# Stimulation of *ipt* overexpression as a tool to elucidate the role of cytokinins in high temperature responses of *Arabidopsis thaliana*

**DOI:** 10.1093/jxb/erw129

**Published:** 2016-04-05

**Authors:** Jan Skalák, Martin Černý, Petr Jedelský, Jana Dobrá, Eva Ge, Jan Novák, Marie Hronková, Petre Dobrev, Radomira Vanková, Břetislav Brzobohatý

**Affiliations:** ^1^Laboratory of Plant Molecular Biology, Institute of Biophysics AS CR, v.v.i. and Mendel University in Brno, CEITEC – Central European Institute of Technology, Mendel University in Brno, Zemědělská 1, CZ-613 00 Brno, Czech Republic; ^2^Laboratory of MS, Faculty of Science, Charles University, Viničná 7, CZ-128 43 Prague, Czech Republic; ^3^Laboratory of Hormonal Regulations in Plants, Institute of Experimental Botany AS CR, Rozvojová 263, 165 02 Praha, Czech Republic; ^4^Institute of Plant Molecular Biology, Biology Centre AS CR, Branišovská 31/1160, 370 05 České Budějovice, Czech Republic

**Keywords:** Abscisic acid, *Arabidopsis thaliana*, cytokinin, heat stress, isopentenyltransferase, proteome.

## Abstract

Cytokinins (CKs) are phytohormones regulating plant growth and development as well as response to the environment. In order to evaluate their function in heat stress (HS) responses, the effect of CK elevation was determined during three types of HS – targeted to shoots, targeted to roots and applied to the whole plant. The early (30min) and longer term (3h) responses were followed at the hormonal, transcriptomic and proteomic levels in Arabidopsis transformants with dexamethasone-inducible expression of the CK biosynthetic gene *isopentenyltransferase* (*ipt*) and the corresponding wild-type (Col-0). Combination of hormonal and phenotypic analyses showed transient up-regulation of the CK/abscisic acid ratio, which controls stomatal aperture, to be more pronounced in the transformant. HS responses of the root proteome and Rubisco-immunodepleted leaf proteome were followed using 2-D gel electrophoresis and MALDI-TOF/TOF. More than 100 HS-responsive proteins were detected, most of them being modulated by CK increase. Proteome and transcriptome analyses demonstrated that CKs have longer term positive effects on the stress-related proteins and transcripts, as well as on the photosynthesis-related ones. Transient accumulation of CKs and stimulation of their signal transduction in tissue(s) not exposed to HS indicate that they are involved in plant stress responses.

## Highlight

Decisive role for cytokinins in stimulation of transpiration during the early heat stress response was demonstrated. Longer term positive effects of cytokinins were associated with elevation of stress- and photosynthesis-related proteins and transcripts.

## Introduction

Due to their sessile character, plants have had to evolve complex systems to sense and respond dynamically to various stress conditions. Heat stress (HS), i.e. transient elevation of the temperature by at least 10 °C above the ambient one, represents one of the most frequent abiotic stresses (Wahid *et al.*, 2007). Integration of environmental stimuli, signal transduction and the stress response is mediated, at least partially, by intensive cross-talk among plant hormones (Wahid *et al.*, 2007). With the exception of abscisic acid (ABA), the mechanism of the hormonal mode of actions in abiotic stress responses needs to be elucidated in more detail. Hormone functions in the HS response can be deduced from the impact of their elevated content on plant stress tolerance. In addition to salicylic acid (SA), a positive effect on HS tolerance was found in the case of cytokinins (CKs), hormones associated predominantly with stimulation of cell division, delay of senescence and stabilization of the photosynthetic machinery (Ha *et al.*, 2012; Stirk *et al.*, 2012; Wang *et al.*, 2012).

Exogenous application of benzyladenine was reported to alleviate the inhibitory effect of HS on photosynthetic activity, chlorophyll level and chloroplast development (Caers *et al.*, 1985). Application of *trans*-zeatin riboside mitigated root mortality, diminished membrane electrolyte leakage, delayed chlorophyll decrease (Liu and Huang, 2002) and enhanced activity of the antioxidant system (Xu and Huang, 2009) under HS conditions . Exogenous CK was found to up-regulate heat-shock proteins (Veerasamy *et al.*, 2007). Similar effects on HS tolerance were achieved by elevation of endogenous CK levels by over-expression of the CK biosynthetic gene *isopentenyltransferase* (*ipt*; Lochmanová *et al.*, 2008; Černý *et al.*, 2013). HS-induced CK production in *HSP70:ipt* tobacco transformants resulted in the up-regulation of one small heat shock protein as well as of ABA- and wound-inducible glycine-rich proteins (Harding and Smigocki, 1994). The *ipt* over-expression driven by the *SAG12* promoter prevented chlorophyll loss during HS, maintaining root production and elongation (Xu *et al.*, 2009).

Plant HS responses include very fast effects associated with prevention of sudden elevation of leaf temperature and subsequent protein denaturation, as well as longer term effects related to plant acclimation. Plants respond to HS by reprogramming their transcriptome, proteome, metabolome and lipidome in order to establish a new steady-state balance of metabolic processes (Mittler *et al.*, 2012; Narayanan *et al.*, 2016). Several transcriptome studies of HS effects have been performed (Rizhsky *et al.*, 2002; Lim *et al.*, 2006; Larkindale and Vierling, 2008), but none of them was focused specifically on the effect of CKs.

As mRNA abundance need not provide relevant information about protein levels, especially in the case of low copy number mRNAs (Rose *et al.*, 2004), proteome analyses are very important for characterization of HS effects (Ferreira *et al.*, 2006; Lee *et al.*, 2007; Huang and Xu, 2008; Černý *et al.*, 2011). The effect of CKs on proteome changes associated with the HS response was studied in *Agrostis stolonifera* either after application of exogenous CK (Jespersen and Huang, 2015) or in transformants with the *ipt* gene under the control of the *SAG12* or *HSP18* promoter (Xu *et al.*, 2010). These studies were focused on longer term CK effects (after 28- and 10-day HS, respectively). The use of the *SAG12:ipt* transformant did not allow following of the impact of CKs on the early stress responses, as the stimulation of CK production was induced gradually during the stress progression.

In order to study CK functions in early HS responses, the dexamethasone-inducible system, which enables elevation of CK content in the physiological range and just before HS application, was chosen. This system allows the following of specific CK-induced changes in protein abundance or protein modification (e.g. phosphorylation and dephosphorylation), which may play an important role in the initial phase of the HS response (Černý *et al.*, 2011, 2014). Taking into account tissue specificity of the response as well as the impact of HS targeting, early responses of leaves and roots were compared after exposure to three types of HS, namely application of HS only to shoots (HS-S), only to roots (HS-R) and to the whole plant (HS-SR), in the system established by Dobrá et al. (2015).

## Materials and methods

### Plant material, cultivation and stress conditions

Seeds of *Arabidopsis thaliana* ecotype Columbia (Col-0) and transgenic line *CaMV35S*>GR>*ipt* (pOp^BK^-*ipt*; *ipt*; Craft *et al.*, 2005) were vernalized for 2 days (4 °C, dark) and cultivated hydroponically (with quarter strength Hoagland medium) for 4 weeks with an 8/16h photoperiod at 130 µmol m^−2^ s^−1^, temperature 20 °C and relative humidity ca 75%. One day before HS application, *ipt* expression was induced in the transformants with 20 µM dexamethasone (DEX) applied to the hydropony medium (in order to ensure equal distribution within the plants via the vascular tissue). The stimulation of *ipt* expression by DEX was confirmed by *ipt* transcript determination in all transformant samples. No *ipt* expression was detected in wild-type plants, even after application of DEX.

Applications of three types of HS [targeted only to shoots (HS-S), targeted only to roots (HS-R) and exposure of the whole plants (HS-SR); Dobrá *et al.*, 2015] were carried out in a Sanyo MLR 350H climate chamber (HS-S: incubator temperature of 40 °C, growth medium at 20 °C; HS-R: incubator of 20 °C, medium pre-heated to 40 °C; HS-SR: growth medium and incubator pre-heated to 40 °C). HS targeting was enabled by efficient insulation of the cultivation vessels containing the hydropony medium (4.25 l), which allowed the initial medium temperature (20 or 40 °C) to be maintained with minor changes (within 5%) for 3h.

Samples were collected in replicates from at least two independent biological experiments; each contained *n*>10 plants in two intervals of HS treatment (after 30min and 180min of HS exposure). The samples were immediately frozen in liquid nitrogen.

### Plant hormone determination

Plant hormones were purified and analysed according to Dobrev and Kamínek (2002) and Dobrev and Vankova (2012). Internal standards (10 pmol per sample) were added: [^3^H_6_]ABA, [^2^H_5_]*trans*Z, [^2^H_5_]*trans*ZR, [^2^H_5_]*trans*Z7G, [^2^H_5_]*trans*Z9G, [^2^H_5_]*trans*ZOG, [^2^H_5_]*trans*ZROG, [^2^H_5_]*trans*ZRMP, [^2^H_3_]DHZ, [^2^H_3_]DHZR, [^2^H_3_]DHZ9G, [^2^H_6_]iP, [^2^H_6_]iPR, [^2^H_6_]iP7G, [^2^H_6_]iP9G and [^2^H_6_]iPRMP (Olchemim, Czech Republic). Methanol/water/formic acid (15/4/1, v/v/v) extract was purified using an SPE-C18 column (SepPak-C18, Waters) and separated using a reverse phase–cation exchange SPE column (Oasis-MCX, Waters). CKs were quantified using a hybrid triple quadrupole/linear ion trap mass spectrometer (3200 Q TRAP, Applied Biosystems). ABA was determined using 2D-HPLC according to Dobrev *et al.* (2005). Replicates from at least two independent experiments were analysed.

### RNA extraction, cDNA synthesis and qRT PCR

RNA was extracted with the RNeasy Plant Kit (Qiagen) and treated with DNAse I (DNA-free; Ambion). For RT, oligo dT primers, Protector RNase Inhibitor (Roche) and M-MLV Reverse Transcriptase RNase H Minus, Point mutant (Promega) were used. Quantitative PCR was performed using the LightCycler® 480 SYBR Green I Master kit (Roche Applied Science) or the UPL system (Roche Applied Science) and LightCycler® 480 Probes Master kit (Roche Applied Science), as described in Dobrá *et al.* (2015). Primer sequences are shown in Supplementary Table S1 at *JXB* online. The cDNA derived from the calibrator RNA was included in each LightCycler run to correct for run-to-run differences. Transcripts levels were normalized against *UBQ10* (*At4g05320*). Quality of RNA and PCR products was verified by agarose gel electrophoresis. Replicates from two independent experiments were analysed.

### Proteome analysis

Total proteins from the roots and leaves were extracted with acetone–trichloracetic acid (Černý *et al.*, 2011; Baldrianová *et al.*, 2015); leaf extracts were subsequently depleted of the majority of the Rubisco protein using the IgY/Rubisco Spin Column. Proteins were resolved by 2-DE (Černý *et al.*, 2011, 2014), and the spot pattern was analysed using Decodon Delta 2D software (http://www.decodon.com). Responses to stimuli (HS/modulated CK pool) of proteins corresponding to detected spots were deemed significant if there was an absolute stimulated/mock spot volume ratio ≥1.35, with *t*-test *P* value <0.05, and similar profiles in two biological replicates (three technical replicates per sample). Selected protein spots were digested with trypsin and analysed using a 4800 Plus MALDI TOF/TOF analyser (AB Sciex; Nd:YAG laser 355nm), and the resulting spectra were searched by local Mascot v. 2.1 (Matrix Science) against the TAIR10 Arabidopsis database (for details see Černý *et al.*, 2013; Baldrianová *et al.*, 2015).

### Thermal analysis

Thermal analysis of shoots was performed with an infrared thermometer, the IRI 4000 series Thermal Imager (http://www.irisys.co.uk; emissivity 0.98). Plant images captured during the application of specific HS were analysed using Irisys 4000 Series Imager software. The overall analysis of leaf temperature at 10 position points was performed for each of the 24 plants in at least two biological replicates for each type of HS.

### Stomatal conductance measurement

Stomatal conductance for water vapour (*g*_s_) was determined using the Li-6400 (Li-Cor, Lincoln) with a leaf chamber of 2×3cm and an integrated light source (LI-6400-02B) at relative humidity ~60–70%, as described in Macková *et al.* (2013). The ambient CO_2_ concentration was maintained at 380 μmol mol^–1^. Measurements were recorded for three plants of each genotype.

### Data analysis

Hierarchical clustering analysis by Gene Cluster 3.0 (http://bonsai.hgc.jp/~mdehoon/software/cluster/) and principal component analysis (PCA) by XLSTAT (http://www.xlstat.com/es/) were used to organize profiles of responsive protein spots, proteins, hormones and metabolites as well as for analysis of variance with the Student–Newman–Keuls test for data comparison. Java TreeView 1.1.4r3 (http://jtreeview.sourceforge.net) was used to view the clustering results generated by Cluster 3.0. Information about protein or metabolite function(s) was collected from the UniProt database, the UniGene database (http://www.ncbi.nlm.nih.gov/unigene), the TAIR database (http://www.arabidopsis.org), a conserved domains search (http://www.ncbi.nlm.nih.gov/Structure/index.shtml), a homology search (http://blast.ncbi.nlm.nih.gov/Blast.cgi), the Kyoto Encyclopedia of Genes and Genomes (http://www.kegg.jp/kegg/), and the literature.

## Results

**Fig. 1.**
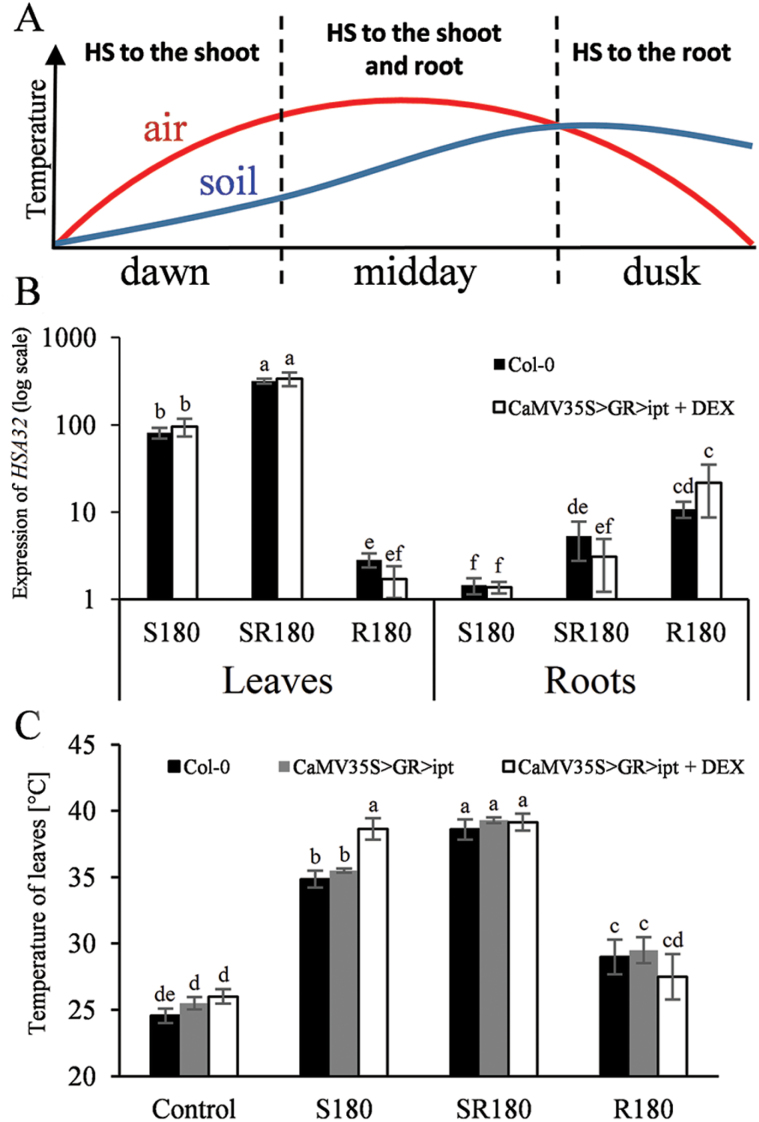
(A) Time course of air and soil temperature profile during a summer day in nature. (B) Transcript levels of heat stress marker gene *HSA32* in leaves and roots. (C) Leaf temperature of wild-type (Col 0), non-induced, and DEX-induced *ipt* transformant after prolonged HS exposure. HS (40 °C) treatment was applied to the shoots (S), whole plant (SR) or roots (R). HS duration was 180min. Unstressed organs were held at 20 °C. Transcript results represent mean values±SD from three independent biological replicates with three technical replicates for each. Leaf temperature data represent means±SD (*n*≥50). Statistical differences are indicated by letters.

The application of heat stress (HS) to the different parts of the plant, which may resemble the temperature profile during a summer day (Fig. 1A), allowed us to evaluate the physiological responses of HS-exposed and non-exposed tissues. In order to check the efficiency of HS targeting, the expression level of the HS marker gene *HSA32* was followed in leaves and roots (Fig. 1B). The almost two orders of magnitude difference between *HSA32* expression in HS-exposed and non-exposed tissues confirmed the specificity of the applied HS treatments. The lower expression of *HSA32* in roots of the DEX-induced transformants after application of HS to the whole plant (HS-SR) may indicate their higher stress tolerance.

In parallel, the leaf temperature of wild-type (Col-0), DEX-non-induced transgenics and transgenic plants over-expressing *ipt* was measured (Fig. 1C). This parameter correlated well with the *HSA32* expression in leaves. Interestingly, a significant difference between the leaf temperature of wild-type and DEX-induced transgenic plants was observed in the case of prolonged HS-S, with no difference being found after HS-SR.

### Hormone analysis – the impact of enhanced *ipt* expression on cytokinin levels and the HS responses

**Fig. 2.**
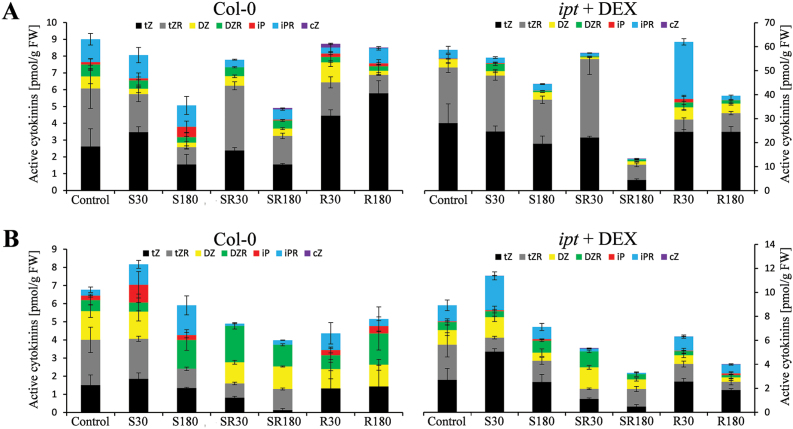
The content of active cytokinins in leaves (A) and roots (B) after exposure to different HS treatments in wild-type (Col-0) and DEX-induced trasngenic plants. Data represent means±SD from two independent biological experiments with three replicates for each bar. tZ, *trans*-zeatin; tZR, *trans*-zeatin riboside; DZ, dihydrozeatin; DZR, dihydrozeatin riboside; iP, isopentenyladenine; iPR, isopentenyladenosine; cZ, *cis*-zeatin. S30, 30-min HS applied to shoots; S180, 180-min HS applied to shoots; SR30, 30-min HS applied to shoots and roots; SR180, 180-min HS applied to shoots and roots; R30, 30-min HS applied to roots; R180, 180-min HS applied to roots HS.

The 24-h activation of *ipt*-inducible plants by DEX led to a ca eight-fold increase of active CKs [*trans*-zeatin (tZ), isopentenyladenine (iP), dihydrozeatin (DZ) and the corresponding ribosides (tZR, iPR and DZR)] in leaves (Fig. 2). The levels of *cis*-zeatin (cZ) and its riboside were generally close to the detection limit. In transformant leaves, a high elevation of CK precursors (CK phosphates) was found, predominantly due to *trans*-zeatin riboside phosphate. Stimulation of *ipt* expression resulted also in strong promotion of CK deactivation by O- and N-glucosylation (see Supplementary Fig. S1, Supplementary Table S2), which indicates an attempt to minimize the effect of *ipt* over-expression on CK homeostasis. Only minor changes were observed in roots.

In wild-type leaves, HS-S led to a transient mild increase of tZ. HS-SR resulted in a fast decrease of active CKs, after 30min especially at the expense of iPR, later on also of tZ and tZR. In contrast, HS-R had a longer term positive effect on tZ content in wild-type leaves. In transformant leaves, HS-S led to the mild gradual decrease of tZ (still well above the wild-type levels). Prolonged HS-SR resulted in strong down-regulation of active CKs. HS-R was associated with a decrease of tZR content, while the levels of iP and DZR transiently increased after 30min. In roots, the HS-S resulted in highly significant transient up-regulation of active CKs [predominantly of tZ, iP(R) and DZ] in both genotypes. HS-SR had the strongest negative effect on active CKs, especially after a prolonged period. The HS-R led in roots to a decrease of active CKs in both genotypes, caused mainly by tZR down-regulation.

### The effect of the HS and *ipt* over-expression on abscisic acid content and CK/ABA ratio

**Fig. 3.**
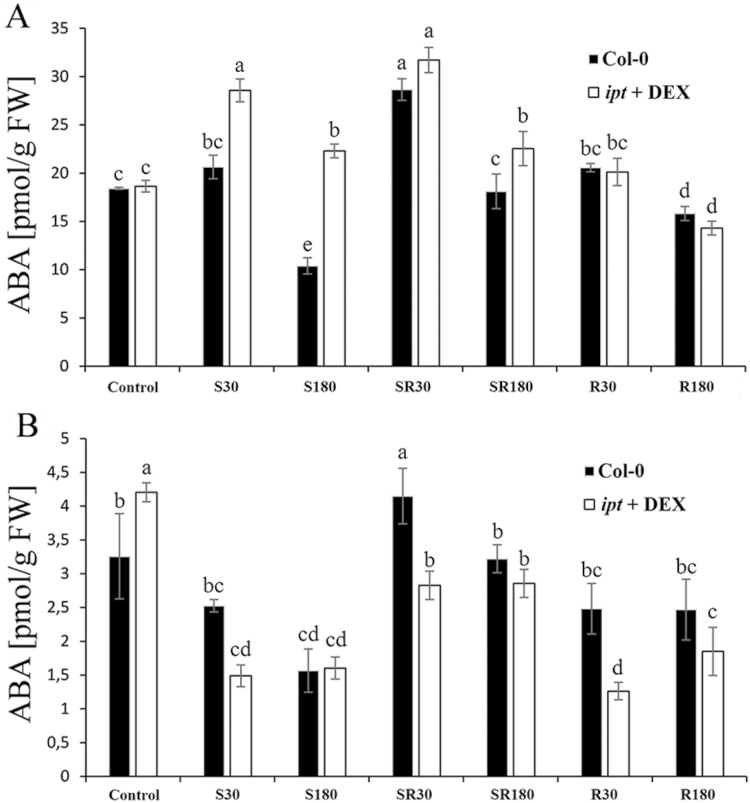
The content of abscisic acid in leaves (A) and roots (B) after exposure to different HS in wild-type (Col-0) and DEX-induced trasngenic plants. HS specification is as described in Fig. 2. Statistical significance of the data was evaluated by ANOVA and Duncan’s multiple range test.

CKs exhibit during the HS response an intensive cross-talk with ABA. Under control conditions, stimulation of *ipt* expression in Arabidopsis did not affect ABA levels in leaves; it caused significant elevation only in roots (Fig. 3). In leaves, HS-S resulted in significant ABA increase only in the transformant, probably due to the necessity of precise regulation of CK/ABA ratio. HS-SR represented the most severe stress, associated with transient ABA increase. During prolonged HS, ABA levels in leaves decreased, showing a similar trend in wild-type and transformant plants. Only a mild, transient increase was observed in leaves after exposure to HS-R. ABA levels in roots were in comparison with leaves considerably lower. In the HS non-exposed roots, an ABA decrease was observed in both genotypes, earlier in the transformant. HS-SR resulted in significant transient elevation of ABA in wild-type roots. Lack of effect in the transformant may be related to lower stress sensing at elevated CK content. HS-R had a negative effect on ABA root content in both genotypes, significantly stronger in the transformant.

The CK/ABA ratio regulates stomatal aperture. In order to check the impact of stimulation of *ipt* expression on regulation of transpiration during HS response, stomatal conductance was compared in wild-type and *ipt* transformant plants (see Supplementary Fig. S2). For technical reasons (size of measuring chamber), tobacco plants (SR1 *Petit Havana*; *CaMV35S*>GR>*ipt*, line 303; Šámalová *et al.*, 2005; Novák *et al.*, 2013) had to be used. DEX-induced CK increase enhanced the speed of opening of stomata as well as their aperture under HS (Supplementary Fig. S2).

### The effect of HS and *ipt* over-expression on cytokinin and abscisic acid related genes

**Fig. 4.**
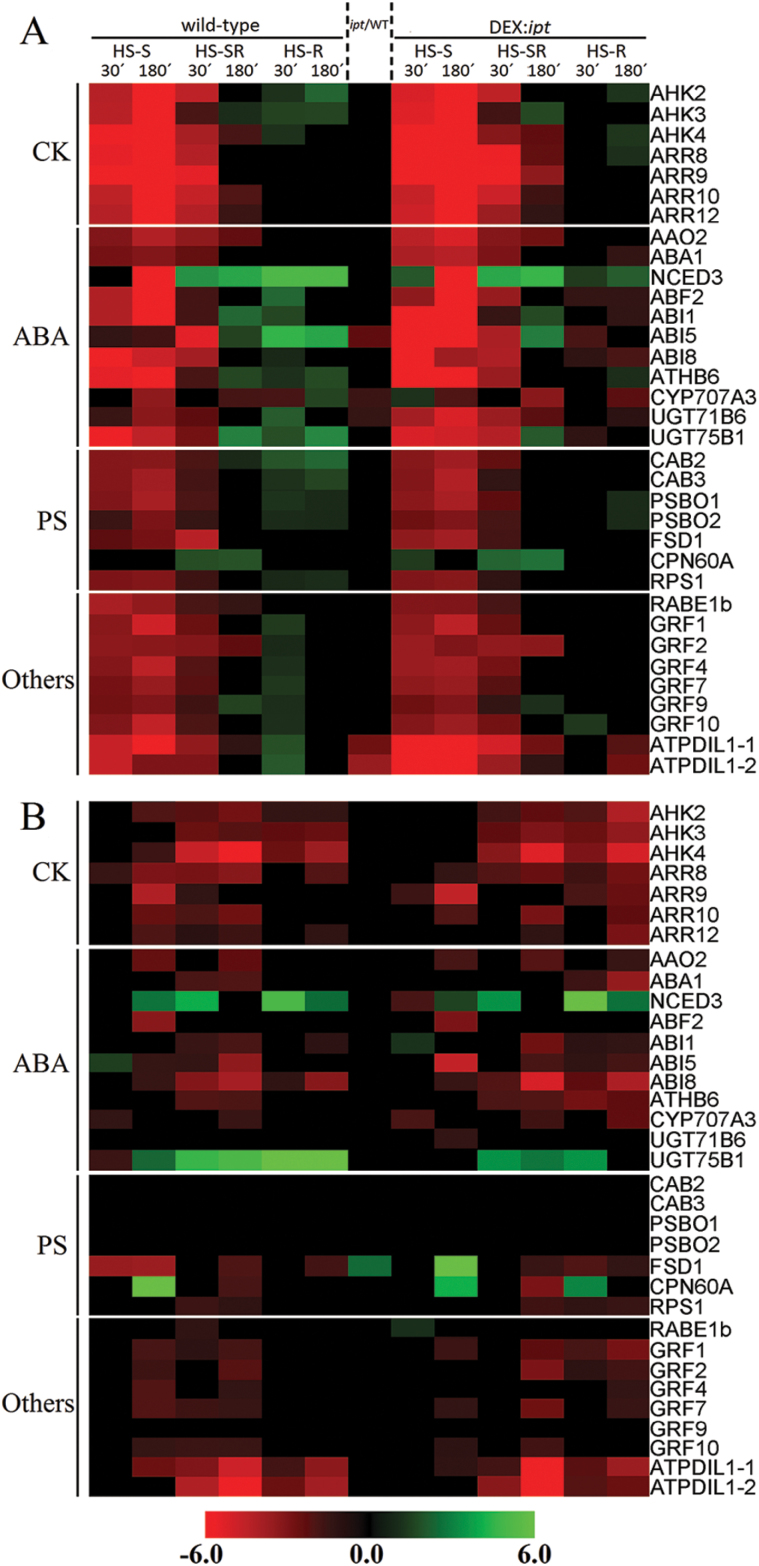
Effect of heat stress targeting on expression profiles of 46 selected hormone- or stress-related genes. The heat map represents fold changes in the abundance of gene transcripts in leaves (A) and roots (B) after heat stress (40 °C) targeted to shoots (S), roots (R) and whole plant (SR) compared with the control. HS duration was 30 or 180min (as indicated). The genes are grouped according to their function (cytokinin, abscisic acid, photosynthesis and stress related).

HS-S was associated in leaves of both genotypes with down-regulation of the expression of genes for CK receptors *AHK2*, *AHK3* and *AHK4* as well as for selected response regulators (type-A: *ARR8*, *ARR9*; type-B: *ARR10*, *ARR12*) (Fig. 4A). In the roots, minor up-regulation of the expression of *AHK2* (by 17%) and *AHK3* (by 11%) as well as of the down-stream signalling component *ARR12* (by 12%) was observed after 30min of HS in the wild-type (see Supplementary Table S3). These minor differences were consistent, but below the threshold of the heat map resolution. In transformant roots, the expression of *AHK2* was increased (by 24%), that of *AHK3* was not affected, both *ARR10* and *ARR12* were elevated (by 16% and 42%, respectively), and *ARR8* was elevated (by 14%) by HS-S. *ARR9* exhibited increased expression in DEX-induced transformant already in control conditions (by 58%).

HS-SR resulted in fast, transient down-regulation of CK signalling components in leaves of both genotypes. In roots, a decrease in expression was generally found.

HS-R resulted in wild-type leaves in the stimulation of the expression of all CK receptors. Enhanced expression was maintained in the case of *AHK2* and *AHK3*. During prolonged HS, expression of *ARR12* and *ARR9* was elevated. All three receptor transcripts were up-regulated during the HS progression in the transformant leaves. Type-A response regulators were increased with delay (after 3h). In the HS-exposed roots, all components of the CK signalling pathway were down-regulated.

The expression of ABA biosynthesis genes (*AAO2*, *ABA1*, *NCED3*) was decreased in wild-type leaves during HS-S (Fig. 4A, Supplementary Table S3). The temporary increase of *NCED3* in the transformant may reflect the necessity to re-establish, upon stimulation of *ipt* transcription, an optimal ratio between ABA and active CKs. The expression of ABA responsive genes (*ABF2*, *ABI1*, *ABI5*, *ABI8*, and *AtHB6*) was down-regulated in both genotypes. The deactivating genes (hydroxylase *CYP707A3* and glucosyltransferases *UGT71B6* and *UGT75B1*) were transiently suppressed in wild-type. In the transformant, elevated expression of hydroxylase might be necessary for precise regulation of ABA upon *NCED3* increase. In roots, transient up-regulation of ABA-related genes *NCED3* and *ABI8* was found in wild-type (Fig. 4B). With the exception of *NCED3* and *ABI1*, ABA-responsive genes were down-regulated upon CK increase.

HS-SR led generally to a short, transient decrease of ABA-related genes in leaves of both genotypes. In roots, strong up-regulation was found only in the case of *NCED3*; prolonged stimulation of deactivating gene *UGT75B1* followed.

HS-R resulted in fast up-regulation of the expression of ABA-related genes in wild-type leaves. This elevation was not observed in the transformant. Increased CK levels might delay this stress response. Fast increase of glucosyltransferases *UGT71B6* and *UGT75B1*, followed by elevation of hydroxylase *CYP707A3*, was detected only in wild-type leaves. In the stressed roots, up-regulation of *NCED3* and deactivating gene *UGT75B1* was found in both genotypes.

### The effect of HS and *ipt* stimulation on the expression of photosynthesis-related, 14-3-3 and other HS-responsive genes

DEX-induced *ipt* expression was associated in control conditions with elevation of *FSD1* expression in the transformant roots, which might indicate the positive effect of enhanced CK content on the antioxidant system. HS-S strongly affected photosynthesis-related genes, e.g. chlorophyll a–b binding proteins 2/3 (*CAB2* and *CAB3*), oxygen-evolving enhancer proteins 1-1 and 1-2 (*PSBO1*, *PSBO2*) and chloroplast superoxide dismutase (*FSD1*). The expression of these genes was suppressed in HS-treated leaves of both genotypes (after 30 and 180min) (Fig. 4A). The exception was chaperonin 60α (*CPN60A*), which is involved in proper folding of Rubisco and thus in its stabilization. Its expression was elevated already after 30-min HS and remained well above the basal level after 3h.

HS-SR resulted in fast down-regulation of this group of genes, followed by their increase during prolonged HS, probably due to at least partial acclimation.

In the non-exposed leaves (HS-R), all tested photosynthesis-related genes were up-regulated in leaves of both genotypes. In roots, the expression of photosynthesis-related genes was very low (Fig. 4B). A similar expression pattern to photosynthetic genes was exhibited by translation elongation factor (*RABE1b*) and ribosomal protein (*RPS1*).

HS significantly affected expression of genes coding for regulatory 14-3-3 proteins (GRF1, 2, 4, 7, 9, 10). The basal level of most *GRF*s was slightly higher in the transformant leaves and roots in comparison with wild-type. HS-S had a negative effect on the expression of all *GRF*s in leaves of both genotypes. In the non-exposed roots, mild transient elevation was found after 30-min HS.

Similarly, in the case of HS-R, the non-exposed leaves showed a transient increase of *GRF* expression. In roots, down-regulation of transcription was observed in the transformant (with the exception of *GRF9* and *GFR10*).

HS-SR resulted in the decrease of *GRF*s in roots of both genotypes, but in leaves only transiently.

HS-R led to a significant elevation of the leaf expression of protein disulfide isomerase (*ATPDIL1-1*, *-2*) as well as glycine-rich RNA-binding protein 7 (*GRP7*), especially in the wild-type.

### Proteomic analysis – identification of temperature- and/or cytokinin-responsive proteins in leaves and roots

**Fig. 5.**
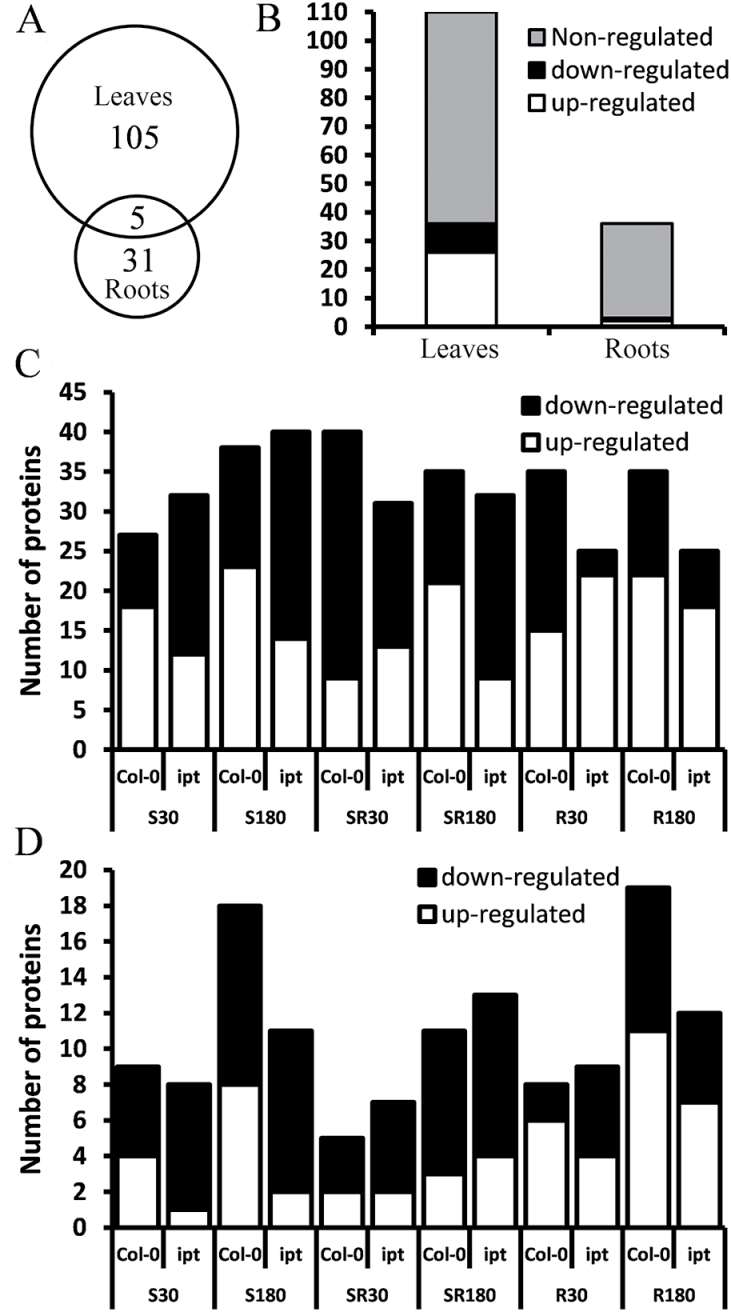
(A) The number of proteins regulated by HS in leaf and root proteome. (B) Effects of activation of *ipt* system (cytokinin elevation) on HS-responsible proteins under control conditions (20 °C). (C) Number of up/down-regulated protein spots in leaves of wild-type (Col-0) and DEX-induced *ipt* transformant after different HS-treatments compared with mock samples. Number of up/down-regulated protein spots in roots of wild-type (Col-0) and DEX-induced *ipt* transformant after different HS-treatments (D). HS specification is as described in Fig. 2.

In order to follow molecular aspects of specific plant responses of the HS-exposed and non-exposed tissues, root and leaf proteome analysis was performed. To increase proteome coverage, we employed standard denaturative acetone–TCA extraction and Rubisco immunodepletion of root and leaf tissues, respectively. 2-DE analysis revealed 146 protein spots with at least 1.35-fold absolute variation caused by CK elevation and/or HS. Altogether, 133 CK- and/or HS-responsive proteins were identified in the 146 spots, including one protein mixture, by MALDI-TOF/TOF MS analysis followed by Mascot database searches against the TAIR 10 database (see Supplementary Tables S4, S5). HS treatments affected 141 spots (Fig. 5A). The overlap of the differentially abundant proteins/proteoforms between leaves and roots was rather limited. In total only five proteins were detected in both datasets, representing 5% and 14% of all detected differentially abundant proteins in leaves and roots, respectively. The 24-h treatment of the transformants with DEX, which resulted in high elevation of endogenous CKs, had a significant impact on the heat-responsive proteins even under control conditions, causing in leaves a change in abundance of 39 spots (Fig. 5B, Table 1).

**Table 1.** List of CK-responsive proteins identified in leaves and roots The *CaMV35S*>GR>*ipt* transformant was induced with DEX for 24h.

Spot/ protein no.^*a*^	AGI (TAIR)^*b*^	Name (Uniprot)^*c*^	*ipt*/Col-0 Mean±SD	Biological function (TAIR; Uniprot)
Leaves: up-modulation by CKs				
S01	At4g02520	Glutathione *S*-transferase PM24	1.52±0.06	Auxin-activated signalling pathway
S02	At3g28940	AIG2 protein-like	1.81±0.04	Indoleacetic acid biosynthetic process
S08	At5g59880	Actin-depolymerizing factor 3	1.54±0.02	Actin filament depolymerization
S09	At1g10960	Ferredoxin-1, chloroplastic	1.59±0.02	Electron transport chain
S11	At2g30860	Glutathione *S*-transferase PHI 9	1.51±0.03	Conjugation of reduced glutathione
S16	At4g25100	Superoxide dismutase [Fe], chloroplastic	2.46±0.05	Destruction of superoxide anion radicals
S26	At1g20020	Ferredoxin--NADP reductase, leaf isozyme 2, chloroplastic	1.91±0.05	Regulation of electron flow
S29	At5g04590	Sulphite reductase	1.51±0.01	Assimilatory sulfate reduction pathway
S30	At5g66190	Ferredoxin--NADP reductase, leaf isozyme 1, chloroplastic	1.39±0.05	Regulation of electron flow
S35	At2g21330	Fructose-bisphosphate aldolase	1.64±0.07	Carbohydrate degradation
S37	At3g14210	GDSL esterase/lipase ESM1	1.38±0.23	Inhibition of nitrile production
S39	At1g68010	Glycerate dehydrogenase HPR, peroxisomal	1.49±0.06	Mediation of fatty acid β-oxidation
S42	At2g43750	Cysteine synthase, chloroplastic/chromoplastic	1.56±0.02	Cysteine biosynthesis
S44	At3g55800	Sedoheptulose-1,7-bisphosphatase, chloroplastic	1.36±0.02	Calvin cycle
S59	At3g01500	β-Carbonic anhydrase 1, chloroplastic	1.46±0.07	Carbon utilization
S76	At3g55800	Sedoheptulose-1,7-bisphosphatase, chloroplastic	1.58±0.03	Calvin cycle
S77	At1g09750	Nucleoid DNA-binding-like protein	1.42±0.03	DNA-binding
S78	At1g56340	Calreticulin-1	1.43±0.03	Calcium ion homeostasis
S83	NA^*d*^	Not identified	1.82±0.02	NA^*d*^
S85	At4g02520	Glutathione *S*-transferase PM24	1.38±0.04	Auxin-activated signalling pathway
S86	At2g37220	Ribonucleoprotein At2g37220, chloroplastic	1.67±0.09	mRNA processing
S89	At1g19130	Putative uncharacterized protein At1g19130/F14D16_18	1.55±0.01	Unknown
S93	At3g63140	Chloroplast stem-loop binding protein of 41kDa, chloroplastic	1.6±0.06	rRNA processing
S103	At1g70730	Probable phosphoglucomutase, cytoplasmic 2	1.8±0.01	Carbohydrate metabolism
S108	At3g15356	Lectin-like protein LEC	1.99±0.03	Ethylene and jasmonic acid signalling pathway
S109	At1g21750	Protein disulfide isomerase-like 1-1	2.32±0.02	Cell redox homeostasis
Leaves: down-modulation by CKs				
S07	At3g60750	Transketolase-1, chloroplastic	0.66±0.12	Reductive pentose-phosphate cycle
S22	At1g06680	Oxygen-evolving enhancer protein 2-1, chloroplastic	0.66±0.03	Regulation of photosystem II
S24	At4g28520	12S seed storage protein CRU1 (Cruciferin 1 or C)	0.47±0.02	Seed storage protein
S34	At2g28000	Chaperonin 60 subunit α1, chloroplastic	0.71±0.04	Chloroplast development
S43	At3g16640	Translationally controlled tumour protein homologue	0.57±0.03	Auxin homeostasis
S47	At2g40490	Uroporphyrinogen decarboxylase 2, chloroplastic	0.54±0.03	Chlorophyll biosynthesis
S54	At4g09010	Thylakoid lumenal 29kDa protein, chloroplastic	0.53±0.05	Peroxidase family
S70	At4g29060	Elongation factor Ts	0.74±0.01	Protein biosynthesis
S84	At1g30580	Obg-like ATPase 1	0.64±0.03	Hydrolysis of ATP
S101	AtCg00490	Ribulose bisphosphate carboxylase large chain	0.68±0.02	Calvin cycle
Roots: up-modulation by CKs				
R09	At4g13930	Serine hydroxymethyltransferase 4	1.5±0.03	l-Serine metabolic process
R20	At4g14630	Germin-like protein subfamily 1 member 8	1.6±0.04	Response to salt stress
Roots: down-modulation by CKs				
R10	At3g09260	β-Glucosidase 23	0.71±0.03	Endoplasmic reticulum body organization

^*a*^ Individual spot number indicating position on 2 DE gels, see Supplementary Fig. S3.

^*b*^ Accession number according to the TAIR database.

^*c*^ Corresponding protein entry name according to the UniProt database.

^*d*^ NA: not available.

### Cytokinins modulate response to temperature

Application of the three types of HS had a huge effect on the leaf (Fig. 5C) and root proteomes (Fig. 5D) in both genotypes. Negative effects prevailed in the case of the most severe HS-SR, in wild-type after 30min, the response being delayed in the transformant (Fig. 5C). Interestingly, CK elevation diminished the stress effects on leaf proteome in the case of HS-R, where the number of down-regulated spots was significantly lower than in wild-type (Fig. 5C, Supplementary Fig. S3). In contrast, a higher number of proteins were down-regulated in the transformant during HS-S progression.

**Fig. 6.**
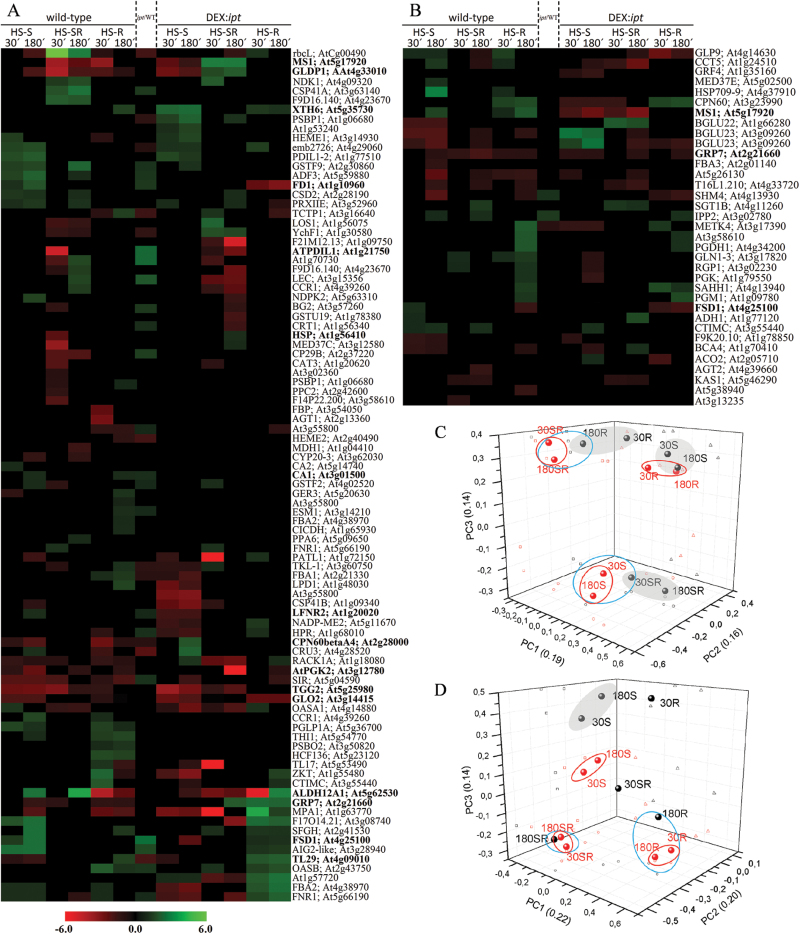
(A, B) Comparison of the effect of different heat stress treatments on HS-responsive proteins in wild-type (Col-0) and DEX-induced *ipt* transformant leaves (A) and roots (B). In total, 146 HS-responsive protein spots were detected. HS specification is as described in Fig. 2. The heat-maps are divided into three sections: on the left side are levels of proteins regulated by HS-S (Col-0 40 °C/Col-0 21 °C), in the middle are levels of proteins regulated only by activation of the *ipt* system (DEX *ipt*/mock Col-0), on the right side are the effects of *ipt* expression after exposure to different HSs compared with HS-treated wild-type plants (DEX 40 °C *ipt*/40 °C Col-0). (C, D) PCA analysis of abundance of differentially regulated proteins in each type of HS treatment in leaves (C) and roots (D) plotted on three principle components, providing 3D plots with visualisation of similarities between specific HS treatment in wild-type (grey circles, dots and letters) and transgenic plants (red circles, dots and letters). The dimensional intersections are marked as rings (PC1×PC2), triangles (PC1×PC3) and squares PC2×PC3.

A comprehensive analysis of the early and late responses of 110 leaf proteins to individual HS treatments in wild-type and transformant plants is shown in Fig. 6A. The response of the root proteome is shown in Fig. 6B. Induction of *ipt* resulted in increased abundance of a number of proteins under control conditions. They included especially photosynthesis- and antioxidant system-related proteins, e.g. chloroplast superoxide dismutase (FSD1, At4g25100), the member of thioredoxin superfamily ATPDIL1 (At1g21750) and chloroplast ferredoxin-NADP(H) oxidoreductase (LFNR2; At1g20020). HS affected abundance of at least three proteins associated with stomatal regulation, myrosinase (TGG2, At5g25980), carbonic anhydrase 1 (CA1, At3g01500) and glycine-rich protein 7 (GRP7, At2g21660). TGG2 was down-regulated in wild-type, especially after the shoot exposure to HS. This suppression was much stronger in the DEX-induced transformant (only during HS-S). CA1 was up-regulated in the transformant under control conditions. CA1 elevation was observed after prolonged HS-S and prolonged HS-R in wild-type. GRP7 was suppressed in stressed wild-type, while it was far more abundant in the transformant, especially after prolonged HS-R and HS-SR. CK elevation increased the abundance of xyloglucan endotransglucosylase/hydrolase 6 (XTH6, At5g65730) during HS-S and HS-R (in wild-type up-regulation only in HS-R was observed). During prolonged HS-S, CKs positively affected abundance of chaperonin CPN60βA4 (At2g28000), involved in Rubisco protection. During HS-R, CKs positively affected abundance of antioxidant enzymes (e.g. ascorbate peroxidase 4, TL29, At4g09010) and protective proteins (e.g. HSP, At1g56410). During strong stress (HS-SR), the transformant exhibited considerably higher levels of protein methionine synthase (MS1, At5g17920), important for ethylene biosynthesis, and glycine decarboxylase (GLDP1, At4g33010), which functions in the regulation of redox state (both with the exception of HS-S). These two above-mentioned proteins were down-regulated in wild-type. Transformant plants exhibited during HS-R low levels of ferredoxin 1 (FD1, At1g10960) and glycolate oxidase (GLO2, At3g14415), both involved in ROS signalling. Unlike wild-type plants, transformants had generally lower level of proline dehydrogenase (ALDH12A1, At5g62530), which may indicate a higher level of proline, which functions also as a ROS scavenger.

The increase of endogenous CKs affected 92% and 93% of temperature-responsive proteins in leaves and roots, respectively. The PCA analysis of differentially abundant leaf and root proteins (Fig. 6C, D, respectively) indicates proximity among responses to individual HS treatments in wild-type and transformant plants.

One of the main protein characteristics is the subcellular localization. In our proteomic analysis, 33–41% of proteins identified in leaves were found to be localized to plastids. Plasma membrane represented the most abundant localization in roots and the second most frequent one in leaves; vacuole localization in leaves and cytosol localization in roots followed (both ~10%; according to the MASC GATOR database; Supplementary Fig. S4). Thus, the majority of plant responses to the temperature increase as well as to the elevated CK content seem to be located in plastids, as predicted (Černý *et al.*, 2011; Černý *et al.*, 2013). The number of HS-regulated proteins localized in plastids was significantly higher after activation of the inducible *ipt* system in transgenic plants.

**Fig. 7.**
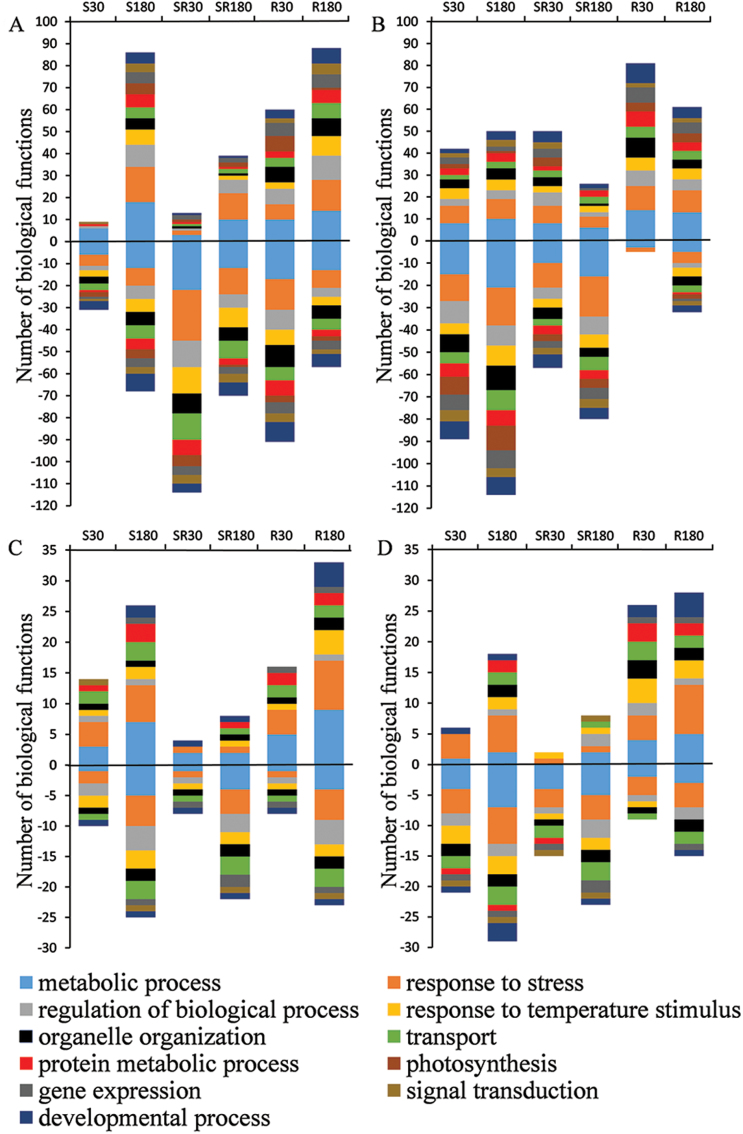
The effect of different HS treatments on the number of up- and down-regulated proteins of different biological functions in wild-type leaves (A), transformant leaves (B), wild-type roots (C) and transformant roots (D). Significant change in relative protein abundance: *P*<0.05. The colour code represents the functional classification acquired from the TAIR database; the biological functions are categorized according to Bevan *et al.* (1998). HS specification is as described in Fig. 2.

In order to obtain an overview of biological functions of proteins affected by HS treatments and CK modulations (Fig. 7), the proteins were sorted according to Bevan *et al.* (1998) and the annotations were provided by the TAIR database. As one protein may have more than one biological function, the total number of affected biological processes is higher than the number of regulated proteins (Fig. 7). The biggest negative effects were observed in wild-type leaves at the early response to HS-SR (Fig. 7A). The acclimation to elevated temperature seems to take place soon after the application of HS-R in wild-type and especially in DEX-induced transformant plants (Fig. 7B). Overall, the largest number of HS-regulated proteins in leaves was involved in metabolic processes (such as carbohydrate metabolism, nucleotide metabolism) and stress responses (see Supplementary Table S6). The number of down-regulated proteins increased during prolonged HS treatments also in root proteome, especially in wild-type (Fig. 7C). HS-SR had a predominantly negative impact. HS-R resulted in an increase in the abundance of proteins, which are mainly involved in metabolic processes, responses to stress and responses to temperature stimulus. CK elevation increased the number of up-regulated proteins involved in the temperature stimulus responses already after 30-min HS-R (Fig. 7D), which suggests a positive role of CKs in the early responses to this HS treatment.

## Discussion

The link between CKs and temperature perception or response has attracted considerable attention. The long-term effects of CK elevation on turf quality and creeping grass proteome responses were studied extensively (Xu *et al.*, 2010; Jespersen and Huang, 2015). Recently, we described the effect of exogenous CK on the proteome of 7-day Arabidopsis seedlings exposed to 15-min HS. We found that more than 70% of the HS-responsive proteins were modulated by CK, which indicated intensive interactions between temperature and CK signalling (Černý *et al.*, 2014). In the present study, we evaluated multiple CK functions in HS responses by determination of the impact of elevated endogenous CK content on the Arabidopsis reactions to different HS treatments (targeted to shoots, roots or the whole plant).

Responses of leaves and roots were followed separately in order to compare the proteomic and transcriptomic changes in tissues that were heat stressed with those not directly affected. Moreover, proteomic and transcriptomic responses exhibit high tissue specificity, as indicated by only minor overlap of leaf and root proteomes (Fig. 5A; Žd’árská *et al.*, 2013).

### Stomatal control during the early heat stress response

HS represents an acute stress. The early HS response is associated with maintenance of leaf temperature lower than that of the environment, at least until other defence mechanisms can be activated. The most effective cooling mechanism is evapotranspiration controlled by stomatal movement (Wahid *et al.*, 2007). Dynamic regulation of stomatal aperture during the early HS response was recently described in tobacco (Macková *et al.*, 2013). Our proteomic analysis (Fig. 6) indicated tight connection between stomatal behaviour and temperature perception. At least three proteins associated with stomatal regulation were affected by HS. Myrosinase, which has a negative effect on stomatal conductance, decreased after application of HS-S, in wild-type and even more in transgenic plants (Fig. 6A, Supplementary Fig. S5A). CA1, which also contributes to stomatal closure, was up-regulated in later stages of the HS response targeted to only part of the plant (HS-S, HS-R) similarly in wild-type and transformant plants. These proteins are associated with ABA signalling (Islam *et al.*, 2009) and according to the STRING database (see Supplementary Fig. S5B) exhibit protein–protein interactions with other HS-responsive proteins identified in our proteomic analysis. In contrast, the stress-related protein GRP7, which decreases ABA levels (Cao *et al.*, 2006) and promotes stomatal opening (Kim *et al.*, 2008), was up-regulated during HS-R and HS-SR in transgenic plants (Supplementary Fig. S5A), but down-regulated in wild-type plants. Strong up-regulation of xyloglucan endotransglucosylase/hydrolase 6, a protein involved in the response to water deprivation, in the transformant predominantly during the HS-S response indicates a decrease of leaf water potential.

Proteomic data are in accordance with the results of hormonal and transcriptomic analyses. High elevation of active CKs and only moderate increase of ABA during HS-S in the transformant indicate that precise regulation of stomatal aperture and thus the early HS response may be impaired by high CK elevation. Indeed, comparison of stomatal conductance of the DEX-induced *ipt* tobacco transformant and the corresponding wild-type plants showed faster and stronger increase of stomatal aperture during HS upon CK elevation (see Supplementary Fig. S2). Our Arabidopsis transcriptomic analysis seems to indicate the tendency of DEX-induced *ipt* plants to re-establish CK/ABA homeostasis. While the expression of ABA biosynthetic genes (*AAO2*, *ABA1*, *NCED3*) was transiently decreased in wild-type leaves, in order to increase the stomatal aperture (Fig. 4A), the transformant exhibited early stimulation of *NCED3.* Down-regulation of ABA-responsive genes was, however, observed in both genotypes. These data correlate well with phenotypic analysis (Fig. 1C). Leaf temperature determination revealed that HS-S led in the transformant after 180min to a significant temperature increase (around 4.0±0.8 °C in comparison with wild-type; Fig. 1C). Our finding resembles effects observed in well-watered *ost1* mutants, which have impaired regulation of stomatal aperture (Ache *et al.*, 2010). These mutants suffered from decreased leaf water potential due to higher conductance of stomata in comparison with the conductance of the root–shoot continuum. In the case of HS-SR, stronger stress severity might help to avoid this problem by higher elevation of ABA and stronger down-regulation of active CKs.

### Positive effect of cytokinins during prolonged HS

CKs were found to exhibit a positive effect on abundance of proteins associated with photosynthesis and the antioxidant system under control conditions (Fig. 6). Also the basal level of a number of stress-related transcripts was higher in transformant leaves and roots in comparison with wild-type, for example of those encoding FSD1 (Fig. 4).

When the impact of CK elevation was followed during HS progression, opposite effects on early and late leaf proteome responses were detected. Longer term positive effects of CKs may be related to their strong link with plastids, the organelles where the majority of HS-responsive proteins are localized. Elevation of endogenous CKs in the transformant increased the number of up-regulated proteins involved in temperature stimulus responses (Fig. 7D). The CK elevation had a positive effect on photosynthesis-related proteins (Fig. 6A), e.g. chaperonin CPN60βA4, involved in proper folding of Rubisco. The enhanced CK content had a positive effect on the antioxidant system, at both transcript and protein levels of chloroplast superoxide dismutase *FSD1* (Figs 4 and 6A).

The data from our proteomic analysis show that DEX-induced transgenic plants have a higher abundance of stress-related proteins. Phosphoglycerate kinase (AtPGK2, At3g12780), a protein associated with enhanced salt stress tolerance (Cheng *et al.*, 2014), was generally maintained in transformant leaves during HS treatments, while its abundance highly decreased in the wild-type, especially during prolonged HS-S.

Our proteomic analysis showed that CKs enhance biosynthesis of heat-shock proteins (e.g. At1g56410; Fig. 6, Supplementary Table S4), which is in accordance with the report of Veerasamy *et al.* (2007). Thus, CKs seem to help plants to overcome negative consequences of HS also via up-regulation of protective proteins. This conclusion is in accordance with the previous reports on positive effect of CK elevation during longer term HS treatment, for example in transgenic creeping bentgrass (Xu *et al.*, 2010).

The impact of CKs may be also deduced from the category of the most abundant proteins. In wild-type roots, catabolic cascades and carbohydrate synthesis were over-represented (see Supplementary Table S5). Especially HS-SR had a negative impact on proteins involved in biological processes. These results are in accordance with the study of Rocco *et al.* (2013), who reported that the two most abundant groups of proteins in Arabidopsis leaves after 6-h HS are those associated with energy production/carbon metabolism and response to abiotic and oxidative stresses. In contrast, DEX-induced transgenic plants exhibited low abundance of proteins associated with catabolic cascades/carbohydrate synthesis, which also indicates a longer term positive effect of CKs on stress tolerance.

### Similarity of the impact of *ipt* over-expression and wild-type HS-R response

Stimulation of CK biosynthesis, resulting in up-regulation of CK levels in the transformant, during HS progression exhibited similar effects on leaf proteome and transcriptome to HS-R treatment of wild-type plants, which showed more than two-fold increase of tZ compared with the control. At the proteome level, the increase of ascorbate peroxidase 4 may be mentioned, while at the transcriptome level transient elevation of *GRF* expression was found. In fact, our hormonal, transcriptiomic and proteomic analyses proved that exposure of roots to HS has in the non-exposed shoots a promoting effect on the pool of active CKs (Fig. 2), as well as on transient up-regulation of the CK signalling pathway. Transcriptomic data showed transient stimulation of the expression of components of the CK signalling pathway (namely of all CK receptors) in wild-type leaves (Fig. 4). Simultaneously, all tested photosynthesis-related genes were up-regulated in the non-exposed leaves of both genotypes. These findings are in accordance with the results of Dobrá *et al.* (2015) as well as Liu and Huang (2005) who reported increase of CKs in shoots of *Agrostis stolonifera* after elevation of soil temperature (35 °C).

The physiological relevance of our experimental set-up (targeted HS) is given by the fact that in nature plant exposure to HS is often not homogeneous. In many cases the temperature of air and soil differs (as shown in Fig. 1A). Thus, our arrangement enables us to follow plant responses to these frequent situations. It seems that plants mobilize their defence on partial stress exposure, before the stress becomes severe (during summer midday). The similarity of transcriptomic and proteomic responses of DEX-induced *ipt* and wild-type plants exposed to targeted HS, as well as hormonal analyses, indicates that CKs contribute to the stress defence.

### Conclusions

The aim of our study has been the characterization of the function of CKs in the perception of an HS stimulus and plant defence. The plants were subjected to three different types of HS in order to compare the impact of partial and total stress conditions. Hormonal, proteomic and transcriptomic analyses confirmed that CKs play a crucial role in the plant responses to specific temperature conditions. Evaluation of the impact of the changed CK/ABA ratio showed that CKs are involved in regulation of stomatal aperture and subsequently transpiration. The importance of precise control of this process was indicated by the HS-responsiveness of proteins involved in the stomatal movement. Additionally, proteome analysis demonstrated that CKs have longer term positive effects on the stress-related proteins, including those that regulate cell redox state, as well as on the photosynthesis-related ones. Comparison of the responses of the HS-exposed and non-exposed tissues, in combination with determination of the effect of elevated CK content, enabled us to demonstrate that transient CK accumulation and stimulation of signal transduction in the non-HS-exposed tissues allows the preservation of plant fitness and may positively affect stress tolerance, with simultaneous suppression of the growth of the stress targeted tissue(s).

## Supplementary data

Supplementary data are available at *JXB* online.

**Figure S1**. Content of cytokinin metabolites in leaves and roots of wild-type and DEX-induced transgenic plants.

**Figure S2**. Stomatal conductance (*g*_s_) in wild-type and *ipt* over-expressing transgenic tobacco leaves during heat stress (40 °C).

**Figure S3**. Average two-dimensional gel electrophoresis maps of leaf and root proteome.

**Figure S4**. Subcellular localization of proteins regulated after exposure to specific HS conditions in wild-type and transgenic plants.

**Figure S5**. The heat-map of proteins regulated in leaves and their functional assignment to temperature-stimulus response and stomatal-movement regulation.

**Table S1**. Primer sequences used in q RT PCR.

**Table S2**. Content of cytokinin metabolites and abscisic acid.

**Table S3**. Expression levels of genes related to cytokinin and abscisic acid metabolism and signalling in wild-type and DEX-induced *ipt* transformant during different HS treatments.

**Table S4**. Complete table of HS-responsive proteins identified in leaves.

**Table S5**. Complete table of HS-responsive proteins identified in roots.

**Table S6**. Number of regulated proteins and their assignment to biological functions.

Supplementary Data
