# Supplementary material for: Stimulation of ipt overexpression as a tool to elucidate the role of cytokinins in high temperature responses of Arabidopsis thaliana
Source: J Exp Bot. 2016 Apr 5;67(9):2861–73. doi: 10.1093/jxb/erw129 (PMC4861028; doi:10.1093/jxb/erw129)
Supplement: Supplementary Data [file supp_67_9_2861__index.html]

Stimulation of ipt overexpression as a tool for elucidation of the role of cytokinins in high temperature responses of Arabidopsis thaliana — Stimulation of ipt overexpression as a tool to elucidate the role of cytokinins in high temperature responses of Arabidopsis thaliana — Supplementary Data 

# Stimulation of *ipt* overexpression as a tool to elucidate the role of cytokinins in high temperature responses of *Arabidopsis thaliana*

## Supplementary Data

Data files

- supplementary\_tables\_S1\_S6\_Figures\_S1\_S5.pdf - Supplementary Data
