# Supplementary material for: Stimulation of ipt overexpression as a tool to elucidate the role of cytokinins in high temperature responses of Arabidopsis thaliana
Source: J Exp Bot. 2016 Apr 5;67(9):2861–73. doi: 10.1093/jxb/erw129 (PMC4861028; doi:10.1093/jxb/erw129)

Journal of Experimental Botany

Stimulation of *ipt*-overexpression as a tool for elucidation of cytokinin role in temperature responses of *Arabidopsis thaliana*

Running title: The impact of cytokinin over-expression on heat stress responses

Jan Skalák, Martin Černý, Petr Jedelský, Jana Dobrá, Eva Ge, Jan Novák, Marie Hronková, Radomira Vanková, Břetislav Brzobohatý

Supplementary data

Table S1. Primers used in q RT PCR.
Sequences start at 5´ end.

Gene Product	*Abbr.*	*Locus*	*Primer*	*Sequence*	*Anneal. Temp.*	*UPL Probe No.*
Heat shock transcription factor A2	*HsfA2*	At2g26150	**At HsfA2 L**	TTTGTGGTGTGGGATTCTCAT	**60^0 C**	#12
			**At HsfA2 R**	CCTTTATAGCTGAAGACTCATAGGAAA		
Heat-stress-associated 32-kD protein	*HSA32*	At4g21320	**At HSA32 L**	TGCTTCGTAGTGGTCCTTCA	**60^0 C**	#25
			**At HSA32 R**	TGTCAAACCCCAACTGCTTA		
Dehydration-responsive element binding protein	*DREB2B*	At3g11020	**At DREB2B L**	GCCAAGTTTTGCAGGAGAAA	**58^0 C**	#35
			**At DREB2B R**	CTGTTGCTGTTCCTGTTGCT		
Isopentenyl transferase 1	*IPT1*	At1g68460	**fAtIPT1**	ACTCAACTTCCACCTCC	**60^0 C**	-
			**rAtIPT1**	ATTCCGTGACCGTGACT		
Isopentenyl transferase 3	*IPT3*	At3g63110	**fAtIPT3**	TTTTCAGGAACGAGCAGT	**60^0 C**	-

			**rAtIPT3**	TTGGACCTTCGCTTTGTA		
Isopentenyl transferase 5	*IPT5*	At5g19040	**fAtIPT5**	ACTTGTTTGCTTGCGTGT A	**60^{0} C**	-
			**rAtIPT5**	CCTTCTTTCGTCATCGTC		
Isopentenyl transferase 7	*IPT7*	At3g23630	**fAtIPT7**	CCG TGC CAC AAA AAG TAA A	**58°C**	-
			**rAtIPT7**	AAC AGT GCT CAA CAT CAT C		
Cytokinin oxidase/ dehydrogenase 1	*CKX1*	At2g41510	**fCKX1-510**	CACCTTTGGCAATTCTACAT	**60^{0} C**	-
			**rCKX1-510**	TGT CCT TGA AGC GAG TGA		
Cytokinin oxidase/ dehydrogenase 3	*CKX3*	At5g56970	**fCKX3-509**	GCTGCGTGGCTATGGA	**60^{0} C**	-
			**rCKX3-509**	TGTGGACCGTACCGAAAC		
Cytokinin oxidase/ dehydrogenase 5	*CKX5*	At5g21482	**fCKX5-513**	GTCAGAAGAACGAGTCAT	**60^{0} C**	-
			**rCKX5-513**	CGCAAGATTGTGGTAGT		
Cytokinin oxidase/ dehydrogenase 6	*CKX6*	At1g75450	**fCKX6-515**	GAC GCC CAA GCC ACT	**60^{0} C**	-
			**rCKX6-515**	TAACTCTCCACCCCATAC		
Arabidopsis histidine kinase 2	*AHK2*	At5g35750	**At AHK2 L**	AAGAACAGCGTGTTGAAGCA	**58°C**	#22
			**At AHK2 R**	GTTTGCTGGCAAGTTGGTG		
Arabidopsis histidine kinase 3	*AHK3*	At1g27320	**At AHK3 L**	CAGATGTTGCAAAGTCACAGTTC	**58°C**	#59
			**At AHK3 R**	CCATAAGCATATGCAACATTCC		

Arabidopsis histidine kinase 4	*AHK4*	At2g01830	**At AHK4 L**	ATTGAGCGGTTACGAAGCTG	**60°C**	#33
			**At AHK4 R**	GCTCCTCAGAGACCAAATGC		
Arabidopsis response regulator 8	*ARR 8*	At2g41310	**At ARR8 L**	ACGTTCCTGCAAGAATCTCC	**60°C**	#17
			**At ARR8 R**	GGTTTCAACTTGGTAAGATCAGC		
Arabidopsis response regulator 9	*ARR 9*	At3g57040	**At ARR9 L**	CGACCCAAATGCGTTTTC	**58°C**	#65
			**At ARR9 R**	CTTGAGCAAATCATAACCAGTCA		
Arabidopsis response regulator 10	*ARR10*	At4g31920	**At ARR10 L**	GCTTCTGATGCTGGTTCCTT	**60°C**	#21
			**At ARR10 R**	CAATCACCTTCCGAGAAATCA		
Arabidopsis response regulator 12	*ARR12*	At2g25180	**At ARR12 L**	CTCCACGATGAAGCAGGAA	**58°C**	#25
			**At ARR12 R**	GAGTGAACTAAACCCTCCATATCC		
Zeaxanthin epoxidase	*ABA1*	At5g67030	**At ABA1 L**	ACAGTACTTTGTTTCTTCGGATGTT	**60°C**	#31
			**At ABA1 R**	TCAAACAACCTTTTCTTCATACCA		
ABA insensitive 1	*ABI1*	At4g26080	**At ABI1 L**	TCGACTATACCAAGATTCCTTCAA	**60°C**	#6
			**At ABI1 R**	GCCAAATGCATCCTCTCTCT		
ABA insensitive 2	*ABI2*	At5g57050	**At ABI2 L**	TTCAGAATCTCTAAATGGCTGAAAC	**60°C**	#61
			**At ABI2 R**	AAAAGGACTAAAATCAGAGTTTCCTG		
ABA insensitive 5	*ABI5*	At2g36270	**At ABI5 L**	CAAGAAGCGGATTCTCTCAGTT	**60°C**	#70

			**At ABI5 R**	TCAACTTCGTTTCTCTAGTTACCATTT		
ABA insensitive 8	*ABI8*	At3g08550	**At ABI8 L**	GCATAATTACATGAAAACTCCCAAT	**60°C**	#25
			**At ABI8 R**	CGCCTGGATGTTAAGTCAGAA		
Aldehyde oxidase 2	*AAO2*	At3g43600	**At AAO2 L**	TTCTTTCAAGAGTGTCAAGCTCA	**60°C**	#88
			**At AAO2 R**	AAGACAGGATCGAACTTGGAGA		
9-*cis*-epoxycarotenoid dioxygenase 3	*NCED3*	At3g14440	**At NCED3 L**	TCGTCGTGATAGGGTCCTG	**60°C**	#22
			**At NCED3 R**	TTCTCGTCAGACTCGTTGAAAA		
ABA 8'-hydroxylase	*CYP707A3*	At5g45340	**At CYP707A3 L**	CGATTTATCGCCGGAGTC	**60°C**	#25
			**At CYP707A3 R**	CGTAAGGATAACCCATTGTTCC		
UDP-glucosyl transferase 71B6	*UGT71B6*	At3g21780	**At UGT71B6 L**	CTCCCAGAAGGGTTTTTCG	**60°C**	#6
			**At UGT71B6 R**	TGTTCAGCCCATCCGATAAC		
UDP-glucosyl transferase 75B1	*UGT75B1*	At1g05560	**At UGT75B1 L**	TGGAAGACTGGTGTGAGGGTA	**60°C**	#22
			**At UGT75B1 R**	TTCCAAACACCTCCTGATCTC		
UDP-glucosyl transferase 75B2	*UGT75B2*	At1g05530	**At UGT75B2 L**	AGAAATGGTGGCAGTTGGTC	**60°C**	#4
			**At UGT75B2 R**	CCTGATTCGCTTCCAGTGA		
Homeobox protein 6	*ATHB6*	At2g22430	**At ATHB6 L**	CCGTGACAATGAATCTCTCCT	**60°C**	#25
			**At ATHB6 R**	GCCGCGTTGTTCTCTTCTT		

Abscisic acid responsive elements-binding factor 2/ Abscisic acid responsive elements-binding protein 2	*ABF2/ AREB1*	At1g45249	**At ABF2 L**	GCACACGAATTTAGCAAAAAGA	**60°C**	#38
			**At ABF2 R**	CATCTCCTGGTGGCTCATTC		
Accelerated cell death	*ACD2*	At4g37000	**At ACD2 L**	GTCGACGGTGGAGAATCG	**60°C**	#12
			**At ACD2 R**	CGTACATCTGGAGGGAGGTT		
Early responsive to dehydration 15	*ERD15*	At2g41430	**At ERD15 L**	TGGTTTCTATGACAACGGAGAG	**60°C**	#22
			**At ERD15 R**	TGGAAGAAGATCAGCTACATCG		
Auxin-induced protein, putative	*ATB2*	At1g60710	**At ATB2 L**	TCGATACTCGTGTCCCTATCG	**58°C**	#13
			**At ATB2 R**	AACAGCATGTGCTCTTCTGATAGT		
Nitrilase 1 (indole-3-acetonitrile nitrile hydratase / indole-3-acetonitrile nitrilase)	*NIT1*	At3g44310	**At NIT1 L**	TGAGTTTCGGAAGTACCATGC	**58°C**	#72
			**At NIT1 R**	CACGTCAGCCAATCTTGCTA		
Malate dehydrogenase, cytoplasmic 1	*MDcyt1*	At1g04410	**At MDcyt1 L**	ACAGCGTTCCATCAGGACTT	**58°C**	#12
			**At MDcyt1 R**	TGATACTTCATCAATCGGAAGG		
Ribosomal protein S1	*RPS1*	At5g30510	**At RPS1 L**	CCTGGTGACACTTTGAAGGTT	**60°C**	#78
			**At RPS1 R**	TGGGTTACGAATCATATCACCAG		
Elongation factor Tu, chloroplastic	*RABE1b*	At4g20360	**At RABE1b L**	TGCTATCAGAGAAGGAGGAAAGA	**60°C**	#67

			**At RABE1b R**	AACCTTATAATCATTCGAGGATCG		
Chloroplast stem-loop binding protein 41 kDa	*CSP41A*	At3g63140	**At CSP41A L**	GCCAAGAAGGCTTTTCTATTCA	**60°C**	#21
			**At CSP41A R**	TCCCACCCTAATAAGTCCTTAGC		
Oxygen-evolving enhancer protein 1-1, chloroplastic	*PSBO-1*	At5g66570	**At PSBO-1 L**	TCAAGGCAGACAGTGTAAGCA	**58°C**	#25
			**At PSBO-1 R**	AAATTGACGCTTCCGTCTGA		
Oxygen-evolving enhancer protein 1-2, chloroplastic	*PSBO-2*	At3g50820	**At PSBO-2 L**	CACTCGATGAGATCGAAGGAC	**60°C**	#52
			**At PSBO-2 R**	TGCTTAACCGTGAAGAGGAAC		
Chlorophyll a-b binding protein 2, chloroplastic	*CAB2*	At1g29920	**At CAB2 L**	CCAGGAACGGAGTCAAGTTT	**58°C**	#34
			**At CAB2 R**	TCCATCGCTGAAGATCTGTG		
Chlorophyll a-b binding protein 3, chloroplastic	*CAB3*	At1g29910	**At CAB3 L**	AGGCCGAGGACTTGCTTTA	**58°C**	#87
			**At CAB3 R**	CTCTGGGTCGGTAGCAAGAC		
Probable fructose-bisphosphate aldolase 1, chloroplastic	*FBA1*	At2g21330	**At FBA1 L**	CGCTACGCAGCTATTTCTCA	**60°C**	#36
			**At FBA1 R**	CGTGTTCTCCGTCCAACATA		
Fructose bisphosphate aldolase-like protein	*FBAlp*	At3g52930	**At FBAlp L**	CTCGATCTCTCCGTCCAACT	**58°C**	#8
			**At FBAlp R**	CAGCGTTGGCGATCAACT		
Cell division protease ftsH homolog 2, chloroplastic (VARIEGATED 2)	*VAR2*	At2g30950	**At VAR2 L**	CATCCTCCATATTTGCAGAGC	**58°C**	#12

			**At VAR2 R**	ACAAGACAAGCTGATGAAGCTG		
Phosphoribulokinase, chloroplastic	*PRK*	At1g32060	**At PRK L**	CATGTCACTGGACTTCTTGACC	**60°C**	#75
			**At PRK R**	AGTCTCTTACTCGCTCATCAAACAT		
RuBisCO large subunit-binding protein subunit alpha, chloroplastic	*CPN60A*	At2g28000	**At CPN60A L**	ATGGAAAATGCTGGTGCAG	**58°C**	#9
			**At CPN60A R**	GCAAGGATAGACGCAGTGGT		
Phosphoglycerate kinase, chloroplastic	*PGKchl*	At1g56190	**At PGKchl L**	CATCAACGATAAGACAAAATCCAT	**60°C**	#52
			**At PGKchl R**	GAGGGAATGTGTCCAAAGGA		
Glucan endo-1,3-beta-glucosidase, acidic isoform	*BGL2*	At3g57260	**At BGL2 L**	CAGCGAATGCGGCTCTAC	**60°C**	#21
			**At BGL2 R**	CTGGGAACGTCGAGGATG		
Protein disulfide-isomerase 2	*ATPDIL1-2*	At1g77510	**At ATPDIL1-2 L**	GTCCTGCTTCTGTTGAAATTAAGTC	**58°C**	#59
			**At ATPDIL1-2 R**	GATAATTTAGGGAACACTCCAACAG		
Probable protein disulfide-isomerase 1	*ATPDIL1-1*	At1g21750	**At ATPDIL1-1 L**	TGACAAGAAGGTTGTTGTGGTT	**60°C**	#36
			**At ATPDIL1-1 R**	CAATGGCCATGAAAGAATCA		
V-type proton ATPase subunit B1, vacuolar	*vATPS B1*	At1g76030	**At vATPS B1 L**	CCCTCTCTCTCTGCTCGAATC	**60°C**	#17
			**At vATPS B1 R**	CCCATTTTCACAGATCAACGA		
V-type proton ATPase catalytic subunit A	*VHA-A*	At1g78900	**At VHA-A L**	CGTTCTTCGAACACACAAGC	**60°C**	#72
			**At VHA-A R**	TCTGAATTCCATCAAAGATATTTCC		

14-3-3-like protein GF14 chi	*GRF1*	At4g09000	**At GRF1 L**	TAAACAACAATGGCGACACC	**60°C**	#73
			**At GRF1 R**	CTTGTTCGGCGAGTTTCG		
14-3-3-like protein GF14 omega	*GRF2*	At1g78300	**At GRF2 L**	ACAATCTCACTCTCTGGACATCTG	**60°C**	#38
			**At GRF2 R**	CTGTTCCTCGGTCGGTTTT		
14-3-3-like protein GF14 phi	*GRF4*	At1g35160	**At GRF4 L**	TTACTCTCTGGACTTCTGACATGC	**60°C**	#40
			**At GRF4 R**	AGAAACCACAAACTTAGATCTCCTTC		
14-3-3-like protein GF14 nu	*GRF7*	At3g02520	**At GRF7 L**	ACGTCATTGGTGCTAGGAGAG	**60°C**	#65
			**At GRF7 R**	CGTTTCCTCTGCTTTCTTCC		
14-3-3-like protein GF14 mu	*GRF9*	At2g42590	**At GRF9 L**	TGACCTTGTGGACTTCTGACAT	**60°C**	#22
			**At GRF9 R**	TGCACACATATCATATCACTCTGC		
14-3-3-like protein GF14 epsilon	*GRF10*	At1g22300	**At GRF10 L**	TCTTGAACTCTCCCGAAAGC	**58°C**	#90
			**At GRF10 R**	GAGTGCTGTCTTTGTATGATTCCTC		
Fe-superoxide dismutase 1	*FSD1*	At4g25100	**At FSD1 L**	AGTTCATGTTCGAGTTTCTCTGC	**58°C**	#46
			**At FSD1 R**	AGGACGTAGTTTGCGGTGAC		
Glycine-rich RNA-binding protein 7	*ATGRP7*	At2g21660	**ATGRP7 L**	ATGGGCCACTGATGACAGA	**60°C**	#8
			**ATGRP7 R**	CCTTGATCTTCCAGTCTCACG		
Elongation factor G, chloroplastic, SNOWY COTYLEDON 1	*ATSCO1*	At1g62750	**ATSCO1 L**	AACCCGGTGGTCTCAAGG	**60°C**	#53

			**ATSCO1 R**	TCCCTCTCAATGTACTCACGTACT		
Polyubiquitin 10	*UBQ10*	At4g05320	**UBQ10f**	AACGGGAAA GACGATTAC	**60^{0} C**	**-**
			**UBQ10r**	ACA AGA TGA AGG GTG GAC		

Table S2. The content of cytokinin metabolites and abscicis acid.

The active cytokinins include *trans*-zeatin (tZ), *trans*-zeatin riboside (tZR), isopentenyladenine (iP), isopentenyladenosine (iPR), dihydrozeatin (DZ), dihydrozeatin riboside (DZR), *cis*-zeatin (cZ); cytokinin O-glucosides include tZOG, tZROG, DZOG; cytokinin phosphates include tZRMP, iPRMP and DZRMP; *cis*-zeatin derivatives include cZOG and cZROG; cytokinin N-glucosides include tZ7G, tZ9G, iP7G, iP9G, DZ7G. S30 – 30-min HS applied to shoots; S180 - 180-min HS applied to shoots; SR30 - 30-min HS applied to shoots and roots; SR180 - 180-min HS applied to shoots and roots; R30 - 30-min HS applied to roots; R180 - 180-min HS applied to roots HS.

	Tissue	Active CKs						
Treatment	Leaves	*tZ*	*tZR*	*DZ*	*DZR*	*iP*	*iPR*	*cZ*
Control	Col-0	2.62 ± 1.06	3.45 ± 1.18	0.72 ± 0.43	0.71 ± 0.35	0.14 ± 0.18	1.36 ± 0.34	# ± #
	ipt + DEX	28.16 ± 8.02	23.15 ± 2.11	3.53 ± 1.89	# ± #	0.17 ± 0.20	3.70 ± 1.43	# ± #
S30	Col-0	3.48 ± 0.32	2.26 ± 0.44	0.32 ± 0.23	0.50 ± 0.44	0.12 ± 0.08	1.39 ± 0.46	# ± #
	Ipt + DEX	24.61 ± 2.34	23.33 ± 1.90	1.85 ± 0.62	3.10 ± 0.75	0.28 ± 0.20	2.31 ± 0.36	# ± #
S180	Col-0	1.54 ± 0.60	1.03 ± 0.08	0.27 ± 0.38	0.33 ± 0.25	0.62 ± 0.34	1.27 ± 0.54	# ± #
	ipt + DEX	19.53 ± 3.21	18.36 ± 1.54	3.13 ± 0.10	0.66 ± 0.02	0.03 ± 0.03	2.73 ± 0.19	0.03 ± 0.02
SR30	Col-0	2.38 ± 0.17	3.85 ± 0.24	0.57 ± 0.08	0.54 ± 0.03	# ± #	0.45 ± 0.03	# ± #
	ipt + DEX	22.11 ± 0.59	32.70 ± 0.87	0.73 ± 0.02	0.47 ± 0.01	0.10 ± 0.00	1.18 ± 0.03	0.13 ± 0.00
SR180	Col-0	1.55 ± 0.06	1.70 ± 0.16	0.43 ± 0.06	0.49 ± 0.06	0.05 ± 0.01	0.61 ± 0.05	0.08 ± 0.01
	ipt + DEX	4.44 ± 0.49	6.38 ± 0.70	1.41 ± 0.16	0.65 ± 0.07	# ± #	0.33 ± 0.04	0.17 ± 0.02
R30	Col-0	4.46 ± 0.35	1.98 ± 0.33	1.19 ± 0.10	0.32 ± 0.32	0.20 ± 0.06	0.36 ± 0.25	0.23 ± 0.04
	ipt + DEX	24.54 ± 0.85	5.05 ± 0.76	5.10 ± 1.23	2.08 ± 2.95	1.45 ± 1.03	23.79 ± 1.31	# ± #
R180	Col-0	5.79 ± 0.74	1.09 ± 0.02	0.24 ± 0.35	0.27 ± 0.38	0.17 ± 0.06	0.90 ± 0.10	0.04 ± 0.06
	ipt + DEX	24.51 ± 2.15	7.79 ± 0.73	3.97 ± 0.61	1.43 ± 0.35	0.04 ± 0.03	1.84 ± 0.27	# ± #

	Roots	*tZ*	*tZR*	*DZ*	*DZR*	*iP*	*iPR*	*cZ*
Control	Col-0	1.51 ± 0.56	2.50 ± 0.70	1.58 ± 0.34	0.61 ± 0.25	0.25 ± 0.08	0.32 ± 0.15	# ± #
	ipt + DEX	2.71 ± 0.95	2.92 ± 1.01	1.22 ± 0.45	0.68 ± 0.26	0.05 ± 0.06	1.34 ± 0.44	# ± #
S30	Col-0	1.86 ± 0.32	2.21 ± 0.14	1.49 ± 0.51	0.51 ± 0.46	0.98 ± 0.73	1.13 ± 0.22	# ± #

	ipt + DEX	5.06 ± 0.25	1.17 ± 0.11	1.70 ± 0.36	0.50 ± 0.42	0.09 ± 0.06	2.86 ± 0.34	0.01 ± 0.02
S180	Col-0	1.35 ± 0.05	1.06 ± 0.09	# ± #	1.59 ± 0.57	0.27 ± 0.15	1.64 ± 0.52	# ± #
	ipt + DEX	2.53 ± 0.64	1.76 ± 0.30	0.69 ± 0.31	1.00 ± 0.08	0.13 ± 0.03	1.02 ± 0.30	# ± #
SR30	Col-0	0.82 ± 0.07	0.78 ± 0.07	1.16 ± 0.10	2.01 ± 0.18	# ± #	0.12 ± 0.01	# ± #
	ipt + DEX	1.11 ± 0.09	0.85 ± 0.07	1.78 ± 0.15	1.34 ± 0.11	# ± #	0.23 ± 0.02	0.05 ± 0.00
SR180	Col-0	0.13 ± 0.09	1.16 ± 0.06	1.24 ± 0.03	1.20 ± 0.06	# ± #	0.26 ± 0.01	# ± #
	ipt + DEX	0.48 ± 0.17	1.47 ± 0.21	0.79 ± 0.14	0.44 ± 0.19	# ± #	0.12 ± 0.01	# ± #
R30	Col-0	1.32 ± 0.98	# ± #	1.07 ± 0.53	0.77 ± 0.38	0.29 ± 0.16	0.92 ± 0.59	# ± #
	ipt + DEX	2.57 ± 0.26	1.46 ± 0.25	0.72 ± 0.19	0.36 ± 0.22	0.03 ± 0.02	1.17 ± 0.13	0.01 ± 0.02
R180	Col-0	1.43 ± 1.17	# ± #	1.20 ± 0.30	1.73 ± 0.91	0.41 ± 0.30	0.39 ± 0.67	# ± #
	ipt + DEX	1.86 ± 0.12	0.68 ± 0.25	0.37 ± 0.27	0.19 ± 0.27	0.14 ± 0.10	0.75 ± 0.06	0.02 ± 0.00

	Tissue	**O-glc**			**P (nucleotides)**			**cisZ derivates**	
Treatment	Leaves	*DZOG*	*tZOG*	*tZROG*	*tZRMP*	*iPRMP*	*DZRMP*	*cZOG*	*cZROG*
Control	Col-0	# ± #	15.11 ± 2.58	2.25 ± 0.69	15.55 ± 2.24	15.79 ± 2.90	2.38 ± 1.35	1.05 ± 0.25	3.83 ± 1.27
	ipt + DEX	# ± #	63.78 ± 7.71	7.49 ± 2.23	160.49 ± 17.21	45.40 ± 7.02	7.99 ± 1.93	1.79 ± 0.47	3.28 ± 0.54
S30	Col-0	0.14 ± 0.10	15.79 ± 0.77	3.44 ± 0.39	15.49 ± 3.06	21.65 ± 2.54	2.35 ± 0.95	0.32 ± 0.05	3.21 ± 0.93
	ipt + DEX	# ± #	43.72 ± 2.83	4.64 ± 0.74	191.39 ± 12.22	20.83 ± 6.84	16.46 ± 2.25	3.70 ± 0.92	4.88 ± 0.37
S180	Col-0	0.19 ± 0.26	16.14 ± 3.66	3.94 ± 1.15	10.86 ± 0.61	0.82 ± 0.63	# ± #	1.45 ± 0.37	2.09 ± 0.58
	ipt + DEX	# ± #	23.09 ± 4.88	4.13 ± 0.88	96.80 ± 3.10	4.19 ± 0.13	4.03 ± 0.13	1.59 ± 0.61	6.93 ± 1.35
SR30	Col-0	# ± #	23.57 ± 0.84	2.60 ± 0.16	16.26 ± 1.43	6.89 ± 0.43	2.92 ± 0.18	2.11 ± 0.13	3.82 ± 0.24
	ipt + DEX	# ± #	35.94 ± 4.12	3.46 ± 0.09	180.01 ± 4.76	18.21 ± 0.48	7.79 ± 0.21	2.10 ± 0.06	3.39 ± 0.09
SR180	Col-0	# ± #	20.32 ± 0.77	1.84 ± 0.21	12.88 ± 0.80	9.70 ± 0.69	# ± #	1.55 ± 0.18	3.23 ± 0.37
	ipt + DEX	# ± #	29.85 ± 3.28	4.97 ± 0.55	36.67 ± 4.03	0.91 ± 0.10	4.69 ± 0.52	1.33 ± 0.15	7.34 ± 0.81
R30	Col-0	# ± #	22.89 ± 1.02	4.39 ± 1.17	5.46 ± 3.09	18.24 ± 0.61	1.67 ± 0.70	1.73 ± 0.17	3.16 ± 0.39
	ipt + DEX	# ± #	36.32 ± 3.54	7.65 ± 0.70	84.53 ± 6.21	38.70 ± 8.05	2.77 ± 0.76	5.18 ± 0.41	6.45 ± 1.17
R180	Col-0	# ± #	28.59 ± 2.46	2.04 ± 0.72	5.61 ± 2.39	17.06 ± 0.98	# ± #	1.68 ± 0.63	2.86 ± 0.37
	ipt + DEX	# ± #	33.06 ± 1.70	6.67 ± 2.85	121.56 ± 2.83	23.60 ± 5.66	3.84 ± 0.83	4.17 ± 1.08	4.26 ± 0.89

	Roots	*DZOG*	*tZOG*	*tZROG*	*tZRMP*	*iPRMP*	*DZRMP*	*cZOG*	*cZROG*

Control	Col-0	# ± #	0.70 ± 0.18	# ± #	5.69 ± 0.98	4.46 ± 0.83	# ± #	# ± #	0.10 ± 0.05
	ipt + DEX	# ± #	0.95 ± 0.46	0.32 ± 0.15	13.10 ± 1.66	3.47 ± 1.11	1.08 ± 0.60	# ± #	# ± #
S30	Col-0	0.18 ± 0.13	0.60 ± 0.30	0.42 ± 0.08	6.05 ± 2.02	6.76 ± 0.87	# ± #	# ± #	# ± #
	ipt + DEX	# ± #	0.53 ± 0.11	0.19 ± 0.02	12.14 ± 0.12	3.03 ± 0.64	0.47 ± 0.66	# ± #	0.02 ± 0.03
S180	Col-0	# ± #	1.11 ± 0.16	0.26 ± 0.22	4.90 ± 0.69	1.53 ± 0.24	# ± #	# ± #	0.16 ± 0.12
	ipt + DEX	# ± #	0.69 ± 0.13	0.20 ± 0.02	4.15 ± 0.10	1.79 ± 0.28	0.83 ± 0.13	# ± #	# ± #
SR30	Col-0	# ± #	0.74 ± 0.07	# ± #	3.99 ± 0.35	2.00 ± 0.18	1.39 ± 0.12	# ± #	0.22 ± 0.02
	ipt + DEX	# ± #	1.27 ± 0.15	0.54 ± 0.05	4.91 ± 0.41	1.33 ± 0.11	1.71 ± 0.14	# ± #	# ± #
SR180	Col-0	# ± #	1.34 ± 0.06	0.75 ± 0.04	2.24 ± 0.11	# ± #	# ± #	# ± #	0.02 ± 0.00
	ipt + DEX	# ± #	1.65 ± 0.10	0.54 ± 0.03	2.38 ± 0.15	# ± #	0.35 ± 0.02	# ± #	# ± #
R30	Col-0	# ± #	1.03 ± 0.70	# ± #	5.38 ± 2.95	1.39 ± 0.83	0.95 ± 0.47	# ± #	# ± #
	ipt + DEX	# ± #	0.42 ± 0.12	0.26 ± 0.05	10.61 ± 0.15	0.35 ± 0.07	0.39 ± 0.36	# ± #	0.07 ± 0.03
R180	Col-0	# ± #	0.91 ± 0.75	# ± #	1.22 ± 0.53	0.85 ± 0.47	1.06 ± 1.13	# ± #	# ± #
	ipt + DEX	# ± #	1.51 ± 0.20	0.31 ± 0.01	1.47 ± 0.75	5.27 ± 0.20	0.65 ± 0.62	# ± #	# ± #

		ABA	
		Leaves	Roots
DEX	Col-0	18.42 ± 0.11	3.26 ± 0.63
	ipt	18.64 ± 0.61	4.20 ± 0.14
S30 + DEX	Col-0	20.64 ± 1.23	2.53 ± 0.09
	ipt	28.57 ± 1.19	1.49 ± 0.16
S180 + DEX	Col-0	10.40 ± 0.83	1.57 ± 0.32
	ipt	22.30 ± 0.71	1.60 ± 0.16
SR30 + DEX	Col-0	28.66 ± 1.13	4.15 ± 0.41
	ipt	31.71 ± 1.31	2.83 ± 0.21
SR180 + DEX	Col-0	18.12 ± 1.79	3.22 ± 0.21
	ipt	22.55 ± 1.78	2.86 ± 0.21
R30 + DEX	Col-0	20.57 ± 0.42	2.48 ± 0.37
	ipt	20.12 ± 1.41	1.26 ± 0.13
R180 + DEX	Col-0	15.82 ± 0.73	2.47 ± 0.45

ipt	14.30	±	0.71	1.85	±	0.36

Table S3. The effect of individual HS treatments on expression levels of genes involved in cytokinin and abscisic acid metabolism and signalling, photosynthesis- and selected stress-related genes.

S30 – 30-min HS applied to shoots; S180 - 180-min HS applied to shoots; SR30 - 30-min HS applied to shoots and roots; SR180 - 180-min HS applied to shoots and roots; R30 - 30-min HS applied to roots; R180 - 180-min HS applied to roots HS.

CK-related genes

LEAVES	*ARR8*	*ARR9*	*ARR10*	*ARR12*	*AHK2*	*AHK3*	*AHK4*	*Agro IPT*
Col-0 Control	7.0919	3.6859	2.4712	1.3139	1.2348	1.0497	1.8051	ND
SD	0.0017	0.4871	0.0680	0.4586	0.1259	0.0268	0.0210	ND
Col-0 S30	0.1625	0.2161	0.5176	0.2966	0.2495	0.2308	0.2367	ND
SD	0.0580	0.1107	0.0510	0.0595	0.0073	0.0777	0.0718	ND
Col-0 S180	0.1000	0.0410	0.2572	0.1609	0.0726	0.0940	0.0660	ND
SD	0.0010	0.0007	0.0295	0.0056	0.0101	0.0138	0.0059	ND
ipt + DEX Control	4.7446	2.8308	1.9361	1.2667	0.9745	0.7537	1.6392	1.0380
SD	0.9224	0.0218	0.4257	0.1338	0.1187	0.1124	0.2673	0.3031
ipt S30	0.0689	0.0612	0.3817	0.2329	0.1638	0.1204	0.1769	0.5756
SD	0.0185	0.0109	0.1427	0.0596	0.0582	0.0274	0.0640	0.1444
ipt S180	0.0617	0.0376	0.2387	0.1358	0.0569	0.0842	0.0459	0.8575
SD	0.0175	0.0118	0.0487	0.0133	0.0068	0.0157	0.0039	0.2359
Col-0 SR30	0.1813	0.1286	0.2393	0.1861	0.1549	0.3351	0.1843	ND
SD	0.0275	0.0095	0.0413	0.0106	0.0006	0.0337	0.0339	ND
Col-0 SR30	0.5890	0.5882	0.5985	0.4710	0.9159	1.4946	0.3844	ND
SD	0.0163	0.0197	0.0194	0.0775	0.0809	0.0753	0.0125	ND
ipt SR30	0.1412	0.0943	0.1974	0.1832	0.1146	0.2763	0.2002	0.4860
SD	0.0001	0.0263	0.0358	0.0340	0.0347	0.0136	0.0754	0.0531
ipt SR180	0.4567	0.4074	0.5947	0.4206	0.7797	1.0593	0.2684	0.7967
SD	0.0375	0.2073	0.1356	0.0864	0.2384	0.0232	0.0005	0.1346
Col-0 R30	1.3232	1.4182	1.4820	0.8113	1.7257	1.5663	1.1673	ND

ABA-related genes

LEAVES	*AAO2*	*ABA1*	*NCED3*	*ABF2*	*ABI1*	*ABI5*	*ABI8*	*ATHB6*	*CYP707A3*	*CYP707A3*	*UGT75B1*	*UGT71B6*
Col-0 Control	2.9554	2.7213	0.3487	1.5163	1.3367	0.2441	1.4111	0.8903	5.1469	5.1469	0.5448	0.3568
SD	0.0813	0.1476	0.0506	0.0243	0.2645	0.1706	0.0285	0.0281	0.0829	0.0829	0.1332	0.0205
Col-0 S30	0.6457	0.6448	0.2343	0.3576	0.3153	0.0254	0.1268	0.1334	5.9922	5.9922	0.1799	0.1774
SD	0.0580	0.0675	0.0931	0.0621	0.0830	0.0032	0.0040	0.0157	1.7767	1.7767	0.0428	0.0736
Col-0 S180	0.4336	0.5888	0.0364	0.1067	0.1206	ND	0.2673	0.0259	1.2850	1.2850	0.2873	0.0972
SD	0.0284	0.0112	0.0205	0.0168	0.0045	ND	0.0695	0.0058	0.1067	0.1067	0.0330	0.0077
ipt + DEX Control	1.9026	2.1964	0.2612	1.1230	1.5862	0.5137	1.2972	0.7980	3.7949	3.7949	0.7595	0.1609
SD	0.3822	0.0063	0.0914	0.0323	0.1578	0.1073	0.2902	0.0358	0.6091	0.6091	0.0797	0.0777
ipt S30	0.4130	0.5418	0.6249	0.3470	0.1651	0.0435	0.0804	0.0831	9.8336	9.8336	0.2222	0.1152
SD	0.1473	0.1899	0.1011	0.0824	0.0258	0.0144	0.0199	0.0284	3.5366	3.5366	0.0584	0.0100
ipt S180	0.3186	0.4881	0.0205	0.0801	0.0687	0.0410	0.3540	0.0161	1.3506	1.3506	0.2395	0.0740
SD	0.0790	0.1291	0.0043	0.0061	0.0108	0.0026	0.0180	0.0235	0.5415	0.5415	0.0020	0.0141
Col-0 SR30	0.2627	0.4300	1.3887	0.3956	0.2166	0.0331	0.2830	0.2940	1.0803	0.4130	2.0153	0.3176
SD	0.0620	0.0000	0.7807	0.1535	0.0521	0.0139	0.0469	0.0198	0.5266	0.0050	0.4557	0.0259
Col-0 SR30	0.3690	0.9632	1.7115	0.5764	1.1297	0.4264	1.0674	1.1561	0.4130	1.1772	0.7739	0.6647
SD	0.0327	0.0000	0.1933	0.0013	0.1362	0.0828	0.0538	0.0194	0.0050	0.2023	0.1749	0.2140
ipt SR30	0.2344	0.3156	1.5202	0.2309	0.3172	0.0558	0.2687	0.2817	0.8158	0.8158	0.1759	0.1870
SD	0.0861	0.0000	0.4714	0.0487	0.0649	0.0188	0.0410	0.0567	0.1349	0.1349	0.0219	0.0388
ipt SR180	0.2759	1.0584	1.8642	0.7887	1.1191	0.4521	1.0574	1.4086	0.3825	0.3825	1.8976	0.3075
SD	0.0740	0.0000	1.0055	0.2137	0.5460	0.3372	0.0524	0.3725	0.1383	0.1383	1.0097	0.0288
Col-0 R30	1.3386	2.4471	37.6466	3.5283	3.5978	1.1511	2.2428	1.5171	0.6887	0.6887	1.6862	1.7838

Photosynthesis-related genes

LEAVES	*CAB2*	*CAB3*	*CPN60A*	*CSP41A*	*FBA1*	*FBAIp*	*FSD1*	*PSBO1*	*PSBO2*	*RPS1*	*SCO1*	*VAR2*	*ACD2*	*ATB2*
Col-0 Control	2.2224	1.5248	1.6044	1.8097	1.5272	1.9111	1.5043	1.5170	1.4147	1.3473	2.0459	1.7526	0.8810	0.6466
SD	0.0954	0.1335	0.0045	0.0724	0.1185	0.1115	0.3542	0.1145	0.1236	0.0607	0.1719	0.1366	0.2876	0.1337
Col-0 S30	0.7444	0.5158	1.9605	0.5847	0.7571	1.6188	0.6909	0.5305	0.8181	0.4806	0.4960	0.6008	0.3376	0.4493
SD	0.1119	0.1713	0.2535	0.0873	0.2295	1.0091	0.1228	0.1192	0.2091	0.0032	0.0208	0.0340	0.0510	0.0222
Col-0 S180	0.7247	0.4063	1.7631	0.5048	0.4547	1.1896	0.5634	0.3814	0.5100	0.4597	0.4607	0.5947	0.2403	0.6341
SD	0.1855	0.0915	0.1508	0.0671	0.0506	0.2584	0.0305	0.0112	0.0108	0.0554	0.0924	0.0478	0.0363	0.0300
ipt + DEX Control	2.2289	1.3969	1.2768	1.9713	1.0517	1.6954	1.9799	1.5046	1.4834	1.4864	1.8055	1.5973	0.9571	1.0371
SD	0.5940	0.2030	0.5408	0.6361	0.2120	0.5353	0.3936	0.2139	0.1871	0.1650	0.2471	0.4328	0.0953	0.1581
ipt S30	0.7342	0.4771	2.2883	0.5836	0.3717	1.4582	0.6104	0.4804	0.5925	0.5141	0.4565	0.7115	0.4017	0.5269
SD	0.0914	0.0577	0.2239	0.0157	0.0197	0.1346	0.0464	0.1255	0.1244	0.1089	0.0791	0.1967	0.1388	0.0545
ipt S180	0.6000	0.3259	1.4784	0.5073	0.4367	0.9414	0.5245	0.3747	0.5147	0.4846	0.4976	0.6400	0.2220	0.4282
SD	0.1844	0.0585	0.0122	0.0577	0.1834	0.0732	0.1273	0.0110	0.0469	0.0632	0.0347	0.0125	0.0217	0.1382
Col-0 SR30	0.4871	0.4398	2.0550	0.2944	0.6281	1.7468	0.2542	0.4397	0.5050	0.4872	0.3524	0.5350	0.4142	0.3448
SD	0.1276	0.1258	0.0432	0.2693	0.0442	0.0680	0.0834	0.0680	0.0835	0.0535	0.1189	0.0787	0.0578	0.0006
Col-0 SR30	1.4274	0.9223	2.1548	0.8900	0.1974	0.5058	0.8492	0.9585	1.0735	0.8218	0.6157	1.4669	0.9162	1.2817
SD	0.0223	0.0315	0.2664	0.0368	0.0687	0.1002	0.1329	0.1363	0.0860	0.0810	0.0646	0.2845	0.0591	0.0728
ipt SR30	0.4249	0.5229	2.1849	0.3840	0.4054	2.0268	0.4383	0.3989	0.4750	0.4978	0.3731	0.4773	0.3617	0.3518
SD	0.1941	0.0183	0.4496	0.1461	0.0289	0.7144	0.0555	0.1298	0.1850	0.2193	0.1109	0.1074	0.0464	0.0846
ipt SR180	1.2583	0.7764	2.4892	0.7103	0.1351	0.5077	0.7668	0.8611	0.9964	0.7853	0.6918	1.3982	0.7788	1.2654
SD	0.3363	0.2044	0.2260	0.2893	0.0953	0.1319	0.0444	0.3007	0.2404	0.1575	0.1958	0.5414	0.1655	0.0707
Col-0 R30	1.0199	1.0440	1.2794	1.0462	0.6622	1.7143	1.3009	1.1340	1.0189	1.2297	1.4134	1.1992	2.3323	1.7766

Others

LEAVES	*ATPDIL1-1*	*ATPDIL1-2*	*GRF1*	*GRF2*	*GRF4*	*GRF7*	*GRF9*	*GRF10*	*GRP7*	*MDcyt1*	*RABE1b*	*VI*[illegible]
Col-0 Control	0.4767	0.3329	1.1936	1.7423	1.3676	1.0352	1.2949	1.2774	0.2324	0.9673	1.6218	[illegible]
SD	0.0033	0.0091	0.0525	0.0466	0.4907	0.0838	0.0579	0.1715	0.0065	0.0214	0.0099	[illegible]
Col-0 S30	0.0920	0.0643	0.3869	0.5455	0.4611	0.3903	0.4973	0.4379	0.1061	0.2692	0.4064	[illegible]
SD	0.0235	0.0121	0.0784	0.0706	0.0707	0.0259	0.0788	0.0344	0.1040	0.0185	0.0339	[illegible]
Col-0 S180	0.0488	0.1177	0.2183	0.5616	0.2958	0.3017	0.4506	0.2597	0.0690	0.1683	0.4940	[illegible]
SD	0.0100	0.0327	0.0196	0.1302	0.0443	0.0152	0.0744	0.0038	0.0115	0.0035	0.0087	[illegible]
ipt + DEX Control	0.9978	1.2905	1.1166	1.6479	1.3639	1.0790	1.1241	1.1501	0.1543	0.9454	1.4408	[illegible]
SD	0.4314	0.0511	0.2872	0.3085	0.4516	0.3259	0.0367	0.2471	0.0284	0.0937	0.2517	[illegible]
ipt S30	0.0960	0.0845	0.3517	0.4741	0.3883	0.3349	0.4484	0.3897	0.1436	0.2923	0.4947	[illegible]
SD	0.0325	0.0322	0.0325	0.0113	0.0324	0.0877	0.0993	0.0940	0.0160	0.0683	0.0980	[illegible]
ipt S180	0.0488	0.1439	0.2380	0.5762	0.3627	0.3132	0.3892	0.3186	0.0764	0.1984	0.4993	[illegible]
SD	0.0106	0.0434	0.0168	0.0536	0.0338	0.0431	0.0065	0.0314	0.0021	0.0169	0.0247	[illegible]
Col-0 SR30	0.1486	0.1538	0.2859	0.3406	0.3107	0.3166	0.5703	0.3120	0.1440	0.2665	0.4606	[illegible]
SD	0.0311	0.0598	0.0437	0.0506	0.0499	0.0024	0.1255	0.0177	0.0334	0.0398	0.0186	[illegible]
Col-0 SR30	0.3155	0.5866	0.8418	0.4411	0.5531	0.7648	1.9898	0.6700	0.7682	0.4705	0.5442	[illegible]
SD	0.0073	0.0263	0.1806	0.0930	0.0270	0.0366	0.0135	0.1357	0.1758	0.0284	0.0757	[illegible]
ipt SR30	0.2062	0.3626	0.3506	0.3686	0.3233	0.3892	0.6182	0.3257	0.1919	0.2772	0.4038	[illegible]
SD	0.0122	0.0471	0.0692	0.0986	0.1326	0.0443	0.0720	0.0923	0.0248	0.0811	0.1643	[illegible]
ipt SR180	0.4585	0.8246	0.8118	0.3851	0.5943	0.8712	1.6423	0.6610	0.7329	0.4618	0.5182	[illegible]
SD	0.1636	0.2549	0.0117	0.0433	0.0630	0.1502	0.1283	0.1041	0.1124	0.0034	0.1251	[illegible]
Col-0 R30	1.1598	1.0765	1.2542	1.3892	1.0563	1.3760	1.2337	1.0702	0.3262	1.1909	1.1722	[illegible]

SD	0.2401	0.4059	0.3788	0.1770	0.3223	0.1739	0.1979	ND
Col-0 R180	1.1475	1.6436	1.1853	1.5261	2.8810	1.5939	0.8342	ND
SD	0.4606	0.2044	0.0446	0.3766	0.5416	0.0096	0.0363	ND
ipt SR30	0.8796	1.0705	0.9870	0.7991	1.1933	1.0311	0.8137	0.7200
SD	0.0315	0.3349	0.1055	0.0215	0.0172	0.0313	0.2775	0.1275
ipt SR180	1.6786	1.8731	0.9639	1.0549	1.5590	1.1537	0.9821	0.7617
SD	0.6559	0.6467	0.2858	0.1892	0.4813	0.1855	0.3947	0.3838
ROOTS	*ARR8*	*ARR9*	*ARR10*	*ARR12*	*AHK2*	*AHK3*	*AHK4*	*Agro IPT*
Col-0 Control	7.6283	1.6197	4.9264	3.1865	2.4663	1.2446	8.0744	ND
SD	0.8890	0.4051	0.7022	0.4520	0.9576	0.1993	0.9485	ND
Col-0 S30	4.5236	1.6820	4.7188	3.8851	2.8944	1.3868	7.6341	ND
SD	1.1934	0.7760	1.1998	0.7539	0.8340	0.2836	0.9634	ND
Col-0 S180	2.7091	0.3843	2.0675	1.5937	1.2280	1.6543	4.6277	ND
SD	0.7146	0.1329	0.6327	0.3163	0.2267	0.3715	0.9644	ND
ipt + DEX Control	3.7127	2.5535	5.8987	2.4506	1.8000	1.2530	7.3545	1.1130
SD	0.8364	0.8506	3.5507	0.8794	0.6338	0.3713	1.4780	0.0584
ipt S30	5.1641	1.4741	6.8464	3.4861	2.2279	1.2207	5.7467	1.5410
SD	1.0778	0.6250	2.0262	0.4797	0.3782	0.3824	0.8252	0.5779
ipt S180	2.8229	0.5228	2.9386	2.0169	1.3358	1.7366	6.7102	1.4902
SD	0.5401	0.1281	0.5766	0.2037	0.2678	0.1397	0.8566	0.7678
Col-0 SR30	2.3573	1.1269	2.5141	1.7116	1.0141	0.3539	1.7219	ND
SD	0.6183	0.1498	0.5509	0.2177	0.1236	0.0398	0.0597	ND
Col-0 SR30	2.0716	1.3453	1.9635	1.4046	0.8288	0.4070	1.2298	ND
SD	0.3350	0.3280	0.6243	0.3406	0.1687	0.0701	0.0839	ND
ipt SR30	2.7080	1.3033	3.5189	2.0385	1.0596	0.4514	2.3646	0.7243
SD	0.0343	0.4248	0.2352	0.5666	0.4186	0.1614	0.8996	0.1205
ipt SR180	2.3297	1.3919	1.9277	1.3817	0.8587	0.3589	1.1462	0.6126

SD	0.3129	0.7016	15.4845	0.7947	1.4414	0.7055	0.0527	0.4604	0.0000	0.1260	0.3492	0.1805
Col-0 R180	1.0664	2.3112	6.2277	1.7374	1.7226	1.5549	1.9175	1.9848	3.7365	3.7365	3.0104	0.9417
SD	0.1313	0.2220	1.4556	0.1826	0.0418	0.6933	0.0018	0.0720	0.6331	0.6331	0.1364	0.0427
ipt SR30	0.6529	1.5212	1.7885	1.0671	1.3880	0.4953	1.4733	1.4685	5.3708	5.3708	1.1437	1.1086
SD	0.2192	0.2599	0.0377	0.3287	0.0792	0.0914	0.0891	0.0594	1.2569	1.2569	0.4076	0.3633
ipt SR180	0.7246	1.2044	2.5227	1.0903	1.3098	0.7565	1.1648	1.3560	3.1237	3.1237	1.5872	0.7924
SD	0.1360	0.5481	0.5311	0.3097	0.3823	0.5077	0.2685	0.5351	0.9770	0.9770	0.6203	0.0525
ROOTS	*AAO2*	*ABA1*	*NCED3*	*ABF2*	*ABI1*	*ABI5*	*ABI8*	*ATHB6*	*CYP707A3*	*CYP707A3*	*UGT75B1*	*UGT71B6*
Col-0 Control	2.7948	0.1021	0.0780	1.4611	0.7055	0.3210	2.0313	0.2914	29.2510	19.9864	0.0038	0.1198
SD	0.5810	0.0207	0.0115	0.2920	0.1336	0.1090	0.4120	0.0872	4.3641	3.5072	0.0031	0.0222
Col-0 S30	3.5779	0.1041	0.0720	1.2873	0.8852	0.5374	2.7461	0.3845	18.3947	18.3947	0.0059	0.2007
SD	1.1227	0.0184	0.0235	0.3210	0.1654	0.0508	0.9149	0.0393	3.6541	3.6541	0.0034	0.0146
Col-0 S180	1.1987	0.0980	0.1976	0.4748	0.4882	0.1941	1.2193	0.2679	22.8591	22.8591	0.0234	0.1092
SD	0.3117	0.0232	0.0573	0.1705	0.1089	0.0836	0.2938	0.0478	2.9037	2.9037	0.0131	0.0291
ipt + DEX Control	3.1592	0.1285	0.0834	1.8163	0.5745	0.5820	2.6409	0.3383	42.7255	18.5187	0.0089	0.0847
SD	1.1976	0.0294	0.0225	0.5559	0.0884	0.3133	1.2341	0.0953	4.8906	4.4849	0.0096	0.0129
ipt S30	2.6039	0.0925	0.0440	1.2458	0.8720	0.4981	2.2429	0.3578	22.0813	22.0813	0.0032	0.1778
SD	0.4197	0.0073	0.0060	0.3337	0.0867	0.1646	0.5876	0.0612	3.6584	3.6584	0.0016	0.0478
ipt S180	1.2660	0.1114	0.1435	0.5360	0.5044	0.1179	1.2075	0.2700	32.2148	32.2148	0.0053	0.1008
SD	0.2184	0.0223	0.0299	0.1109	0.1119	0.0313	0.2274	0.0448	4.3674	4.3674	0.0031	0.0146
Col-0 SR30	1.4821	0.0490	0.2657	0.8014	0.2848	0.2518	0.5307	0.1413	17.2136	17.2136	0.0187	0.0971
SD	0.1548	0.0023	0.1039	0.0631	0.1083	0.0745	0.1003	0.0108	2.3277	2.3277	0.0061	0.0251
Col-0 SR30	0.9534	0.0476	0.0599	1.0068	0.2510	0.1259	0.3984	0.1463	12.1313	12.1313	0.0218	0.1224
SD	0.2536	0.0272	0.0137	0.3084	0.0700	0.0537	0.2007	0.0185	2.3454	2.3454	0.0091	0.0454
ipt SR30	1.6611	0.0555	0.2285	0.9310	0.4552	0.2132	0.8799	0.1318	14.6472	14.6472	0.0291	0.0668
SD	0.7103	0.0186	0.0469	0.5225	0.1945	0.0890	0.2518	0.0475	0.7547	0.7547	0.0189	0.0180
ipt SR180	0.8415	0.0490	0.0808	0.9804	0.2484	0.1409	0.3081	0.1271	10.3833	10.3833	0.0230	0.0901

SD	0.0671	0.0303	0.2110	0.0994	0.0466	0.2576	0.1392	0.0761	0.0734	0.1332	0.1135	0.0962	0.4344	0.1180
Col-0 R180	1.2242	1.2656	0.9314	0.9838	0.9655	1.3432	0.7197	1.0094	0.9875	1.3073	1.0546	0.9710	1.6843	1.4145
SD	0.2164	0.3308	0.0969	0.0164	0.1553	0.1370	0.0855	0.1183	0.1289	0.2783	0.1780	0.1847	0.1337	0.0419
ipt SR30	0.8720	0.6808	0.7341	0.7791	0.1707	2.0852	0.8238	0.6655	0.6693	0.7151	0.8172	0.7412	0.9805	1.3715
SD	0.1572	0.1572	0.0434	0.1235	0.2394	0.7064	0.2040	0.0613	0.0195	0.0798	0.2294	0.1228	0.0947	0.0715
ipt SR180	0.7593	0.7281	0.7366	0.7857	0.6642	1.0711	0.8548	1.0086	0.9067	0.9619	0.7763	0.8169	1.0158	1.1743
SD	0.1425	0.0617	0.1567	0.1986	0.8919	0.1966	0.2492	0.1629	0.3262	0.2592	0.1821	0.1431	0.3158	0.1543
ROOTS	*CAB2*	*CAB3*	*CPN60A*	*CSP41A*	*FBA1*	*FBAIp*	*FSD1*	*PSBO1*	*PSBO2*	*RPS1*	*SCO1*	*VAR2*	*ACD2*	*ATB2*
Col-0 Control	0.0004	0.0001	0.3643	0.0002	0.0004	4.3970	0.1483	0.0005	0.0005	0.0667	0.0207	0.0389	0.0743	3.3598
SD	0.0002	0.0001	0.1215	0.0001	0.0005	1.0441	0.0854	0.0001	0.0001	0.0320	0.0127	0.0127	0.0227	0.5232
Col-0 S30	0.0004	0.0001	0.5067	0.0003	0.0005	4.6446	0.0431	0.0007	0.0005	0.0616	0.0231	0.0488	0.0925	3.9358
SD	0.0002	0.0002	0.0734	0.0001	0.0005	0.6041	0.0251	0.0002	0.0002	0.0013	0.0064	0.0136	0.0247	0.3886
Col-0 S180	0.0007	0.0001	3.4435	0.0009	0.0011	1.6397	0.0414	0.0005	0.0003	0.0483	0.0114	0.0469	0.1036	2.0136
SD	0.0006	0.0003	0.6165	0.0011	0.0018	0.1343	0.0203	0.0003	0.0001	0.0197	0.0022	0.0090	0.0431	0.2096
ipt + DEX Control	0.0005	0.0003	0.5584	0.0007	0.0006	5.7653	0.0346	0.0020	0.0044	0.0632	0.0252	0.0437	0.0759	3.2731
SD	0.0004	0.0006	0.2642	0.0009	0.0009	1.5507	0.0009	0.0010	0.0058	0.0166	0.0122	0.0113	0.0165	0.7442
ipt S30	0.0002	0.0000	0.5743	0.0004	0.0003	5.3381	0.0466	0.0008	0.0007	0.0827	0.0282	0.0611	0.0728	3.6422
SD	0.0000	0.0001	0.1641	0.0001	0.0004	0.5676	0.0160	0.0002	0.0001	0.0175	0.0043	0.0094	0.0089	0.3133
ipt S180	0.0004	0.0001	2.3760	0.0005	0.0025	1.9759	1.9515	0.0003	0.0003	0.0614	0.0110	0.0497	0.1215	2.7521
SD	0.0001	0.0002	0.5632	0.0005	0.0041	0.0738	0.3903	0.0001	0.0002	0.0176	0.0020	0.0062	0.0303	0.0868
Col-0 SR30	0.0005	0.0002	0.4088	0.0011	0.0005	2.8956	0.0004	0.0005	0.0008	0.0484	0.0195	0.0337	0.1532	2.5228
SD	0.0008	0.0003	0.0601	0.0008	0.0004	0.3552	0.0001	0.0000	0.0002	0.0171	0.0014	0.0052	0.0587	0.3359
Col-0 SR30	0.0002	0.0001	0.2605	0.0006	0.0441	2.2012	0.0002	0.0008	0.0004	0.0546	0.0223	0.0391	0.2413	1.8380
SD	0.0003	0.0001	0.0630	0.0005	0.0763	0.2807	0.0000	0.0003	0.0001	0.0055	0.0025	0.0024	0.0778	0.4703
ipt SR30	0.0001	0.0001	0.4492	0.0007	0.0238	3.4005	0.0004	0.0015	0.0012	0.0646	0.0222	0.0408	0.1679	3.4185
SD	0.0002	0.0002	0.1672	0.0002	0.0474	1.0059	0.0002	0.0007	0.0003	0.0182	0.0089	0.0108	0.0456	0.9141
ipt SR180	0.0002	0.0001	0.1497	0.0004	0.0619	1.6871	0.0002	0.0012	0.0007	0.0452	0.0268	0.0429	0.1655	2.4285

SD	0.3349	0.3326	0.1352	0.0632	0.2966	0.1409	0.0196	0.0098	0.0080	0.1419	0.1594	[illegible]
Col-0 R180	0.6304	0.5667	0.9363	1.3350	0.8845	0.9470	1.1280	0.9208	0.5600	0.8592	0.9288	[illegible]
SD	0.0072	0.0067	0.0984	0.1787	0.0471	0.0738	0.0541	0.1579	0.1186	0.0625	0.0841	[illegible]
ipt SR30	1.9477	1.8182	1.0006	1.2291	0.7942	1.1814	0.8316	1.3185	0.3720	0.7561	0.7455	[illegible]
SD	0.0063	0.1943	0.2230	0.2221	0.1064	0.0133	0.1706	0.6197	0.0182	0.0987	0.1231	[illegible]
ipt SR180	0.7538	0.6421	0.7829	0.9399	0.7615	0.8588	0.8955	0.8181	0.6023	0.7017	0.7536	[illegible]
SD	0.1546	0.1290	0.1735	0.3007	0.1519	0.1047	0.1402	0.1887	0.0105	0.1196	0.2056	[illegible]
ROOTS	*ATPDIL1-1*	*ATPDIL1-2*	*GRF1*	*GRF2*	*GRF4*	*GRF7*	*GRF9*	*GRF10*	*GRP7*	*MDcyt1*	*RABE1b*	*V*[illegible]
Col-0 Control	1.4294	0.4578	1.6601	3.4794	4.3236	1.6601	0.4387	2.0400	1.3770	1.6395	0.0640	[illegible]
SD	0.4088	0.1678	0.2050	0.6375	0.5233	0.2050	0.1216	0.3923	0.1122	0.4108	0.0139	[illegible]
Col-0 S30	1.6048	0.4096	1.8152	4.0747	4.9876	1.8152	0.5524	2.1544	2.4025	1.6164	0.0736	[illegible]
SD	0.2178	0.0466	0.0844	0.5895	0.9029	0.0844	0.0689	0.1416	0.4177	0.2566	0.0014	[illegible]
Col-0 S180	0.5718	0.5375	0.8750	1.9762	2.1163	0.8077	0.3481	1.2293	1.0730	0.7614	0.0713	[illegible]
SD	0.1486	0.0388	0.1562	0.2366	0.3482	0.0970	0.0455	0.3237	0.5301	0.1016	0.0147	[illegible]
ipt + DEX Control	1.5962	0.4090	1.9853	4.1033	3.1315	1.6262	0.4138	2.0777	1.5172	1.4870	0.0532	[illegible]
SD	0.5778	0.0907	0.6133	1.7503	0.7498	0.3587	0.1208	0.7077	0.3191	0.3043	0.0092	[illegible]
ipt S30	2.1293	0.5036	2.1289	5.3316	3.4314	2.0670	0.5263	2.7492	2.0084	2.0548	0.0786	[illegible]
SD	0.2524	0.0748	0.3684	0.7448	0.7729	0.2831	0.0762	0.4433	0.6640	0.2483	0.0185	[illegible]
ipt S180	1.0349	0.5497	1.1450	2.8870	2.3302	1.0170	0.3502	1.3700	1.4740	0.9031	0.0694	[illegible]
SD	0.1197	0.1357	0.3403	0.7297	0.3494	0.2745	0.0690	0.5926	0.4423	0.2268	0.0144	[illegible]
Col-0 SR30	0.5830	0.1042	1.1545	2.5947	2.0461	1.0547	0.3121	1.4023	1.3191	1.0593	0.0411	[illegible]
SD	0.1194	0.0127	0.1132	0.2641	0.0576	0.1872	0.0439	0.0913	0.5656	0.0811	0.0102	[illegible]
Col-0 SR30	0.3138	0.0564	0.9233	1.5591	1.7913	1.1022	0.3694	1.4184	0.4978	1.1617	0.0489	[illegible]
SD	0.1055	0.0187	0.2130	0.3912	0.6373	0.1045	0.0686	0.3509	0.1419	0.2949	0.0049	[illegible]
ipt SR30	0.8856	0.1293	1.6800	3.2650	2.3243	1.7249	0.3901	1.8205	3.0176	1.3617	0.0483	[illegible]
SD	0.2748	0.0334	0.4622	1.0359	0.0234	0.5012	0.1202	0.3127	1.7009	0.4110	0.0144	[illegible]
ipt SR180	0.1979	0.0467	0.9032	1.3460	1.8430	0.8042	0.3495	1.2425	0.3357	0.8361	0.0462	[illegible]

SD	1.0094	0.4730	0.5509	0.4948	0.2821	0.1118	0.2954	0.1918
Col-0 R30	5.2084	1.5633	4.2577	2.4661	1.7868	0.4088	2.4471	ND
SD	1.9378	0.4042	1.6336	0.7962	0.3505	0.0605	0.4458	ND
Col-0 R180	3.1073	1.4572	4.2572	1.8139	1.7924	0.3820	1.6830	ND
SD	0.4772	0.5312	0.7410	0.1282	0.3127	0.0980	0.3507	ND
ipt SR30	2.7982	0.8623	3.6032	1.9612	1.5629	0.4123	2.1406	0.7852
SD	1.3847	0.2093	1.0851	0.5201	0.2361	0.0812	0.4130	0.1281
ipt SR180	1.9287	0.6929	2.2705	1.0133	0.7558	0.3140	1.0359	1.1175
SD	0.9852	0.1191	0.3447	0.0854	0.2082	0.0949	0.1863	0.3079

SD	0.0167	0.0165	0.0632	0.2840	0.0781	0.1010	0.1107	0.0223	0.2346	0.2346	0.0082	0.0115
Col-0 R30	2.7086	0.1019	0.4313	1.3881	0.5842	0.3820	1.2614	0.2389	53.2040	53.2040	0.0310	0.1660
SD	0.8086	0.0189	0.1707	0.4176	0.1428	0.0986	0.2725	0.0711	9.0585	9.0585	0.0085	0.0477
Col-0 R180	2.2737	0.0953	0.1787	1.9945	0.3323	0.2690	0.6379	0.1936	43.3290	43.3290	0.0200	0.1190
SD	0.1637	0.0145	0.0096	0.5747	0.0217	0.0875	0.1063	0.0444	8.4996	8.4996	0.0124	0.0149
ipt SR30	2.1156	0.0702	1.0217	1.4081	0.5080	0.3377	0.9718	0.1179	47.3582	47.3582	0.0192	0.1052
SD	0.4003	0.0077	0.2056	0.2301	0.1466	0.0561	0.1782	0.0408	5.5620	5.5620	0.0036	0.0364
ipt SR180	1.2184	0.0365	0.2086	1.6414	0.4818	0.2786	0.5274	0.1369	19.5687	19.5687	0.0044	0.1114
SD	0.4189	0.0151	0.0062	0.7475	0.3796	0.1487	0.1455	0.0806	6.4372	6.4372	0.0007	0.0299

SD	0.0004	0.0003	0.0086	0.0003	0.1231	0.2435	0.0001	0.0003	0.0003	0.0067	0.0089	0.0102	0.0501	0.5098
Col-0 R30	0.0006	0.0002	0.4166	0.0012	0.0002	2.7813	0.2150	0.0004	0.0008	0.0768	0.0210	0.0399	0.2285	4.2300
SD	0.0003	0.0005	0.0999	0.0004	0.0000	0.5608	0.1125	0.0002	0.0002	0.0223	0.0077	0.0067	0.0493	0.9733
Col-0 R180	0.0002	0.0001	0.3917	0.0016	0.0002	2.8020	0.1411	0.0008	0.0007	0.0780	0.0244	0.0529	0.1360	3.8937
SD	0.0002	0.0001	0.0550	0.0007	0.0000	0.3747	0.0082	0.0001	0.0002	0.0124	0.0034	0.0083	0.0373	0.4604
ipt SR30	0.0003	0.0001	0.9537	0.0006	0.0001	2.3061	0.0685	0.0005	0.0020	0.0604	0.0165	0.0319	0.1221	3.5365
SD	0.0004	0.0002	0.2141	0.0000	0.0002	0.2850	0.0243	0.0001	0.0004	0.0045	0.0020	0.0038	0.0505	0.4802
ipt SR180	0.0564	0.0005	0.4626	0.0030	0.0001	1.8583	0.0871	0.0003	0.0009	0.0552	0.0166	0.0368	0.0584	2.5383
SD	0.0949	0.0011	0.1362	0.0010	0.0000	0.5391	0.0181	0.0001	0.0003	0.0152	0.0026	0.0092	0.0180	0.1118

SD	0.0557	0.0110	0.2830	0.0724	0.4150	0.1068	0.0461	0.2633	0.1451	0.1617	0.0128	[illegible]
Col-0 R30	0.7916	0.1556	1.1453	2.8240	2.1154	1.3027	0.3503	2.0708	1.2841	1.0618	0.0573	[illegible]
SD	0.2074	0.0232	0.2094	0.5200	0.2951	0.3583	0.0735	0.2725	0.6713	0.1195	0.0147	[illegible]
Col-0 R180	0.4590	0.1018	1.1677	3.1071	2.2939	1.1509	0.3387	1.8960	0.4795	1.1678	0.0630	[illegible]
SD	0.1290	0.0306	0.2275	0.5874	0.3795	0.1975	0.0162	0.1927	0.0904	0.1707	0.0106	[illegible]
ipt SR30	0.5919	0.1601	1.0536	2.4226	1.9337	1.0173	0.2985	1.6837	0.8419	0.9699	0.0452	[illegible]
SD	0.0814	0.0456	0.1866	0.4557	0.1967	0.1280	0.0226	0.1951	0.1288	0.1216	0.0076	[illegible]
ipt SR180	0.3530	0.1365	0.7432	1.9978	1.3847	0.7432	0.2512	1.5763	0.4009	0.7694	0.0433	[illegible]
SD	0.1222	0.0269	0.1215	0.3314	0.7442	0.1215	0.0330	0.4673	0.1475	0.1704	0.0064	[illegible]

Table S4. Complete list of identified proteins regulated by individual HS treatments in wild-type (Col-0) and activated *ipt* transformant leaves. Molecular Weight (MW; theoretical, theo; experimental, exp.); Isoelectric point (pI; theoretical, theo; experimental, exp.); Relative spot volume (V%); Relative spot volume change.

Spot N.	AGI (TAIR)	Name (UniProt)	Score	MW	pI	Wild type (Col-0)							Plants with increased CK levels (11.5)						
							40° C heat only on shoots		40° C heat to shoot&roots		40° C heat only to roots			40° C heat only to shoots		40° C heat to shoots&roots		40° C heat only to roots	
				(theo./exp.)	(theo./exp.)	V%	30 min	180 min	30 min	180 min	30 min	180 min	V%	30 min	180 min	30 min	180 min	30 min	180 min
S01	At4g02520	Glutathione S-transferase PM24	138	24.1 / 21.5	5.9 / 6.5	0.34	1.08 ± 0.04	1.31 ± 0.16	0.89 ± 0.02	0.90 ± 0.04	0.84 ± 0.04	0.85 ± 0.09	0.44	1.07 ± 0.12	0.91 ± 0.09	1.12 ± 0.07	0.75 ± 0.02	0.88 ± 0.02	0.78 ± 0.07
S02	At3g28940	Arabidopsis thaliana (thale cress) AIG2 protein-like	205	19.5 / 17.0	5.0 / 5.1	0.08	2.42 ± 0.03	2.44 ± 0.16	1.04 ± 0.04	1.24 ± 0.01	0.81 ± 0.01	1.23 ± 0.05	0.06	1.08 ± 0.06	0.90 ± 0.05	1.26 ± 0.04	1.05 ± 0.11	1.52 ± 0.01	1.60 ± 0.03
S03	At3g14415	Peroxisomal (S)-2-hydroxy-acid oxidase GLO2	305	40.3 / 38.0	9.0 / 9.0	0.35	0.50 ± 0.13	0.50 ± 0.09	1.14 ± 0.09	1.18 ± 0.01	0.69 ± 0.02	0.96 ± 0.04	0.25	0.42 ± 0.07	0.50 ± 0.13	0.98 ± 0.03	1.09 ± 0.03	0.45 ± 0.04	0.46 ± 0.02
S04	At4g33010	Glycine dehydrogenase [decarboxylating] 2, mitochondrial	408	112.9 / 105.0	6.5 / 6.4	0.17	0.77 ± 0.13	0.51 ± 0.02	0.24 ± 0.00	0.52 ± 0.06	0.51 ± 0.09	0.69 ± 0.04	0.16	0.39 ± 0.04	0.50 ± 0.09	1.81 ± 0.08	2.06 ± 0.04	1.11 ± 0.07	0.86 ± 0.07
S05	At3g55440	Triosephosphate isomerase, cytosolic	401	27.5 / 22.0	5.4 / 5.5	0.59	0.99 ± 0.10	0.89 ± 0.23	0.89 ± 0.10	1.09 ± 0.11	2.01 ± 0.06	1.27 ± 0.02	0.42	0.82 ± 0.09	0.69 ± 0.01	1.25 ± 0.04	0.96 ± 0.04	1.11 ± 0.02	1.47 ± 0.06
S06	At1g63770	Putative aminopeptidase	506	99.2 / 98.0	5.4 / 5.6	0.15	0.77 ± 0.04	0.49 ± 0.04	1.02 ± 0.07	1.05 ± 0.04	0.44 ± 0.06	0.49 ± 0.04	0.10	0.38 ± 0.04	0.42 ± 0.02	0.11 ± 0.00	0.92 ± 0.16	3.88 ± 0.01	1.69 ± 0.09
S07	At3g60750	Putative transketolase	374	80.0 / 73.0	5.9 / 5.5	0.66	0.85 ± 0.08	0.57 ± 0.24	1.01 ± 0.03	0.95 ± 0.02	0.79 ± 0.01	0.78 ± 0.07	0.35	0.61 ± 0.07	0.79 ± 0.09	0.10 ± 0.02	0.85 ± 0.08	1.48 ± 0.05	1.23 ± 0.04
S08	At5g59880	Actin-depolymerizing factor 3	314	15.9 / 19.0	5.9 / 6.0	0.18	1.73 ± 0.02	2.19 ± 0.09	0.98 ± 0.11	1.42 ± 0.08	1.00 ± 0.06	1.16 ± 0.10	0.11	0.89 ± 0.04	0.75 ± 0.05	1.23 ± 0.01	1.13 ± 0.05	0.98 ± 0.05	0.91 ± 0.09
S09	At1g10960	Ferredoxin-1, chloroplastic	183	15.9 / 19.0	4.4 / 4.6	0.14	1.48 ± 0.02	1.97 ± 0.08	1.14 ± 0.04	1.34 ± 0.13	0.92 ± 0.05	0.84 ± 0.09	0.16	1.01 ± 0.04	1.18 ± 0.04	1.05 ± 0.03	1.25 ± 0.10	0.44 ± 0.04	0.35 ± 0.04
S10	At5g62530	Delta-1-pyrroline-5-carboxylate dehydrogenase 12A1, mitochondrial	124	61.8 / 58.0	6.3 / 6.2	0.03	0.81 ± 0.01	2.51 ± 0.14	1.07 ± 0.01	3.82 ± 0.11	0.23 ± 0.02	0.45 ± 0.01	0.02	0.87 ± 0.00	0.59 ± 0.00	0.39 ± 0.01	0.46 ± 0.01	0.15 ± 0.15	2.84 ± 0.07
S11	At2g30860	Glutathione S-transferase PHI 9	281	24.1 / 21.5	6.2 / 6.7	0.30	1.69 ± 0.04	1.79 ± 0.14	1.31 ± 0.08	2.25 ± 0.06	0.81 ± 0.04	0.92 ± 0.06	0.37	1.01 ± 0.06	1.49 ± 0.08	0.76 ± 0.09	1.05 ± 0.04	1.07 ± 0.03	0.80 ± 0.06
S12	N/A	**Not identified**	N/A	N/A / 18.5	N/A / 5.9	0.20	1.06 ± 0.01	1.81 ± 0.07	1.04 ± 0.07	1.2 ± 0.06	1.40 ± 0.03	1.47 ± 0.02	0.15	0.99 ± 0.08	0.66 ± 0.04	0.95 ± 0.02	0.97 ± 0.09	1.29 ± 0.02	1.15 ± 0.01
S13	At5g63310	Nucleoside diphosphate kinase II, chloroplastic	99	25.6 / 16.5	9.1 / 5.6	0.20	1.27 ± 0.03	1.73 ± 0.10	0.88 ± 0.04	1.32 ± 0.07	1.25 ± 0.04	1.14 ± 0.05	0.12	0.84 ± 0.04	0.80 ± 0.06	0.85 ± 0.03	0.59 ± 0.01	1.09 ± 0.02	0.90 ± 0.06
S14	At5g54770	Thiazole biosynthetic enzyme, chloroplastic	185	36.7 / 29.0	5.8 / 5.7	0.09	1.16 ± 0.02	1.41 ± 0.04	1.04 ± 0.13	1.38 ± 0.02	1.65 ± 0.05	1.79 ± 0.04	0.07	0.83 ± 0.01	0.83 ± 0.02	1.01 ± 0.03	1.21 ± 0.04	0.95 ± 0.06	1.29 ± 0.02
S15	At5g53490	Thylakoid lumenal 17.4 kDa protein, chloroplastic	240	25.6 / 15.5	6.0 / 5.4	0.16	1.03 ± 0.07	0.98 ± 0.07	1.08 ± 0.10	0.88 ± 0.05	1.54 ± 0.02	0.59 ± 0.03	0.10	0.98 ± 0.05	0.66 ± 0.02	0.03 ± 0.01	1.09 ± 0.05	1.20 ± 0.02	0.95 ± 0.03
S16	At4g25100	Superoxide dismutase [Fe], chloroplastic	139	23.8 / 19.5	6.6 / 6.2	0.50	1.62 ± 0.06	2.46 ± 0.27	0.95 ± 0.02	0.95 ± 0.02	1.02 ± 0.06	1.07 ± 0.08	0.34	0.94 ± 0.15	0.47 ± 0.06	0.86 ± 0.09	0.75 ± 0.14	1.43 ± 0.06	1.49 ± 0.05
S17	At3g52960	Peroxiredoxin-2E, chloroplastic	183	24.7 / 16.5	9.7 / 5.2	0.07	1.46 ± 0.05	1.59 ± 0.15	0.95 ± 0.04	1.50 ± 0.03	1.29 ± 0.02	1.15 ± 0.05	0.08	1.41 ± 0.06	1.02 ± 0.07	1.12 ± 0.05	1.40 ± 0.02	1.26 ± 0.03	0.71 ± 0.01
S18	At2g28190	Superoxide dismutase [Cu-Zn], chloroplastic	163	22.2 / 17.5	6.5 / 5.5	0.01	1.58 ± 0.04	1.51 ± 0.13	1.02 ± 0.07	1.42 ± 0.05	0.79 ± 0.03	1.13 ± 0.02	0.02	1.35 ± 0.09	1.11 ± 0.04	0.98 ± 0.05	1.40 ± 0.01	1.22 ± 0.23	1.32 ± 0.01
S19	At1g48030	Dihydrolipoyl dehydrogenase 1, mitochondrial	253	54.0 / 58.0	7.0 / 6.7	0.03	1.06 ± 0.01	1.25 ± 0.02	1.21 ± 0.07	0.86 ± 0.04	1.09 ± 0.07	1.76 ± 0.04	0.02	0.41 ± 0.01	0.54 ± 0.00	0.89 ± 0.02	0.94 ± 0.03	1.03 ± 0.01	1.11 ± 0.06
S20	At1g55480	GAN	131	37.4 / 33.0	8.2 / 5.4	0.06	0.84 ± 0.01	1.09 ± 0.01	0.91 ± 0.08	1.11 ± 0.03	2.25 ± 0.02	0.71 ± 0.11	0.03	0.51 ± 0.02	0.35 ± 0.02	0.97 ± 0.03	0.75 ± 0.05	1.29 ± 0.04	1.71 ± 0.10
S21	AtCg00490	Ribulose bisphosphate carboxylase large chain	578	53.0 / 51.0	5.9 / 6.2	1.61	0.76 ± 0.01	0.62 ± 0.02	1.09 ± 0.09	1.32 ± 0.02	0.84 ± 0.01	0.75 ± 0.00	2.29	1.63 ± 0.03	1.12 ± 0.02	0.90 ± 0.03	1.09 ± 0.09	0.78 ± 0.04	0.87 ± 0.05
S22	At1g06680	Oxygen-evolving enhancer protein 2-1, chloroplastic	209	28.1 / 24.0	6.9 / 5.4	0.16	1.28 ± 0.03	1.32 ± 0.06	1.24 ± 0.01	0.86 ± 0.03	1.27 ± 0.01	1.21 ± 0.01	0.24	1.71 ± 0.03	2.20 ± 0.03	1.3 ± 0.11	1.5 ± 0.01	0.92 ± 0.03	0.78 ± 0.09
S23	AT2G21660	CCR2 (COLD, CIRCADIAN RHYTHM, AND RNA BINDING 2)	66	14.9 / 16.5	5.1 / 5.5	0.18	0.94 ± 0.03	0.60 ± 0.05	0.67 ± 0.03	0.98 ± 0.04	0.65 ± 0.02	0.66 ± 0.01	0.08	1.00 ± 0.04	0.77 ± 0.07	1.17 ± 0.08	2.05 ± 0.05	2.36 ± 0.01	2.23 ± 0.02
S24	At4g28520	12S seed storage protein CRU1 (Cruciferin 1 or C)	232	58.2 / 59.0	6.5 / 6.5	0.24	0.96 ± 0.01	1.48 ± 0.26	1.11 ± 0.02	1.10 ± 0.07	0.57 ± 0.09	0.90 ± 0.10	0.34	1.36 ± 0.02	2.21 ± 0.02	0.98 ± 0.01	0.75 ± 0.01	1.39 ± 0.02	1.32 ± 0.05

S25	At1g72150	Patellin-1	304	64.0 / 105.0	4.8 / 5.2	0.22	0.76 ± 0.04	0.53 ± 0.07	1.2 ± 0.05	0.96 ± 0.04	0.71 ± 0.08	0.96 ± 0.02	0.18	0.76 ± 0.07	0.83 ± 0.04	1.02 ± 0.06	1.17 ± 0.07	1.51 ± 0.09	1.10 ± 0.01
S26	At1g20020	Ferredoxin--NADP reductase, leaf isozyme 2, chloroplastic	263	41.2 / 30.0	8.5 / 6.6	0.26	0.99 ± 0.02	1.47 ± 0.06	1.01 ± 0.01	1.3 ± 0.03	0.94 ± 0.09	1.20 ± 0.04	0.30	0.55 ± 0.05	0.55 ± 0.03	0.94 ± 0.03	1.21 ± 0.01	1.11 ± 0.07	1.08 ± 0.02
S27	At4g14880	Cysteine synthase	239	33.8 / 30.0	5.9 / 6.2	0.20	1.55 ± 0.06	1.03 ± 0.03	0.62 ± 0.03	0.59 ± 0.03	1.05 ± 0.08	1.02 ± 0.02	0.05	0.71 ± 0.02	0.41 ± 0.02	0.71 ± 0.02	0.50 ± 0.01	1.47 ± 0.05	1.25 ± 0.01
S28	At5g23120	Photosystem II stability/assembly factor HCF136, chloroplastic	395	44.1 / 36.5	6.8 / 5.4	0.02	0.82 ± 0.09	0.90 ± 0.11	1.2 ± 0.02	1.09 ± 0.04	1.83 ± 0.05	1.72 ± 0.02	0.06	1.02 ± 0.02	1.23 ± 0.10	1.11 ± 0.08	1.2 ± 0.03	1.02 ± 0.01	1.24 ± 0.00
S29	At5g04590	Sulphite reductase	292	72.0 / 65.0	8.5 / 7.7	0.42	0.48 ± 0.01	0.48 ± 0.01	0.52 ± 0.03	0.95 ± 0.04	0.67 ± 0.03	0.50 ± 0.05	0.20	0.99 ± 0.03	0.57 ± 0.01	1.24 ± 0.05	1.21 ± 0.03	0.84 ± 0.10	0.62 ± 0.08
S30	At5g66190	Ferredoxin--NADP reductase, leaf isozyme 1, chloroplastic	116	40.3 / 30.0	8.3 / 5.5	0.31	1.46 ± 0.04	1.46 ± 0.09	1.31 ± 0.03	0.65 ± 0.02	1.41 ± 0.03	1.17 ± 0.04	0.13	0.54 ± 0.03	0.48 ± 0.02	0.93 ± 0.03	0.48 ± 0.01	1.76 ± 0.02	2.18 ± 0.02
S31	At3g14930	Uroporphyrinogen decarboxylase 1, chloroplastic	274	46.3 / 42.0	6.6 / 6.6	0.29	1.65 ± 0.06	1.16 ± 0.07	1.21 ± 0.08	1.07 ± 0.06	0.68 ± 0.06	0.89 ± 0.05	0.25	1.51 ± 0.07	1.77 ± 0.05	0.96 ± 0.03	1.04 ± 0.05	0.85 ± 0.09	0.78 ± 0.08
S32	At2g13360	Serine--glyoxylate aminotransferase	543	44.2 / 45.0	7.7 / 8.1	0.05	1.14 ± 0.04	0.87 ± 0.04	1.09 ± 0.03	1.12 ± 0.04	0.38 ± 0.04	0.77 ± 0.01	0.05	1.00 ± 0.06	1.33 ± 0.05	0.89 ± 0.01	0.98 ± 0.05	1.07 ± 0.02	0.83 ± 0.02
S33	At1g09340	Uncharacterized protein At1g09340, chloroplastic	362	42.6 / 36.5	8.2 / 6.9	0.42	0.99 ± 0.03	0.66 ± 0.17	0.78 ± 0.06	0.72 ± 0.04	0.86 ± 0.06	1.06 ± 0.07	0.16	0.44 ± 0.06	0.37 ± 0.05	0.66 ± 0.09	1.04 ± 0.06	1.08 ± 0.06	1.34 ± 0.11
S34	At2g28000	RuBisCO large subunit-binding protein subunit alpha, chloroplastic	113	62.1 / 58.0	5.8 / 5.2	0.12	0.65 ± 0.02	0.49 ± 0.05	1.03 ± 0.05	0.94 ± 0.09	0.40 ± 0.03	0.71 ± 0.05	0.25	1.00 ± 0.03	1.97 ± 0.06	1.13 ± 0.02	0.91 ± 0.03	0.99 ± 0.09	0.85 ± 0.17
S35	At2g21330	Fructose-bisphosphate aldolase	155	33.3 / 34.0	6.3 / 5.4	0.17	0.96 ± 0.06	1.27 ± 0.09	1.08 ± 0.04	1.13 ± 0.04	1.43 ± 0.05	1.60 ± 0.02	0.14	0.69 ± 0.04	0.51 ± 0.04	1.07 ± 0.16	1.2 ± 0.04	1.37 ± 0.07	1.14 ± 0.07
S36	At5g17920	5-methyltetrahydropteroyltriglutamate--homocysteine methyltransferase	677	84.4 / 75.0	6.9 / 6.6	1.11	0.93 ± 0.15	0.80 ± 0.09	0.27 ± 0.02	0.45 ± 0.07	0.31 ± 0.05	0.82 ± 0.03	0.79	0.46 ± 0.02	0.60 ± 0.02	2.76 ± 0.17	2.67 ± 0.06	1.29 ± 0.07	0.99 ± 0.01
S37	At3g14210	GDSL esterase/lipase ESM1	481	44.1 / 45.0	7.8 / 7.3	1.61	1.01 ± 0.32	1.07 ± 0.66	0.98 ± 0.04	1.18 ± 0.07	1.08 ± 0.05	1.31 ± 0.07	1.85	0.89 ± 0.05	0.74 ± 0.04	0.74 ± 0.04	1.14 ± 0.01	0.91 ± 0.23	0.81 ± 0.01
S38	At1g77510	Protein disulfide isomerase-like 1-2	109	56.4 / 65.0	4.9 / 4.2	0.25	1.86 ± 0.01	2.07 ± 0.05	1.08 ± 0.07	0.92 ± 0.08	0.75 ± 0.01	1.36 ± 0.03	0.30	1.79 ± 0.01	1.57 ± 0.01	1.18 ± 0.01	0.88 ± 0.03	1.07 ± 0.03	0.74 ± 0.06
S39	At1g68010	Hydroxypyruvate reductase	356	42.2 / 38.0	6.7 / 7.2	0.43	0.78 ± 0.06	1.03 ± 0.09	1.13 ± 0.04	1.20 ± 0.02	0.65 ± 0.07	0.89 ± 0.10	0.46	0.61 ± 0.01	0.55 ± 0.02	1.05 ± 0.03	0.99 ± 0.08	1.46 ± 0.08	1.19 ± 0.05
S40	At5g11670	Malic enzyme	568	64.4 / 65.0	6.1 / 6.3	0.29	1.28 ± 0.01	1.12 ± 0.13	0.95 ± 0.05	1.05 ± 0.13	0.85 ± 0.05	1.22 ± 0.06	0.20	0.57 ± 0.03	0.64 ± 0.03	1.24 ± 0.01	0.94 ± 0.03	1.58 ± 0.01	1.05 ± 0.07
S41	At2g37660	Uncharacterized protein At2g37660, chloroplastic	258	34.9 / 22.5	8.4 / 5.5	0.13	1.05 ± 0.05	1.08 ± 0.04	0.96 ± 0.05	0.88 ± 0.02	1.24 ± 0.11	1.19 ± 0.66	0.12	1.01 ± 0.03	0.98 ± 0.07	1.22 ± 0.01	1.21 ± 0.07	1.11 ± 0.10	1.48 ± 0.04
S42	At2g43750	Cysteine synthase, chloroplastic/chromoplastic	70	41.7 / 32.0	8.1 / 5.9	0.22	0.87 ± 0.03	1.22 ± 0.02	1.10 ± 0.08	0.94 ± 0.02	2.07 ± 0.02	1.51 ± 0.05	0.08	1.25 ± 0.02	1.09 ± 0.04	1.50 ± 0.12	1.2 ± 0.05	1.67 ± 0.06	1.90 ± 0.06
S43	At3g16640	Translationally-controlled tumor protein homolog	363	18.9 / 18.5	4.5 / 4.8	0.50	1.08 ± 0.12	1.32 ± 0.15	0.85 ± 0.06	1.11 ± 0.03	0.68 ± 0.02	1.68 ± 0.03	0.36	0.91 ± 0.05	0.93 ± 0.12	1.50 ± 0.05	0.87 ± 0.01	0.89 ± 0.02	0.59 ± 0.02
S44	At3g55800	Sedoheptulose-1,7-bisphosphatase, chloroplastic	275	42.4 / 38.0	6.2 / 5.2	0.03	0.87 ± 0.04	1.03 ± 0.04	1.25 ± 0.02	1.04 ± 0.07	0.77 ± 0.14	1.32 ± 0.07	0.04	0.37 ± 0.05	0.35 ± 0.02	1.14 ± 0.08	0.95 ± 0.04	1.13 ± 0.00	0.92 ± 0.05
S45	At2g21330	Fructose-bisphosphate aldolase	323	33.3 / 34.0	5.3 / 5.4	1.16	0.89 ± 0.14	1.09 ± 0.21	0.72 ± 0.03	1.03 ± 0.09	1.22 ± 0.02	1.50 ± 0.06	1.12	0.66 ± 0.11	0.67 ± 0.02	1.39 ± 0.02	0.91 ± 0.08	1.15 ± 0.08	1.17 ± 0.09
S46	At2g41530	S-formylglutathione hydrolase	402	31.7 / 26.0	5.9 / 6.2	0.17	1.34 ± 0.07	2.45 ± 0.02	1.15 ± 0.02	0.95 ± 0.06	1.25 ± 0.07	1.58 ± 0.01	0.11	1.01 ± 0.09	0.96 ± 0.03	1.47 ± 0.01	0.78 ± 0.02	1.55 ± 0.01	1.53 ± 0.05
S47	At2g40490	Uroporphyrinogen decarboxylase 2, chloroplastic	129	43.6 / 38.0	8.3 / 6.6	0.22	1.07 ± 0.02	0.83 ± 0.01	1.05 ± 0.06	1.07 ± 0.04	0.77 ± 0.06	0.66 ± 0.04	0.20	1.18 ± 0.01	1.22 ± 0.02	0.97 ± 0.13	1.26 ± 0.12	0.82 ± 0.05	1.18 ± 0.09
S48	At5g14740	Carbonic anhydrase 2, chloroplastic	397	28.3 / 29.0	5.4 / 5.5	0.53	1.42 ± 0.09	1.36 ± 0.06	0.83 ± 0.07	0.99 ± 0.04	1.16 ± 0.10	1.16 ± 0.02	0.36	0.88 ± 0.07	0.81 ± 0.18	0.90 ± 0.00	1.15 ± 0.06	1.04 ± 0.02	1.29 ± 0.07
S49	At5g36700	4-nitrophenylphosphatase-like	180	43.1 / 60.0	8.8 / 5.9	0.54	1.77 ± 0.35	1.50 ± 0.07	1.15 ± 0.09	1.33 ± 0.13	1.71 ± 0.03	1.41 ± 0.02	0.38	1.07 ± 0.14	0.99 ± 0.11	1.21 ± 0.07	1.21 ± 0.03	1.11 ± 0.02	0.80 ± 0.03
S50	At5g20630	Germin-like protein subfamily 3 member 3	110	21.8 / 18.5	6.3 / 6.2	0.03	0.65 ± 0.04	1.18 ± 0.02	1.28 ± 0.11	1.11 ± 0.06	1.05 ± 0.09	1.53 ± 0.01	0.07	1.05 ± 0.02	0.74 ± 0.02	1.09 ± 0.04	0.95 ± 0.05	1.27 ± 0.09	0.95 ± 0.03
S51	At4g39260	Glycine-rich RNA-binding protein 8	217	16.6 / 16.5	5.6 / 5.4	0.20	0.93 ± 0.03	1.78 ± 0.08	1.08 ± 0.05	1.06 ± 0.10	1.41 ± 0.08	1.10 ± 0.15	0.27	0.86 ± 0.05	0.76 ± 0.12	1.29 ± 0.01	0.97 ± 0.01	1.09 ± 0.05	1.36 ± 0.02
S52	At3g62030	Peptidyl-prolyl cis-trans isomerase CYP20-3, chloroplastic	282	28.2 / 17.5	8.8 / 5.6	0.08	0.85 ± 0.13	0.62 ± 0.01	1.14 ± 0.03	0.65 ± 0.11	1.32 ± 0.13	1.24 ± 0.01	0.06	0.89 ± 0.10	0.89 ± 0.20	1.05 ± 0.01	0.77 ± 0.01	1.03 ± 0.07	0.99 ± 0.03
S53	At3g08740	Putative elongation factor P (EF-P)	198	26.2 / 19.0	8.6 / 5.4	0.04	1.95 ± 0.01	2.43 ± 0.02	0.74 ± 0.02	1.09 ± 0.02	1.01 ± 0.04	1.12 ± 0.05	0.04	1.31 ± 0.03	1.23 ± 0.03	0.76 ± 0.02	0.34 ± 0.01	2.37 ± 0.12	2.17 ± 0.01
S54	At4g09010	Thylakoid lumenal 29 kDa protein, chloroplastic	252	37.9 / 22.5	8.6 / 6.2	0.07	0.69 ± 0.06	0.59 ± 0.06	1.21 ± 0.02	1.06 ± 0.02	1.09 ± 0.02	1.73 ± 0.14	0.06	0.69 ± 0.06	0.94 ± 0.05	1.19 ± 0.04	1.03 ± 0.08	2.01 ± 0.07	2.14 ± 0.03

S55	At5g09650	Soluble inorganic pyrophosphatase 1, chloroplastic	236	33.4 / 26.0	5.7 / 5.2	0.10	0.95 ± 0.09	0.80 ± 0.08	1.11 ± 0.03	0.97 ± 0.07	0.84 ± 0.03	1.38 ± 0.04	0.08	1.12 ± 0.07	1.31 ± 0.10	1.06 ± 0.01	0.92 ± 0.01	1.02 ± 0.06	0.87 ± 0.06
S56	At1g65930	Isocitrate dehydrogenase [NADP]	238	45.7 / 46.5	6.1 / 6.4	0.33	1.36 ± 0.07	1.09 ± 0.10	1.01 ± 0.04	0.97 ± 0.03	0.93 ± 0.06	1.40 ± 0.15	0.45	0.90 ± 0.04	0.80 ± 0.02	0.99 ± 0.02	1.11 ± 0.05	1.21 ± 0.13	1.21 ± 0.02
S57	At3g55800	Sedoheptulose-1,7-bisphosphatase, chloroplastic	497	42.4 / 36.5	6.2 / 5.3	0.16	1.11 ± 0.24	0.87 ± 0.14	0.75 ± 0.07	0.65 ± 0.12	0.92 ± 0.06	1.42 ± 0.11	0.21	0.95 ± 0.07	0.85 ± 0.19	1.37 ± 0.09	1.22 ± 0.08	0.99 ± 0.15	0.87 ± 0.10
S58	At5g25980	Myrosinase 2	64	62.7 / 58.0	7.1 / 6.6	0.13	1.09 ± 0.02	1.22 ± 0.03	1.07 ± 0.05	1.09 ± 0.04	0.50 ± 0.02	1.04 ± 0.03	0.07	1.04 ± 0.02	1.15 ± 0.02	1.22 ± 0.04	1.02 ± 0.03	1.05 ± 0.03	0.84 ± 0.04
S59	At3g01500	Carbonic anhydrase, chloroplastic	276	37.5 / 22.0	5.7 / 6.6	0.06	0.89 ± 0.02	1.43 ± 0.05	0.93 ± 0.02	0.92 ± 0.13	0.89 ± 0.07	1.43 ± 0.07	0.05	0.86 ± 0.06	0.76 ± 0.04	0.88 ± 0.14	1.20 ± 0.10	0.84 ± 0.04	0.76 ± 0.04
S60	At3g50820	Oxygen-evolving enhancer protein 1-2, chloroplastic	155	35.0 / 26.0	5.9 / 5.3	0.46	0.78 ± 0.02	0.96 ± 0.06	1.11 ± 0.04	1.01 ± 0.03	1.41 ± 0.03	1.55 ± 0.11	0.50	1.00 ± 0.04	0.99 ± 0.13	1.06 ± 0.03	0.99 ± 0.01	0.97 ± 0.01	0.79 ± 0.09
S61	At3g12780	Phosphoglycerate kinase 1, chloroplastic	432	50.0 / 45.0	5.9 / 5.3	0.02	0.66 ± 0.01	0.43 ± 0.02	0.96 ± 0.04	0.66 ± 0.05	0.86 ± 0.04	0.66 ± 0.04	0.01	1.02 ± 0.11	1.13 ± 0.15	0.88 ± 0.02	1.00 ± 0.00	1.04 ± 0.06	0.70 ± 0.06
S62	At4g38970	Probable fructose-bisphosphate aldolase 2, chloroplastic	194	42.9 / 32.0	6.8 / 5.6	0.57	1.46 ± 0.05	1.47 ± 0.04	1.01 ± 0.10	1.12 ± 0.01	1.41 ± 0.12	1.17 ± 0.02	0.41	0.52 ± 0.03	0.42 ± 0.06	1.07 ± 0.01	1.06 ± 0.11	1.96 ± 0.08	2.44 ± 0.12
S63	At1g57720	Probable elongation factor 1-gamma 2	316	46.4 / 48.5	5.6 / 5.7	0.01	0.76 ± 0.04	0.88 ± 0.04	1.19 ± 0.04	0.97 ± 0.03	0.95 ± 0.01	1.14 ± 0.07	0.01	1.40 ± 0.03	1.42 ± 0.06	0.50 ± 0.02	0.59 ± 0.01	2.06 ± 0.15	2.51 ± 0.02
S64	At4g38970	Probable fructose-bisphosphate aldolase 2, chloroplastic	519	42.9 / 34.0	6.8 / 5.6	0.23	1.05 ± 0.31	1.02 ± 0.16	1.21 ± 0.02	1.26 ± 0.05	1.22 ± 0.06	1.46 ± 0.09	0.26	1.05 ± 0.16	1.19 ± 0.28	0.97 ± 0.09	1.15 ± 0.01	1.22 ± 0.02	1.28 ± 0.04
S65	At3g55800	Sedoheptulose-1,7-bisphosphatase, chloroplastic	390	42.4 / 77.0	6.2 / 6.7	0.92	1.01 ± 0.03	0.88 ± 0.04	0.72 ± 0.08	0.96 ± 0.04	0.64 ± 0.04	0.65 ± 0.03	0.92	1.15 ± 0.01	0.87 ± 0.02	0.87 ± 0.05	1.21 ± 0.05	0.90 ± 0.06	0.67 ± 0.04
S66	N/A	**Not identified**	N/A	N/A / 70.0	N/A / 6.7	0.05	1.62 ± 0.01	1.04 ± 0.01	0.53 ± 0.01	0.69 ± 0.01	0.88 ± 0.01	0.52 ± 0.00	0.05	0.54 ± 0.01	0.48 ± 0.02	0.61 ± 0.01	1.26 ± 0.02	1.15 ± 0.04	1.35 ± 0.14
S67	At5g65730	Probable xyloglucan endotransglucosylase/hydrolase protein 6	32	33.7 / 26.0	6.7 / 6.8	0.48	1.12 ± 0.01	1.17 ± 0.00	1.06 ± 0.03	1.08 ± 0.02	1.36 ± 0.09	1.71 ± 0.02	0.29	2.23 ± 0.01	2.38 ± 0.01	1.14 ± 0.01	1.14 ± 0.09	1.58 ± 0.04	1.50 ± 0.02
S68	At3g54050	Fructose-1,6-bisphosphatase, chloroplastic	308	45.2 / 47.0	5.3 / 5.1	0.23	0.97 ± 0.13	1.16 ± 0.10	1.15 ± 0.07	0.85 ± 0.05	0.44 ± 0.66	1.13 ± 0.06	0.11	0.84 ± 0.07	0.83 ± 0.09	1.05 ± 0.02	1.02 ± 0.01	1.08 ± 0.04	0.92 ± 0.03
S69	At1g53240	Malate dehydrogenase 1, mitochondrial	273	35.8 / 32.0	8.5 / 6.4	0.04	0.90 ± 0.08	0.91 ± 0.05	1.21 ± 0.03	1.05 ± 0.04	1.10 ± 0.08	1.38 ± 0.03	0.04	1.44 ± 0.05	1.72 ± 0.07	1.28 ± 0.01	1.12 ± 0.03	1.01 ± 0.07	0.98 ± 0.06
S70	At4g29060	Elongation factor Ts	113	103.8 / 95.0	5.0 / 5.0	0.08	1.76 ± 0.01	1.85 ± 0.01	0.93 ± 0.02	1.05 ± 0.05	1.20 ± 0.03	1.23 ± 0.01	0.08	1.49 ± 0.01	1.53 ± 0.01	0.88 ± 0.09	1.12 ± 0.01	1.66 ± 0.04	1.03 ± 0.14
S71	At1g18080	Guanine nucleotide-binding protein subunit beta-like protein A	350	35.7 / 34.0	7.6 / 8.5	0.10	0.63 ± 0.03	0.71 ± 0.04	0.72 ± 0.13	1.06 ± 0.04	0.63 ± 0.06	0.71 ± 0.02	0.05	0.75 ± 0.02	1.54 ± 0.03	0.45 ± 0.03	0.41 ± 0.01	0.87 ± 0.15	1.41 ± 0.13
S72	N/A	**Not identified**	N/A	N/A / 18.5	N/A / 5.9	0.02	1.01 ± 0.01	1.05 ± 0.05	1.30 ± 0.07	0.94 ± 0.12	1.40 ± 0.05	1.47 ± 0.09	0.02	2.05 ± 0.02	1.59 ± 0.06	1.12 ± 0.01	1.33 ± 0.08	1.15 ± 0.01	0.97 ± 0.02
S73	At4g29060	Elongation factor Ts	113	103.8 / 145.0	5.0 / 5.2	0.31	0.67 ± 0.05	1.18 ± 0.08	0.40 ± 0.01	0.94 ± 0.02	0.49 ± 0.00	1.12 ± 0.02	0.32	1.34 ± 0.04	1.50 ± 0.07	0.72 ± 0.02	0.46 ± 0.01	1.46 ± 0.08	1.06 ± 0.00
S74	At4g23670	Major latex protein-related / MLP-related	101	17.5 / 16.0	5.9 / 6.2	0.07	0.86 ± 0.05	1.34 ± 0.11	2.10 ± 0.04	1.98 ± 0.02	1.30 ± 0.09	1.34 ± 0.17	0.09	1.26 ± 0.06	1.37 ± 0.11	0.86 ± 0.04	0.87 ± 0.03	1.36 ± 0.02	1.12 ± 0.05
S75	At4g39260	Glycine-rich RNA-binding protein 8	107	16.6 / 17.5	5.6 / 5.4	0.23	1.03 ± 0.02	1.2 ± 0.03	1.17 ± 0.03	1.62 ± 0.01	1.14 ± 0.03	1.01 ± 0.08	0.38	1.07 ± 0.04	0.99 ± 0.01	0.62 ± 0.04	0.46 ± 0.02	0.94 ± 0.03	1.06 ± 0.07
S76	At3g57260	Glucan endo-1,3-beta-glucosidase, acidic isoform	124	37.3 / 30.0	4.9 / 5.0	0.02	1.10 ± 0.03	1.06 ± 0.01	0.65 ± 0.04	0.93 ± 0.01	1.09 ± 0.04	1.06 ± 0.09	0.03	0.98 ± 0.03	1.01 ± 0.01	0.77 ± 0.03	0.65 ± 0.01	0.89 ± 0.12	1.03 ± 0.04
S77	At1g09750	Nucleoid DNA-binding-like protein	99	47.7 / 48.5	7.5 / 7.7	0.29	1.07 ± 0.35	1.07 ± 0.00	0.97 ± 0.03	0.85 ± 0.03	1.11 ± 0.21	0.96 ± 0.02	0.36	0.91 ± 0.14	1.22 ± 0.08	0.58 ± 0.03	0.17 ± 0.01	1.01 ± 0.02	1.22 ± 0.07
S78	At1g56340	Calreticulin-1	152	48.5 / 58.0	4.5 / 4.8	0.08	0.93 ± 0.06	0.88 ± 0.02	0.79 ± 0.04	1.39 ± 0.04	1.3 ± 0.10	1.05 ± 0.09	0.10	1.05 ± 0.15	1.01 ± 0.07	0.81 ± 0.04	0.57 ± 0.03	0.89 ± 0.01	1.04 ± 0.04
S79	At1g78380	Glutathione S-transferase TAU 19	122	25.7 / 21.5	5.9 / 5.7	0.58	1.12 ± 0.01	1.25 ± 0.02	0.85 ± 0.02	1.01 ± 0.02	1.21 ± 0.05	0.98 ± 0.06	0.47	0.9 ± 0.06	1.04 ± 0.03	1.01 ± 0.02	0.54 ± 0.01	1.28 ± 0.06	1.17 ± 0.09
S80	At1g20620	Catalase-3	184	56.7 / 55.0	7.3 / 8.0	0.14	0.93 ± 0.03	1.01 ± 0.03	0.48 ± 0.02	1.02 ± 0.14	1.22 ± 0.06	0.93 ± 0.04	0.20	1.05 ± 0.01	0.99 ± 0.04	0.67 ± 0.04	0.83 ± 0.05	1.13 ± 0.18	1.11 ± 0.09
S81	At4g09320	Nucleoside diphosphate kinase 1	316	16.5 / 14.5	6.3 / 6.5	0.27	0.89 ± 0.07	1.03 ± 0.04	1.60 ± 0.15	1.85 ± 0.19	1.06 ± 0.06	0.92 ± 0.05	0.34	1.21 ± 0.04	0.97 ± 0.09	2.06 ± 0.21	1.07 ± 0.03	1.15 ± 0.03	1.21 ± 0.04
S82	At4g14880	Cysteine synthase	129	33.8 / 32.0	5.9 / 6.2	0.15	1.28 ± 0.03	0.93 ± 0.08	0.35 ± 0.00	1.10 ± 0.01	1.06 ± 0.13	1.11 ± 0.07	0.19	0.89 ± 0.04	1.05 ± 0.07	1.32 ± 0.00	0.78 ± 0.00	1.15 ± 0.07	0.95 ± 0.04
S83	N/A	**Not identified**	N/A	N/A / 30.0	N/A / 6.9	0.28	0.97 ± 0.02	1.05 ± 0.04	4.17 ± 0.05	2.51 ± 0.04	1.07 ± 0.06	0.88 ± 0.04	0.35	1.11 ± 0.06	1.06 ± 0.05	0.98 ± 0.02	0.88 ± 0.01	1.16 ± 0.05	1.01 ± 0.06
S84	At1g30580	GTP-binding protein	280	44.5 / 46.0	6.4 / 7.2	0.04	0.94 ± 0.07	0.97 ± 0.10	0.61 ± 0.03	0.67 ± 0.03	1.12 ± 0.02	0.93 ± 0.07	0.06	1.16 ± 0.04	0.92 ± 0.02	1.88 ± 0.08	1.76 ± 0.05	0.91 ± 0.04	0.88 ± 0.06

S85	At4g02520	Atpm24.1 glutathione S transferase	451	24.1 / 21.5	6.1 / 6.3	0.21	**1.14 ± 0.02**	**0.98 ± 0.04**	**1.47 ± 0.03**	**1.51 ± 0.04**	**1.21 ± 0.07**	**1.05 ± 0.03**	0.35	**0.94 ± 0.08**	**1.24 ± 0.06**	**1.03 ± 0.01**	**1.09 ± 0.04**	**1.19 ± 0.04**	**0.91 ± 0.04**
S86	At2g37220	Ribonucleoprotein At2g37220, chloroplastic	63	30.7 / 22.5	5.6 / 4.9	0.36	**0.90 ± 0.03**	**1.22 ± 0.01**	**0.48 ± 0.04**	**0.50 ± 0.10**	**1.07 ± 0.08**	**1.12 ± 0.11**	0.36	**1.13 ± 0.02**	**1.01 ± 0.05**	**0.96 ± 0.04**	**0.73 ± 0.06**	**1.02 ± 0.03**	**0.91 ± 0.01**
S87	At3g12580	Heat shock cognate 70 kDa protein 4	296	71.1 / 73.0	5.2 / 5.3	0.07	**1.14 ± 0.02**	**1.06 ± 0.01**	**0.30 ± 0.02**	**1.20 ± 0.15**	**1.21 ± 0.04**	**1.25 ± 0.05**	0.11	**1.05 ± 0.07**	**1.21 ± 0.11**	**1.05 ± 0.07**	**1.41 ± 0.08**	**1.19 ± 0.06**	**1.04 ± 0.13**
S88	At1g06680	Oxygen-evolving enhancer protein 2-1, chloroplastic	119	28.1 / 20.0	6.9 / 5.4	0.27	**1.01 ± 0.06**	**1.1 ± 0.03**	**0.70 ± 0.12**	**1.12 ± 0.15**	**1.23 ± 0.04**	**0.97 ± 0.04**	0.26	**0.92 ± 0.05**	**1.02 ± 0.02**	**0.94 ± 0.26**	**0.77 ± 0.12**	**1.07 ± 0.03**	**0.92 ± 0.05**
S89	At1g19130	Putative uncharacterized protein At1g19130/F14D16_18	70	21.6 / 19.5	5.5 / 5.5	0.45	**1.16 ± 0.07**	**1.3 ± 0.06**	**2.04 ± 0.01**	**1.30 ± 0.00**	**1.11 ± 0.09**	**1.01 ± 0.02**	0.44	**1.05 ± 0.01**	**0.89 ± 0.15**	**1.38 ± 0.01**	**0.48 ± 0.00**	**1.06 ± 0.02**	**0.92 ± 0.06**
S90	N/A	**Not identified**	N/A	N/A / 65.0	N/A / 7.9	0.15	**0.99 ± 0.09**	**0.97 ± 0.12**	**0.67 ± 0.01**	**0.97 ± 0.02**	**0.98 ± 0.01**	**1.05 ± 0.01**	0.18	**0.93 ± 0.07**	**1.14 ± 0.08**	**1.03 ± 0.02**	**1.05 ± 0.01**	**0.92 ± 0.02**	**1.12 ± 0.05**
S91	At5g25980	Myrosinase 2	144	62.7 / 60.0	7.1 / 6.8	0.17	**0.41 ± 0.02**	**0.39 ± 0.05**	**0.97 ± 0.11**	**1.03 ± 0.01**	**0.64 ± 0.02**	**0.97 ± 0.14**	0.17	**0.28 ± 0.04**	**0.37 ± 0.02**	**0.92 ± 0.01**	**0.97 ± 0.03**	**1.04 ± 0.14**	**0.99 ± 0.00**
S92	At3g58610	Ketol-acid reductoisomerase, chloroplastic	173	63.8 / 58.0	6.4 / 5.9	3.75	**0.93 ± 0.02**	**1.01 ± 0.02**	**0.49 ± 0.02**	**0.87 ± 0.02**	**0.93 ± 0.07**	**1.03 ± 0.10**	3.10	**1.28 ± 0.02**	**1.06 ± 0.07**	**1.24 ± 0.04**	**0.99 ± 0.02**	**1.05 ± 0.04**	**0.97 ± 0.02**
S93	At3g63140	Uncharacterized protein At3g63140, chloroplastic	361	43.9 / 36.5	8.5 / 6.5	0.03	**1.07 ± 0.04**	**1.28 ± 0.09**	**1.93 ± 0.06**	**2.50 ± 0.11**	**0.94 ± 0.08**	**1.06 ± 0.10**	0.03	**0.91 ± 0.04**	**0.89 ± 0.06**	**0.86 ± 0.05**	**0.81 ± 0.06**	**1.16 ± 0.03**	**1.03 ± 0.09**
S94	N/A	**Not identified**	N/A	N/A / 65.0	N/A / 8.0	0.30	**0.88 ± 0.02**	**1.05 ± 0.02**	**0.52 ± 0.01**	**0.98 ± 0.01**	**1.21 ± 0.02**	**0.9 ± 0.06**	0.46	**1.01 ± 0.01**	**1.04 ± 0.14**	**1.10 ± 0.01**	**0.93 ± 0.01**	**1.19 ± 0.03**	**1.13 ± 0.09**
S95	At1g04410	Malate dehydrogenase, cytoplasmic 1	81	35.6 / 36.5	6.1 / 6.3	0.08	**1.23 ± 0.12**	**1.21 ± 0.05**	**1.26 ± 0.04**	**0.58 ± 0.01**	**0.97 ± 0.06**	**1.28 ± 0.09**	0.12	**1.07 ± 0.08**	**1.28 ± 0.04**	**1.26 ± 0.03**	**0.92 ± 0.02**	**1.14 ± 0.06**	**0.89 ± 0.01**
S96	At5g25980	Myrosinase 2	577	62.7 / 60.0	7.1 / 7.0	0.04	**1.13 ± 0.14**	**1.12 ± 0.07**	**0.35 ± 0.06**	**0.65 ± 0.06**	**0.89 ± 0.00**	**1.21 ± 0.05**	0.04	**1.01 ± 0.11**	**0.91 ± 0.04**	**0.64 ± 0.09**	**0.48 ± 0.05**	**1.1 ± 0.01**	**0.97 ± 0.05**
S97	At1g56075	Elongation factor EF-2	392	93.9 / 90.0	5.9 / 6.5	0.05	**0.91 ± 0.06**	**1.19 ± 0.13**	**0.55 ± 0.01**	**0.61 ± 0.01**	**0.92 ± 0.06**	**1.3 ± 0.06**	0.04	**0.97 ± 0.09**	**1.11 ± 0.10**	**2.24 ± 0.02**	**1.01 ± 0.01**	**1.14 ± 0.04**	**0.89 ± 0.03**
S98	At4g23670	Major latex protein-related / MLP-related	132	17.5 / 16.0	5.9 / 6.3	0.05	**1.06 ± 0.03**	**0.93 ± 0.03**	**0.71 ± 0.09**	**1.66 ± 0.36**	**0.96 ± 0.01**	**0.95 ± 0.03**	0.03	**1.15 ± 0.02**	**0.88 ± 0.09**	**0.87 ± 0.04**	**0.41 ± 0.02**	**1.22 ± 0.02**	**1.06 ± 0.02**
S99	N/A	**Not identified**	N/A	N/A / 26.0	N/A / 7.2	0.32	**0.95 ± 0.05**	**1.15 ± 0.04**	**0.90 ± 0.02**	**1.39 ± 0.02**	**1.01 ± 0.05**	**1.07 ± 0.05**	0.35	**1.06 ± 0.03**	**1.19 ± 0.01**	**0.98 ± 0.05**	**0.89 ± 0.01**	**0.92 ± 0.06**	**0.91 ± 0.02**
S100	N/A	**Not identified**	N/A	N/A / 155.0	N/A / 4.9	0.10	**0.89 ± 0.01**	**1.14 ± 0.08**	**0.53 ± 0.01**	**1.35 ± 0.03**	**0.93 ± 0.03**	**0.92 ± 0.04**	0.15	**1.25 ± 0.11**	**1.05 ± 0.10**	**0.83 ± 0.03**	**0.73 ± 0.01**	**0.88 ± 0.06**	**1.14 ± 0.06**
S101	AtCg00490	Ribulose bisphosphate carboxylase large chain	86	53.0 / 36.5	5.9 / 6.7	0.04	**0.95 ± 0.02**	**1.22 ± 0.04**	**1.49 ± 0.06**	**0.94 ± 0.03**	**1.05 ± 0.15**	**1.1 ± 0.05**	0.04	**1.18 ± 0.14**	**1.01 ± 0.06**	**1.49 ± 0.04**	**0.83 ± 0.02**	**1.04 ± 0.02**	**0.98 ± 0.06**
S102	At3g02360	6-phosphogluconate dehydrogenase, decarboxylating	288	53.6 / 48.5	7.2 / 7.3	0.10	**1.09 ± 0.06**	**1.06 ± 0.03**	**0.71 ± 0.00**	**1.35 ± 0.02**	**1.01 ± 0.03**	**1.01 ± 0.02**	0.21	**1.11 ± 0.13**	**1.03 ± 0.02**	**1.08 ± 0.02**	**1.13 ± 0.02**	**0.95 ± 0.03**	**0.99 ± 0.02**
S103	At1g70730	Probable phosphoglucomutase, cytoplasmic 2	106	63.5 / 70.0	5.6 / 5.7	0.05	**1.04 ± 0.04**	**1.28 ± 0.01**	**0.51 ± 0.01**	**1.79 ± 0.02**	**1.11 ± 0.05**	**1.01 ± 0.10**	0.03	**1.12 ± 0.09**	**1.07 ± 0.03**	**0.55 ± 0.01**	**0.88 ± 0.01**	**1.06 ± 0.06**	**0.95 ± 0.01**
S104	At1g56410	Heat shock cognate 70 kDa protein/HSC70/HSP70	68	68.4 / 70.0	5.2 / 5.3	1.88	**1.06 ± 0.02**	**1.11 ± 0.04**	**0.46 ± 0.08**	**1.01 ± 0.17**	**1.25 ± 0.11**	**1.07 ± 0.02**	2.15	**1.15 ± 0.03**	**0.95 ± 0.09**	**0.95 ± 0.18**	**1.45 ± 0.08**	**1.04 ± 0.01**	**1.22 ± 0.05**
S105	At5g66190	Ferredoxin--NADP reductase, leaf isozyme 1, chloroplastic	117	40.3 / 30.0	8.3 / 5.7	0.30	**1.03 ± 0.00**	**0.99 ± 0.05**	**0.66 ± 0.03**	**0.91 ± 0.03**	**0.93 ± 0.03**	**1.17 ± 0.09**	0.23	**1.21 ± 0.08**	**0.93 ± 0.02**	**1.44 ± 0.02**	**0.84 ± 0.01**	**1.05 ± 0.06**	**1.21 ± 0.02**
S106	AtCg00490	Ribulose bisphosphate carboxylase large chain	96	53.0 / 36.5	5.9 / 6.6	0.22	**1.17 ± 0.02**	**1.07 ± 0.06**	**10.06 ± 0.09**	**2.76 ± 0.02**	**1.1 ± 0.08**	**0.97 ± 0.02**	0.29	**1.21 ± 0.10**	**0.91 ± 0.01**	**1.08 ± 0.02**	**0.62 ± 0.01**	**0.98 ± 0.02**	**0.92 ± 0.05**
S107	At5g02240	Uncharacterized protein At5g02240	106	27.1 / 24.5	6.2 / 6.2	0.09	**1.19 ± 0.02**	**1.01 ± 0.02**	**1.62 ± 0.07**	**1.48 ± 0.09**	**1.17 ± 0.14**	**1.05 ± 0.01**	0.08	**1.2 ± 0.05**	**0.92 ± 0.07**	**0.95 ± 0.04**	**0.57 ± 0.03**	**1.14 ± 0.00**	**0.95 ± 0.11**
S108	At3g15356	Lectin-like protein	243	29.7 / 26.0	8.9 / 9.4	0.11	**1.24 ± 0.06**	**0.91 ± 0.06**	**0.83 ± 0.02**	**1.75 ± 0.03**	**1.01 ± 0.02**	**0.91 ± 0.14**	0.11	**1.3 ± 0.10**	**0.97 ± 0.05**	**0.46 ± 0.02**	**0.42 ± 0.01**	**0.99 ± 0.07**	**1.01 ± 0.03**
S109	At1g21750	Protein disulfide isomerase-like 1-1	347	55.6 / 105.0	4.8 / 5.9	0.09	**0.95 ± 0.01**	**1.09 ± 0.03**	**0.19 ± 0.00**	**1.15 ± 0.02**	**0.91 ± 0.06**	**0.98 ± 0.05**	0.13	**1.12 ± 0.05**	**1.21 ± 0.05**	**0.70 ± 0.02**	**0.40 ± 0.01**	**1.01 ± 0.06**	**1.24 ± 0.05**
S110	At2g42600	Phosphoenolpyruvate carboxylase 2	112	109.8 / 60.0	5.6 / 5.1	0.20	**0.96 ± 0.04**	**0.99 ± 0.06**	**0.65 ± 0.03**	**1.05 ± 0.07**	**0.98 ± 0.05**	**0.97 ± 0.03**	0.15	**1.22 ± 0.06**	**0.93 ± 0.09**	**0.92 ± 0.03**	**0.79 ± 0.06**	**1.24 ± 0.13**	**1.14 ± 0.07**

Table S5. Complete list of identified proteins regulated by individual HS treatments in wild-type (Col-0) and activated *ipt* transformant roots. Molecular Weight (MW; theoretical, theo; experimental, exp.); Isoelectric point (pI; theoretical, theo; experimental, exp.); Relative spot volume (V%); Relative spot volume change.

Spot N.	AGI (TAIR)	Name (UniProt)	Score	MW	pI	Wild type (Col-0)							Plants with increased CK levels (11.5)						
							40° C heat only to shoots		40° C heat to shoots&roots		40° C heat to roots		0	40° C heat only to shoot		40° C heat to shoots&roots		40° C heat only to roots	
				(theo./exp.)	(theo./exp.)	V%	30 min	180 min	30 min	180 min	30 min	180 min	V%	30 min	180 min	30 min	180 min	30 min	180 min
R01	At5g26130	Hypotetical protein, SCP family protein	312	18.4 / 18.0	8.2 / 7.0	0.34	**0.94 ± 0.18**	**0.46 ± 0.06**	**0.98 ± 0.46**	**0.74 ± 0.22**	**0.64 ± 0.14**	**0.61 ± 0.24**	0.36	**0.68 ± 0.07**	**0.77 ± 0.03**	**0.90 ± 0.16**	**0.72 ± 0.03**	**0.86 ± 0.02**	**0.96 ± 0.04**
R02	At5g17920	5-methyltetrahydropteroyltriglutamate--homocysteine methyltransferase	787	84.4 / 80.0	6.1 / 6.8	0.15	**1.33 ± 0.16**	**1.74 ± 0.23**	**0.87 ± 0.05**	**1.08 ± 0.24**	**1.37 ± 0.17**	**2.03 ± 0.36**	0.15	**0.71 ± 0.27**	**0.64 ± 0.09**	**0.71 ± 0.01**	**0.58 ± 0.37**	**1.20 ± 0.00**	**0.82 ± 0.10**
R03	At5g02500	Heat shock cognate 70 kDa protein 1	1090	71.4 / 70.0	5.0 / 5.0	0.70	**0.94 ± 0.04**	**1.49 ± 0.07**	**0.94 ± 0.14**	**1.15 ± 0.11**	**1.06 ± 0.26**	**1.28 ± 0.09**	0.70	**1.09 ± 0.22**	**0.96 ± 0.10**	**0.88 ± 0.06**	**0.92 ± 0.07**	**1.04 ± 0.24**	**1.23 ± 0.19**
R04	At4g37910	Mitochondrial heat shock protein 70-1	581	73.0 / 70.0	5.5 / 5.2	0.08	**1.13 ± 0.06**	**2.84 ± 0.42**	**0.83 ± 0.14**	**1.12 ± 0.08**	**1.40 ± 0.20**	**1.23 ± 0.01**	0.08	**0.89 ± 0.27**	**0.99 ± 0.06**	**1.05 ± 0.28**	**0.78 ± 0.12**	**1.16 ± 0.15**	**1.22 ± 0.08**
R05	At3g09260	Beta-glucosidase 23	781	59.7 / 60.0	6.5 / 7.0	0.50	**0.60 ± 0.30**	**0.48 ± 0.39**	**0.92 ± 0.09**	**0.73 ± 0.11**	**1.10 ± 0.29**	**0.84 ± 0.31**	0.50	**1.03 ± 0.15**	**1.22 ± 0.11**	**0.75 ± 0.24**	**0.52 ± 0.06**	**0.65 ± 0.29**	**0.58 ± 0.27**
R06	At3g09260	Beta-glucosidase 23	638	59.7 / 60.0	6.5 / 7.1	0.35	**0.49 ± 0.06**	**0.49 ± 0.02**	**0.87 ± 0.09**	**0.74 ± 0.09**	**0.92 ± 0.40**	**0.66 ± 0.35**	0.24	**1.27 ± 0.04**	**1.10 ± 0.23**	**0.82 ± 0.09**	**0.69 ± 0.14**	**0.82 ± 0.08**	**0.85 ± 0.13**
R07	At3g23990	Chaperonin CPN60, mitochondrial	569	61.2 / 60.0	5.7 / 5.3	0.25	**0.94 ± 0.15**	**1.13 ± 0.29**	**0.83 ± 0.07**	**1.30 ± 0.16**	**1.72 ± 0.42**	**1.83 ± 0.22**	0.23	**0.56 ± 0.22**	**0.68 ± 0.15**	**0.70 ± 0.16**	**0.78 ± 0.04**	**1.66 ± 0.46**	**1.88 ± 0.40**
R08	At1g24510	T-complex protein 1 subunit epsilon	373	59.4 / 60.0	5.5 / 5.5	0.12	**1.03 ± 0.19**	**1.57 ± 0.34**	**0.76 ± 0.28**	**0.71 ± 0.08**	**1.39 ± 0.10**	**0.98 ± 0.20**	0.11	**0.82 ± 0.20**	**0.78 ± 0.14**	**0.63 ± 0.09**	**0.54 ± 0.15**	**1.13 ± 0.07**	**0.97 ± 0.22**
R09	At4g13930	Serine hydroxymethyltransferase 4	112	51.7 / 50.0	6.8 / 7.0	0.08	**0.83 ± 0.19**	**0.48 ± 0.32**	**1.12 ± 0.37**	**0.95 ± 0.18**	**0.95 ± 0.25**	**0.69 ± 0.02**	0.08	**0.81 ± 0.03**	**0.90 ± 0.07**	**0.85 ± 0.16**	**1.10 ± 0.10**	**0.68 ± 0.29**	**0.64 ± 0.05**
R10	At3g17390	S-adenosylmethionine synthase 4	862	42.8 / 40.0	5.5 / 5.6	0.24	**0.80 ± 0.09**	**1.18 ± 0.16**	**1.09 ± 0.21**	**0.95 ± 0.32**	**1.29 ± 0.21**	**2.08 ± 0.15**	0.24	**0.70 ± 0.25**	**0.73 ± 0.07**	**1.26 ± 0.37**	**0.75 ± 0.07**	**1.54 ± 0.26**	**1.25 ± 0.15**
R11	At3g17820	Glutamine synthetase cytosolic isozyme 1-3	402	38.6 / 40.0	5.7 / 5.6	0.20	**0.87 ± 0.20**	**1.02 ± 0.22**	**1.41 ± 0.05**	**0.93 ± 0.15**	**1.43 ± 0.07**	**1.60 ± 0.00**	0.20	**0.76 ± 0.00**	**0.71 ± 0.10**	**1.08 ± 0.06**	**1.16 ± 0.05**	**1.29 ± 0.09**	**1.24 ± 0.09**
R12	At1g79550	Phosphoglycerate kinase	742	42.1 / 40.0	5.5 / 5.5	0.30	**0.90 ± 0.07**	**1.19 ± 0.06**	**1.14 ± 0.10**	**1.11 ± 0.05**	**1.32 ± 0.23**	**1.50 ± 0.06**	0.30	**0.76 ± 0.02**	**0.74 ± 0.07**	**0.90 ± 0.07**	**1.06 ± 0.02**	**1.11 ± 0.06**	**1.00 ± 0.07**
R13	At2g44060	Late embryogenesis abundant family protein	557	35.9 / 40.0	4.7 / 4.7	0.13	**1.01 ± 0.06**	**1.43 ± 0.08**	**1.00 ± 0.14**	**1.07 ± 0.16**	**1.18 ± 0.08**	**1.31 ± 0.39**	0.15	**0.90 ± 0.08**	**0.67 ± 0.12**	**1.09 ± 0.17**	**0.97 ± 0.08**	**1.27 ± 0.07**	**1.17 ± 0.20**
R14	At3g02230	Reversibly glycosylated polypeptide-1	288	40.7 / 40.0	5.6 / 5.6	0.29	**0.84 ± 0.18**	**0.95 ± 0.20**	**1.37 ± 0.17**	**0.95 ± 0.10**	**1.26 ± 0.22**	**1.53 ± 0.06**	0.39	**0.82 ± 0.10**	**0.74 ± 0.04**	**1.05 ± 0.04**	**1.18 ± 0.04**	**1.09 ± 0.07**	**0.79 ± 0.07**
R15	At2g01140	Probable fructose-bisphosphate aldolase 3, chloroplastic	536	42.3 / 40.0	8.2 / 6.7	0.30	**1.05 ± 0.07**	**0.65 ± 0.11**	**0.93 ± 0.18**	**0.90 ± 0.14**	**0.79 ± 0.13**	**0.96 ± 0.16**	0.30	**1.04 ± 0.01**	**0.89 ± 0.07**	**0.81 ± 0.29**	**0.98 ± 0.08**	**1.17 ± 0.24**	**0.92 ± 0.06**
R16	At1g35160	14-3-3-like protein GF14 phi	465	30.2 / 30.0	4.8 / 4.8	0.17	**0.91 ± 0.23**	**0.91 ± 0.18**	**0.76 ± 0.31**	**0.97 ± 0.03**	**1.14 ± 0.26**	**1.19 ± 0.22**	0.17	**0.66 ± 0.20**	**0.56 ± 0.20**	**0.92 ± 0.11**	**1.06 ± 0.15**	**1.10 ± 0.10**	**0.96 ± 0.27**
R17	At1g70410	Carbonic anhydrase, putative	415	30.8 / 25.0	6.7 / 6.9	0.22	**0.65 ± 0.05**	**0.69 ± 0.11**	**0.77 ± 0.21**	**1.24 ± 0.10**	**0.87 ± 0.26**	**0.74 ± 0.00**	0.20	**0.79 ± 0.23**	**0.71 ± 0.17**	**1.12 ± 0.24**	**1.38 ± 0.03**	**0.94 ± 0.16**	**0.84 ± 0.31**
R18	At3g02780	Isopentenyl-diphosphate Delta-isomerase II, chloroplastic	433	32.6 / 25.0	6.1 / 5.2	0.09	**1.32 ± 0.06**	**1.39 ± 0.12**	**1.19 ± 0.07**	**1.46 ± 0.11**	**0.89 ± 0.13**	**1.05 ± 0.04**	0.10	**1.54 ± 0.01**	**1.15 ± 0.05**	**0.92 ± 0.06**	**1.01 ± 0.22**	**1.15 ± 0.17**	**1.57 ± 0.14**
R19	At3g55440	Triosephosphate isomerase, cytosolic	428	27.2 / 25.0	5.4 / 5.4	0.15	**1.39 ± 0.05**	**1.48 ± 0.16**	**1.08 ± 0.13**	**1.46 ± 0.03**	**1.05 ± 0.25**	**0.97 ± 0.20**	0.15	**1.24 ± 0.22**	**1.47 ± 0.13**	**1.08 ± 0.07**	**1.11 ± 0.01**	**0.97 ± 0.06**	**0.87 ± 0.29**
R20	At4g14630	Germin-like protein subfamily 1 member 8	314	23.6 / 24.0	6.3 / 5.9	0.20	**1.42 ± 0.29**	**1.42 ± 0.07**	**0.80 ± 0.16**	**0.73 ± 0.11**	**1.67 ± 0.56**	**1.15 ± 0.11**	0.30	**1.00 ± 0.06**	**0.98 ± 0.01**	**0.89 ± 0.17**	**0.64 ± 0.23**	**0.69 ± 0.06**	**0.66 ± 0.27**
R21	At4g25100	Superoxide dismutase [Fe], chloroplastic	198	23.8 / 24.0	6.1 / 6.1	0.22	**1.45 ± 0.31**	**1.17 ± 0.32**	**1.32 ± 0.06**	**0.99 ± 0.23**	**0.82 ± 0.50**	**0.60 ± 0.07**	0.28	**0.80 ± 0.13**	**0.91 ± 0.14**	**0.83 ± 0.15**	**0.96 ± 0.27**	**0.67 ± 0.15**	**0.59 ± 0.40**
R22	At2g21660	Glycine-rich RNA-binding protein 7	549	16.9 / 18.0	5.9 / 5.5	0.47	**0.80 ± 0.18**	**0.70 ± 0.26**	**0.64 ± 0.20**	**0.53 ± 0.46**	**0.68 ± 0.01**	**0.69 ± 0.23**	0.47	**0.70 ± 0.02**	**0.69 ± 0.12**	**0.80 ± 0.10**	**0.71 ± 0.23**	**0.97 ± 0.17**	**0.72 ± 0.08**
R23	At4g33720	Pathogenesis-related protein, SCP family protein	141	18.5 / 18.0	6.1 / 5.1	0.40	**1.10 ± 0.24**	**0.63 ± 0.16**	**1.24 ± 0.06**	**0.69 ± 0.14**	**0.84 ± 0.43**	**0.69 ± 0.14**	0.55	**1.01 ± 0.07**	**1.10 ± 0.18**	**0.73 ± 0.17**	**0.52 ± 0.47**	**1.12 ± 0.10**	**0.92 ± 0.26**
R24	At2g05710	Aconitate hydratase 2, mitochondrial	781	108.2 / 100.0	6.7 / 6.1	0.22	**1.13 ± 0.04**	**1.14 ± 0.11**	**0.96 ± 0.25**	**1.14 ± 0.06**	**0.90 ± 0.38**	**1.31 ± 0.04**	0.26	**1.30 ± 0.12**	**1.39 ± 0.07**	**1.03 ± 0.19**	**1.06 ± 0.08**	**0.50 ± 0.38**	**0.92 ± 0.24**

R25	At1g66280	Beta-glucosidase 22	374	59.8 / 60.0	6.7 / 7.5	0.34	0.54 ± 0.63	0.55 ± 0.32	0.96 ± 0.24	0.87 ± 0.27	1.01 ± 0.00	0.86 ± 0.01	0.49	0.95 ± 0.25	0.84 ± 0.04	1.97 ± 0.50	1.62 ± 0.10	0.82 ± 0.02	0.81 ± 0.02
R26	At1g78850	Curculin-like (mannose-binding) lectin family protein	345	49.1 / 50.0	7.8 / 7.8	0.32	0.64 ± 0.25	0.69 ± 0.15	0.94 ± 0.12	0.80 ± 0.15	0.76 ± 0.22	0.78 ± 0.02	0.29	0.73 ± 0.35	0.82 ± 0.05	1.07 ± 0.16	0.96 ± 0.14	0.77 ± 0.36	0.83 ± 0.08
R27	At5g46290	3-oxoacyl-[acyl-carrier-protein] synthase I, chloroplastic	501	50.4 / 40.0	8.3 / 6.8	0.12	1.02 ± 0.22	0.83 ± 0.01	0.74 ± 0.21	0.70 ± 0.09	0.94 ± 0.21	0.99 ± 0.17	0.12	1.03 ± 0.04	1.01 ± 0.05	0.69 ± 0.08	0.72 ± 0.15	1.30 ± 0.10	1.11 ± 0.09
R28	At4g11260	Protein SGT1 homolog B	364	39.8 / 40.0	5.0 / 5.0	0.05	1.14 ± 0.03	1.29 ± 0.04	1.12 ± 0.09	1.60 ± 0.20	1.00 ± 0.12	0.77 ± 0.03	0.06	1.20 ± 0.20	1.14 ± 0.14	1.75 ± 0.33	1.56 ± 0.36	1.11 ± 0.21	1.26 ± 0.37
R29	At3g13235	Ubiquitin family protein	458	45.4 / 40.0	4.8 / 4.9	0.08	1.09 ± 0.31	0.88 ± 0.22	0.70 ± 0.03	1.07 ± 0.12	0.98 ± 0.00	1.11 ± 0.09	0.10	1.14 ± 0.06	1.18 ± 0.02	0.98 ± 0.14	0.97 ± 0.09	1.29 ± 0.09	0.94 ± 0.14
R30	At1g77120	Alcohol dehydrogenase class-P	692	41.2 / 40.0	5.8 / 6.0	0.13	1.39 ± 0.14	1.21 ± 0.16	1.13 ± 0.03	1.23 ± 0.16	0.90 ± 0.06	1.23 ± 0.07	0.20	1.18 ± 0.02	1.08 ± 0.08	1.23 ± 0.30	2.25 ± 0.42	1.17 ± 0.17	1.12 ± 0.12
R31	At4g39660	Alanine--glyoxylate aminotransferase 2 homolog 1, mitochondrial	633	52.0 / 40.0	7.7 / 7.3	0.11	0.88 ± 0.17	0.98 ± 0.11	0.77 ± 0.01	0.61 ± 0.08	1.03 ± 0.20	1.03 ± 0.23	0.11	0.78 ± 0.25	0.95 ± 0.15	0.76 ± 0.20	0.75 ± 0.06	1.00 ± 0.30	1.00 ± 0.30
R32	At5g38940	Germin-like protein subfamily 1 member 11	406	23.7 / 24.0	8.6 / 8.6	0.65	1.13 ± 0.13	0.80 ± 0.04	1.15 ± 0.08	0.88 ± 0.13	0.93 ± 0.09	0.60 ± 0.14	0.68	1.00 ± 0.03	0.89 ± 0.16	1.21 ± 0.06	0.99 ± 0.06	0.90 ± 0.00	0.75 ± 0.24
R33	At1g09780	2,3-bisphosphoglycerate-independent phosphoglycerate mutase 1	849	60.6 / 65.0	5.3 / 5.4	0.22	1.11 ± 0.00	1.16 ± 0.03	0.88 ± 0.03	0.99 ± 0.00	1.09 ± 0.29	1.56 ± 0.08	0.22	0.86 ± 0.10	0.82 ± 0.02	0.83 ± 0.06	0.88 ± 0.02	0.86 ± 0.03	1.59 ± 0.19
R34	N/A	**Not identified**	N/A	N/A / 60.0	N/A / 5.6	0.15	0.83 ± 0.37	0.97 ± 0.22	1.13 ± 0.07	0.91 ± 0.06	1.16 ± 0.26	1.49 ± 0.10	0.16	1.06 ± 0.05	1.15 ± 0.12	0.96 ± 0.24	0.93 ± 0.22	1.57 ± 0.04	1.63 ± 0.04
R35	At4g34200	Phosphoglycerate dehydrogenase-like protein	168	63.3 / 60.0	6.2 / 5.9	0.35	1.05 ± 0.04	1.04 ± 0.05	0.86 ± 0.24	1.07 ± 0.19	1.27 ± 0.17	1.99 ± 0.23	0.35	0.92 ± 0.30	0.77 ± 0.19	0.91 ± 0.16	0.97 ± 0.22	1.31 ± 0.08	1.40 ± 0.20
R35	At3g58610	Ketol-acid reductoisomerase, chloroplastic	330	63.8 / 60.0	6.4 / 5.9	0.35	1.05 ± 0.04	1.04 ± 0.05	0.86 ± 0.24	1.07 ± 0.19	1.27 ± 0.17	1.99 ± 0.23	0.35	0.92 ± 0.30	0.77 ± 0.19	0.91 ± 0.16	0.97 ± 0.22	1.31 ± 0.08	1.40 ± 0.20
R36	At4g13940	Adenosylhomocysteinase 1	595	53.4 / 50.0	5.7 / 5.8	0.60	1.14 ± 0.22	1.29 ± 0.18	1.12 ± 0.07	1.01 ± 0.04	1.25 ± 0.32	1.64 ± 0.06	0.59	0.92 ± 0.10	0.87 ± 0.09	1.24 ± 0.08	1.23 ± 0.06	1.59 ± 0.40	1.36 ± 0.02

Table S6. Number of HS-regulated proteins associated with specific biological functions in wild-type (Col-0) and transformant (ipt). Assignment according to databases Uniprot and TAIR.

Number of regulated proteins						Description - Biological function (TAIR)
S (*Col-0l*)	S (*ipt*)	SR (*Col-0)*)	SR (*ipt*)	R (*Col-0*)-0	R (*ipt*)	
37	36	45	45	36	36	**response to stimulus**
26	26	30	26	28	28	**metabolic process**
26	25	32	34	27	27	**response to chemical stimulus**
25	24	30	28	28	28	**cellular process**
25	23	32	31	22	22	**response to stress**
22	21	27	30	23	23	**response to inorganic substance**
20	21	27	23	22	22	**cellular metabolic process**
20	21	27	29	22	22	**response to metal ion**
18	20	24	26	21	21	**response to cadmium ion**
16	14	25	22	18	18	**response to abiotic stimulus**
12	11	15	15	10	10	**defense response**
12	11	14	13	9	9	**multi-organism process**
11	11	14	13	8	8	**response to biotic stimulus**
11	11	14	13	8	8	**response to other organism**
10	8	11	8	10	10	**cellular nitrogen compound metabolic process**
10	8	11	X	10	10	**nitrogen compound metabolic process**
10	10	11	10	7	7	**response to bacterium**
9	10	9	9	7	7	**defense response to bacterium**
8	5	11	9	9	9	**response to cold**
8	5	8	8	6	6	**response to endogenous stimulus**
8	5	8	8	6	6	**response to hormone stimulus**
8	X	8	8	X	X	**response to organic substance**
8	8	14	10	10	10	**response to osmotic stress**
8	8	14	10	10	10	**response to salt stress**
8	5	13	11	9	9	**response to temperature stimulus**
8	8	10	X	11	11	**small molecule metabolic process**
7	5	6	8	X	X	**cellular response to stimulus**
7	5	8	7	6	6	**response to abscisic acid stimulus**
6	9	10	9	7	7	**catabolic process**
6	3	6	8	X	X	**response to oxidative stress**
5	5	6	5	5	5	**carboxylic acid metabolic process**
5	5	6	5	5	5	**cellular ketone metabolic process**
5	4	4	7	X	X	**cellular response to chemical stimulus**
5	5	6	5	5	5	**organic acid metabolic process**
5	5	6	5	5	5	**oxoacid metabolic process**
5	5	6	5	5	5	**sulfur metabolic process**
4	4	6	5	4	4	**amine metabolic process**
4	4	6	5	4	4	**cellular amine metabolic process**
4	4	6	5	4	4	**cellular amino acid and derivative metabolic process**
4	4	6	5	4	4	**cellular amino acid metabolic process**

4	5	7	7	X	X	**cellular catabolic process**
4	X	5	X	4	4	**cellular nitrogen compound biosynthetic process**
4	X	4	5	X	X	**cellular response to stress**
4	X	5	5	X	X	**immune response**
4	X	5	5	X	X	**immune system process**
4	X	5	5	3	3	**innate immune response**
4	X	4	4	X	X	**response to light stimulus**
4	X	4	4	X	X	**response to radiation**
4	4	7	6	5	5	**response to zinc ion**
4	6	5	4	6	6	**small molecule catabolic process**
3	6	4	4	5	5	**carbohydrate catabolic process**
3	6	4	4	5	5	**cellular carbohydrate catabolic process**
3	X	X	X	X	X	**cellular response to endogenous stimulus**
3	X	X	3	X	X	**cellular response to hormone stimulus**
3	2	2	3	X	X	**circadian rhythm**
3	3	X	X	4	4	**cofactor metabolic process**
3	X	2	2	3	3	**regulation of stomatal movement**
3	2	3	5	X	X	**response to reactive oxygen species**
3	2	2	3	X	X	**rhythmic process**
3	2	3	3	2	2	**serine family amino acid metabolic process**
3	2	4	3	3	3	**sulfur compound biosynthetic process**
2	4	3	3	2	2	**amine catabolic process**
2	X	X	X	1	1	**carbon utilization**
2	2	2	2	2	2	**carboxylic acid catabolic process**
2	2	2	2	2	2	**cellular amino acid catabolic process**
2	2	2	2	2	2	**cellular response to abscisic acid stimulus**
2	2	2	3	X	X	**cellular response to oxidative stress**
2	2	1	2	X	X	**cellular response to oxygen radical**
2	2	2	3	X	X	**cellular response to reactive oxygen species**
2	2	1	2	X	X	**cellular response to superoxide**
2	1	2	2	X	X	**cysteine biosynthetic process**
2	1	2	2	X	X	**cysteine metabolic process**
2	X	X	X	X	X	**defense response to fungus, incompatible interaction**
2	4	3	2	4	4	**alcohol catabolic process**
2	4	2	X	4	4	**glucose catabolic process**
2	4	3	2	4	4	**glucose metabolic process**
2	4	3	2	4	4	**hexose catabolic process**
2	4	3	X	5	5	**hexose metabolic process**
2	2	X	X	X	X	**chloroplast organization**
2	4	3	2	4	4	**monosaccharide catabolic process**
2	3	X	X	3	3	**NADP metabolic process**
2	3	X	X	3	3	**nicotinamide nucleotide metabolic process**
2	2	2	2	2	2	**organic acid catabolic process**
2	3	X	X	3	3	**oxidoreduction coenzyme metabolic process**
2	2	2	3	X	X	**oxygen and reactive oxygen species metabolic process**
2	3	X	X	3	3	**pyridine nucleotide metabolic process**

2	2	1	2	X	X	**removal of superoxide radicals**
2	2	X	2	X	X	**response to copper ion**
2	2	1	2	X	X	**response to oxygen radical**
2	2	1	2	X	X	**response to superoxide**
2	1	2	2	X	X	**serine family amino acid biosynthetic process**
2	2	3	3	2	2	**sulfur amino acid biosynthetic process**
2	2	3	3	2	2	**sulfur amino acid metabolic process**
2	2	1	2	X	X	**superoxide metabolic process**
1	1	1	1	X	X	**cysteine biosynthetic process from serine**
1	1	1	1	1	1	**glycine decarboxylation via glycine cleavage system**
1	X	X	X	X	X	**isocitrate metabolic process**
1	X	1	1	1	1	**mRNA export from nucleus**
1	X	1	1	1	1	**mRNA transport**
1	X	1	X	1	1	**oxazole or thiazole biosynthetic process**
1	X	1	X	1	1	**oxazole or thiazole metabolic process**
1	1	1	1	1	1	**proline catabolic process**
1	1	1	1	1	1	**proline catabolic process to glutamate**
1	1	1	1	1	1	**proline metabolic process**
1	X	X	X	1	1	**response to carbon dioxide**
1	X	1	X	X	X	**response to mannitol stimulus**
1	X	3	1	1	1	**RNA secondary structure unwinding**
1	1	1	X	1	1	**sulfate reduction**
1	X	1	X	1	1	**thiamin and derivative biosynthetic process**
1	X	1	X	1	1	**thiamin and derivative metabolic process**
1	X	1	X	1	1	**thiamin biosynthetic process**
1	X	1	X	1	1	**thiamin metabolic process**
X	X	X	X	5	5	**alcohol metabolic process**
X	X	4	X	X	X	**amine biosynthetic process**
X	X	2	2	X		**calcium ion homeostasis**
X	7	X	X	7	7	**carbohydrate metabolic process**
X	2	2	2	2	2	**carbon fixation**
X	X	4	X	X	X	**carboxylic acid biosynthetic process**
X	X	2	2	X	X	**cation homeostasis**
X	X	X	X	2	2	**cell tip growth**
X	2	4	3	X	X	**cellular amino acid biosynthetic process**
X	7	5	5	7	7	**cellular carbohydrate metabolic process**
X	X	2	2	X	X	**cellular glucan metabolic process**
X	X	X	X	2	2	**cellular metabolic compound salvage**
X	X	2	2	X	X	**cellular polysaccharide biosynthetic process**
X	X	1	1	X	X	**cellular response to hydrogen peroxide**
X	X	1	1	X	X	**cellular response to nitrogen starvation**
X	3	X	X	3	3	**coenzyme metabolic process**
X	X	X	X	2	2	**developmental cell growth**
X	1	X	X	X	X	**defense response to insect**
X	X	X	2	X	X	**detection of abiotic stimulus**
X	X	X	2	X	X	**detection of external stimulus**

X	X	1	1	X	X	**detection of gravity**
X	X	X	2	X	X	**detection of stimulus**
X	X	2	2	X	X	**di-, tri-valent inorganic cation homeostasis**
X	X	2	2	X	X	**disaccharide biosynthetic process**
X	X	2	2	2	2	**disaccharide metabolic process**
X	X	X	X	2	2	**electron transport chain**
X	X	X	X	4	4	**embryonic development**
X	X	X	X	1	1	**fructose 1,6-bisphosphate metabolic process**
X	X	X	X	1	1	**fructose metabolic process**
X	X	1	1	X	X	**galactose catabolic process**
X	3	4	X	5	5	**generation of precursor metabolites and energy**
X	X	2	2	X	X	**glucan biosynthetic process**
X	X	2	2	X	X	**glucan metabolic process**
X	X	1	1	X	X	**glucose 1-phosphate metabolic process**
X	2	X	2	X	X	**glucosinolate catabolic process**
X	2	X	2	X	X	**glucosinolate metabolic process**
X	1	X	X	X	X	**glutamate metabolic process**
X	1	X	X	X	X	**glutamine family amino acid catabolic process**
X	1	X	X	X	X	**glycine catabolic process**
X	X	X	X	2	2	**glycolysis**
X	X	2	2	X	X	**glycoside biosynthetic process**
X	2	X	2	X	X	**glycoside catabolic process**
X	3	3	4	3	3	**glycoside metabolic process**
X	2	X	2		X	**glycosinolate catabolic process**
X	2	X	2		X	**glycosinolate metabolic process**
X	X	X	3	X	X	**homeostatic process**
X	X	1	1	X	X	**hydrogen peroxide catabolic process**
X	X	1	1	X	X	**hydrogen peroxide metabolic process**
X	X	X	3	X	X	**chemical homeostasis**
X	X	2	2	X	X	**ion homeostasis**
X	1	X	X	X	X	**L-serine metabolic process**
X	1	X	X	X	X	**malate metabolic process**
X	X	2	2	X	X	**metal ion homeostasis**
X	4	3	X	5	5	**monosaccharide metabolic process**
X	3	X	X	2	2	**NADPH regeneration**
X	X	X	X	1	1	**negative regulation of photosynthesis, light reaction**
X	3	X	X	3	3	**nucleobase, nucleoside and nucleotide metabolic process**
X	3	X	X	3	3	**nucleoside phosphate metabolic process**
X	3	X	X	3	3	**nucleotide metabolic process**
X	X	2	2	X	X	**oligosaccharide biosynthetic process**
X	X	2	2	2	2	**oligosaccharide metabolic process**
X	X	4	X	X	X	**organic acid biosynthetic process**
X	2	2	2	2	2	**organic substance metabolic process**
X	4	X	X	X	X	**oxidation reduction**
X	3	X	X	2	2	**pentose-phosphate shunt**
X	1	X	X	X	X	**pentose-phosphate shunt, oxidative branch**

X	X	X	X	1	1	**photoinhibition**
X	X	X	X	2	2	**photorespiration**
X	3	3	3	4	4	**photosynthesis**
X	1	1	1	1	1	**photosynthesis, dark reaction**
X	2	2	2	3	3	**photosynthesis, light reaction**
X	X	X	X	2	2	**photosynthetic electron transport chain**
X	X	X	X	1	1	**photosystem II stabilization**
X	X	X	X	2	2	**pollen tube development**
X	X	X	X	2	2	**pollen tube growth**
X	2		X	X	X	**plastid organization**
X	X	2	2	X	X	**polysaccharide biosynthetic process**
X	X	X	6	X	X	**post-embryonic development**
X	X	3	X	X	X	**protein folding**
X	1	X	X	X	X	**protein homooligomerization**
X	1	1	1	1	1	**reductive pentose-phosphate cycle**
X	X	X	X	2	2	**regulation of cellular protein metabolic process**
X	X	X	X	1	1	**regulation of dephosphorylation**
X	X	X	1	1	1	**regulation of multicellular organism growth**
X	X	X	X	1	1	**regulation of protein amino acid dephosphorylation**
X	X	X	X	2	2	**regulation of protein metabolic process**
X	X	X	X	1	1	**regulation of protein modification process**
X	1	X	X	X	X	**response to endoplasmic reticulum stress**
X	X	X	4	X	X	**response to external stimulus**
X	X	2	3	X	X	**response to hydrogen peroxide**
X	2	X	2	X	X	**response to insect**
X	X	2	X	X	X	**response to light intensity**
X	X	3	X	X	X	**ribosome biogenesis**
X	3	X	4	X	X	**secondary metabolic process**
X	2	5	2	X	X	**S-glycoside catabolic process**
X	2	X	2	X	X	**S-glycoside metabolic process**
X	1	2	2	X	X	**starch biosynthetic process**
X	X	2	2	X	X	**starch metabolic process**
X	1	X	X	2	X	**sucrose biosynthetic process**
X	X	X	X	X	2	**sucrose metabolic process**
X	2	X	2	X	X	**sulfur compound catabolic process**
X	X	2	2	X	X	**toxin catabolic process**
X	X	2	2	X	X	**toxin metabolic process**
X	X	X	X	2	2	**tube development**

Figure S1. Content of cytokinin metabolites in leaves (A, C, E, G) and roots (B, D, F, H) of wild-type (Col-0) and activated transgenic plants (*ipt*) (pmol/g FW). The CK O-glucosides (tZOG – *trans*-zeatin O-glucoside, tZROG - *trans*-zeatin riboside O-glucoside), CK phosphates (tZRMP - *trans*-zeatin riboside phosphate, iPRMP – isopentenyladenosine phosphate, DZRMP – dihydrozeatin riboside phosphate), *cis*-zeatin O-glucosides (*cis*-zeatin (riboside) O-glucoside), CK N-glucosides (7N- and 9N-glucosides of *trans*-zeatin, isopentenyladenine and dihydrozeatin).

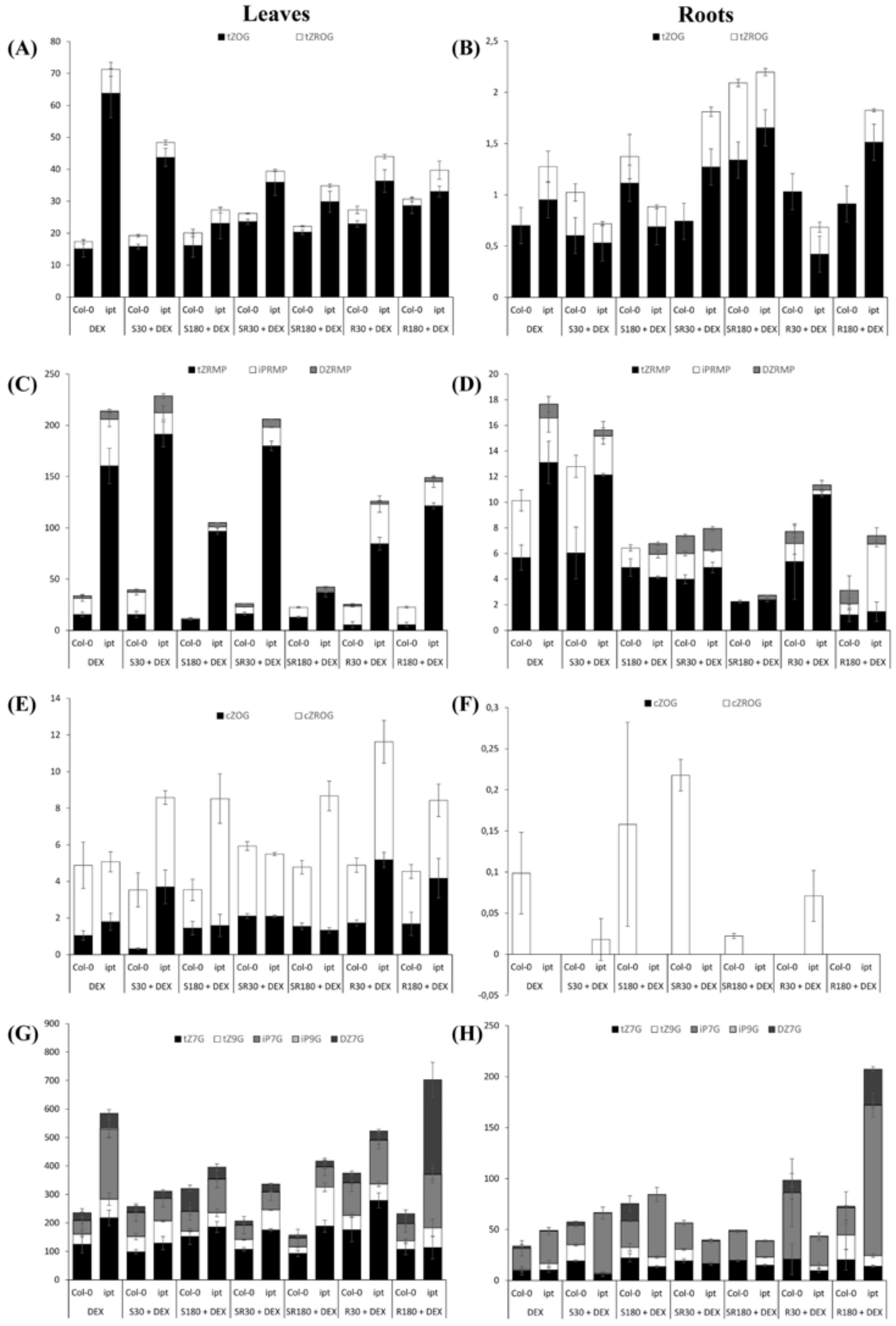

Figure S2. Stomatal conductance (g_s) in wild-type and *ipt* over-expressing transgenic tobacco leaves during heat stress. The $g_{s,}$ was measured at fully expanded leaves in a closed cabinet at 40°C at PAR 300 μmol m^{-2} s^{-1}. Three plants of each genotype were followed.

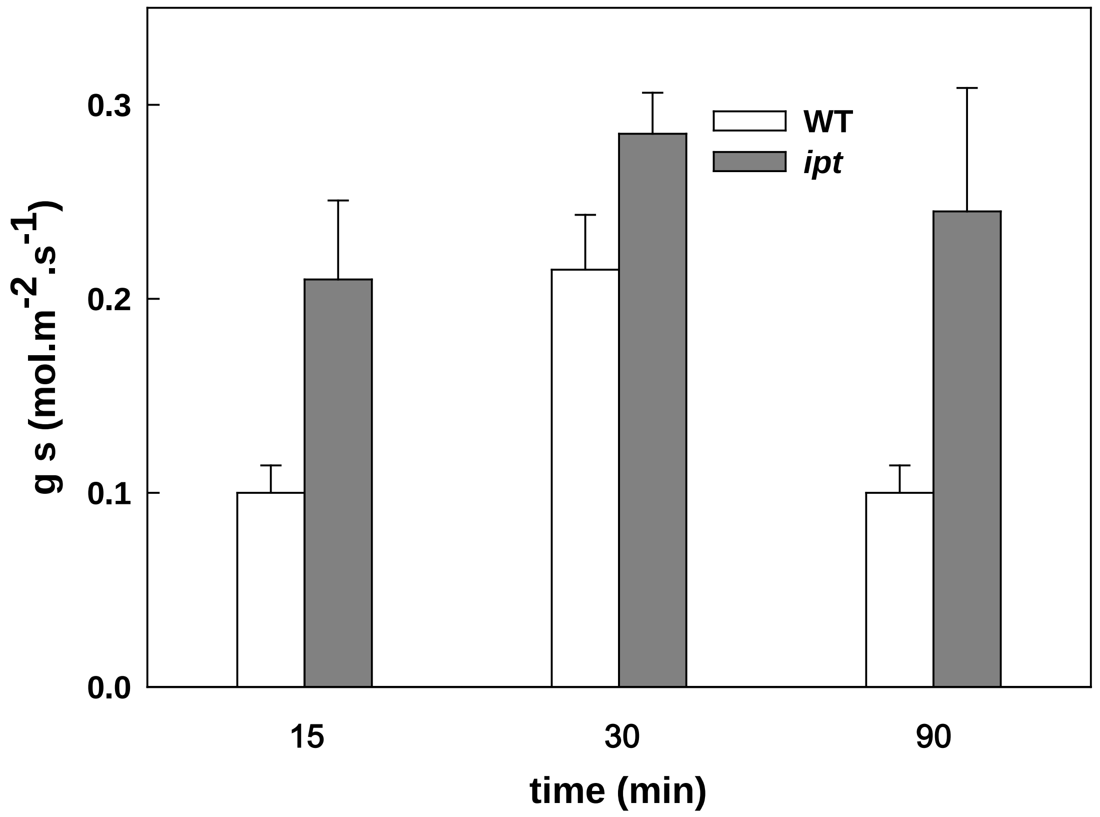

Figure S3. Representative two-dimensional gels showing protein resolution in the leaves (A) and roots (B). Differentially regulated protein spots are indicated and their numbers corespond with the lists of proteins (e.g., Supplementary Table S2). Isoelectric points (pI) and mobility of molecular mass (kDa) markers are marked. Decodon Delta 2D was used to compensate for the PAGE background and thus to optimize the visibility of all differentially regulated protein spots.

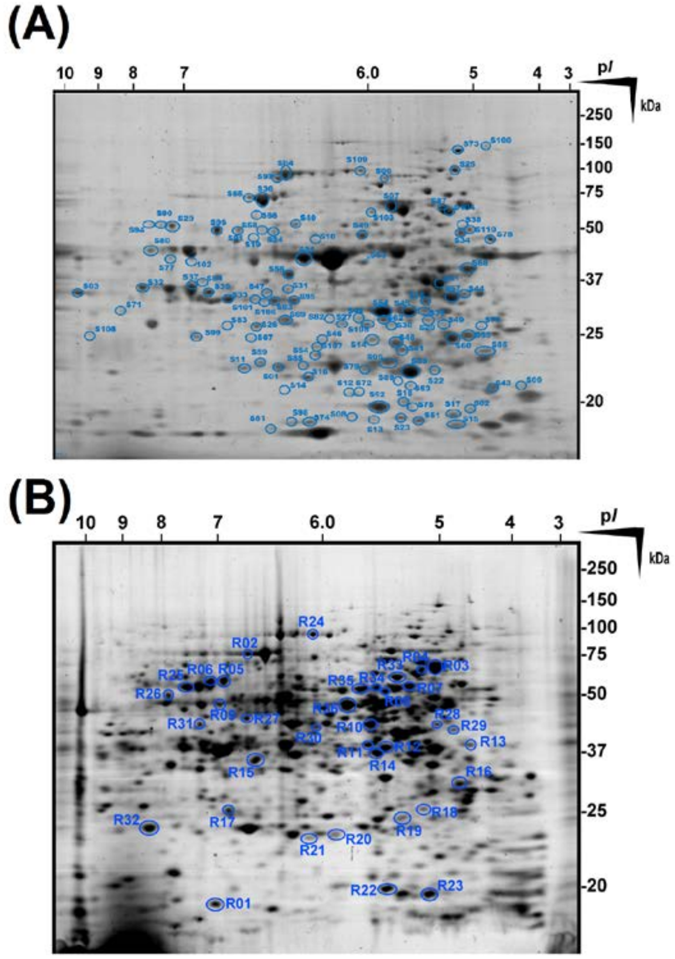

Figure S4. Subcellular localization of proteins regulated after exposition to specific HS treatments in wild-type (Col-0) (A) and transgenic plants (*ipt*) (B). Percentage diagrams show distribution of leaf proteins (diagrams above) and root proteins (diagrams below), respectively. The subcellular location of each identified protein was determined according to the TAIR database (http://www.arabidopsis.org/) and MASCP GATOR (http://gator.masc-proteomics.org/; Joshi et al., 2011).

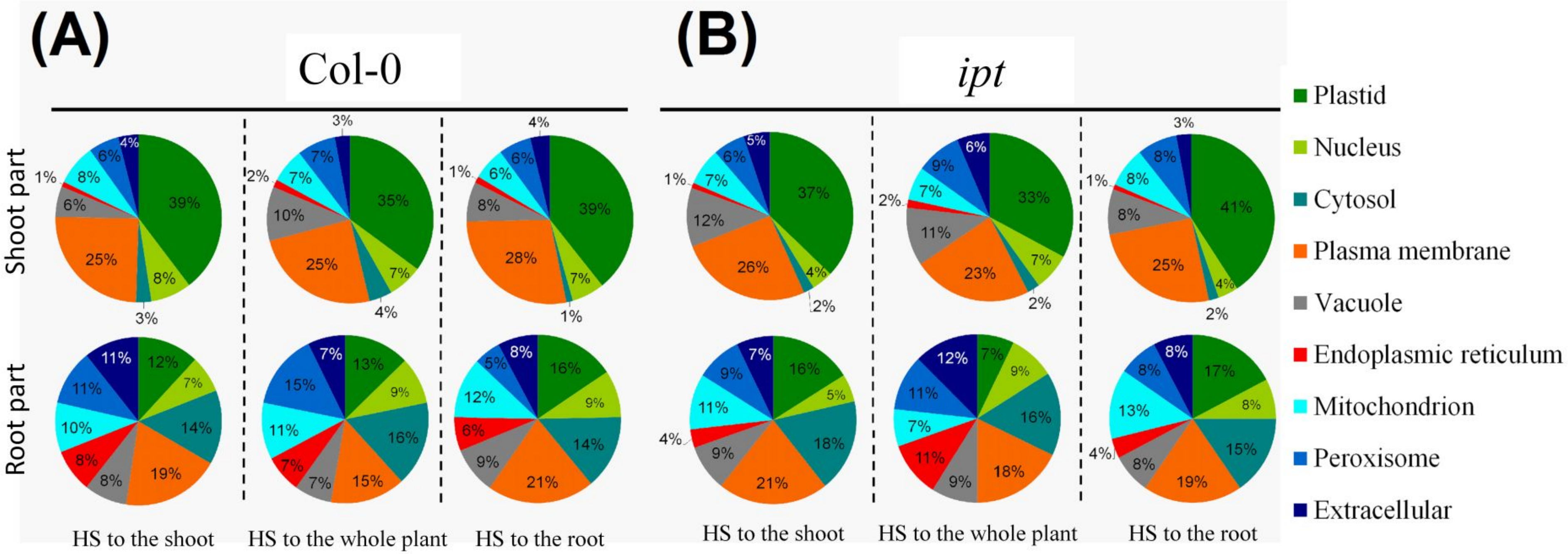

Figure S5. The heat-map of proteins regulated in the leaves and their functional assignment to temperature-stimulus response and stomatal-movement regulation (A) acquired from UNIPROT (http://www.uniprot.org/) and TAIR (http://www.arabidopsis.org/) database. The protein-protein interaction network designed by STRING database (B; www.string-db.org) demonstrates tight connection between the above mentioned biological processes. Significant comparison of protein spot volumes was based on the T-test P < 0.05, when significant relative spot volume change was made by comparison of WT 40°C/WT 21°C (for WT) and DEX 40°C ipt/40°C WT (for transgen).

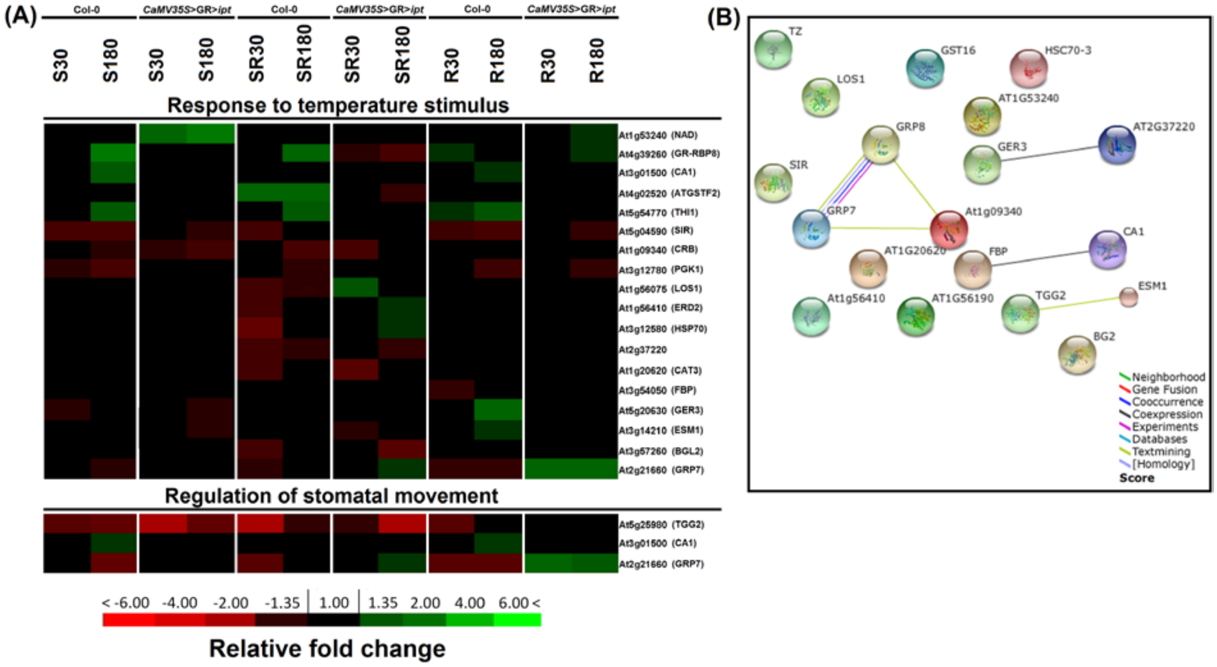

Supplement: Supplementary Data [file supp_erw129_supplementary_tables_S1_S6_Figures_S1_S5.pdf]
